# Novel Fluorescent Tetrahedral Zinc (II) Complexes Derived from 4-Phenyl-1-octyl-1*H*-imidazole Fused with Aryl-9*H*-Carbazole and Triarylamine Donor Units: Synthesis, Crystal Structures, and Photophysical Properties

**DOI:** 10.3390/ijms241512260

**Published:** 2023-07-31

**Authors:** Thompho Jason Rashamuse, Elena Mabel Coyanis, Rudolph Erasmus, Nomampondo Penelope Magwa

**Affiliations:** 1 Nanotechnology Innovation Centre, Health Platform, Advanced Materials Division, Mintek, Private Bag X3015, Randburg 2125, South Africa; mabelc@mintek.co.za; 2 Materials for Energy Research Group, Material Physics Research Institute, School of Physics, University of the Witwatersrand, Private Bag 3, Wits, Johannesburg 2050, South Africa; rudolph.erasmus@wits.ac.za; 3 Department of Chemistry, University of South Africa, Private Bag X6, Florida, Roodepoort 1710, South Africa; magwanp@unisa.ac.za

**Keywords:** Zn (II) complexes, free ligands, single X-ray chromatography, FTIR, NMR, UV-Vis absorption, photoluminescence, thermal gravimetric

## Abstract

We present here the design, synthesis, and photophysical properties of two novel fluorescent zinc (II) complexes, **ZnCl_2_**(**ImL1**)**_2_** and **ZnCl_2_**(**ImL2**)**_2_**, containing 4-(1-octyl-1*H*-imidazol-4-yl)-*N*,*N*-diphenyl-[1,1-biphenyl]-4-yl)-4-amine **ImL1** and 9-(4-(1-octyl-1*H*-imidazol-4-yl)-[1,1-biphenyl]-4-yl)-9*H*-carbazole **ImL2** ligands. The newly synthesized free ligands and their zinc (II) complexes were characterized using several spectroscopic techniques; their structures were identified by single-crystal X-ray diffraction; and their photophysical properties have been studied in the context of their chemical structure. The **ZnCl_2_**(**ImL1**)**_2_** and **ZnCl_2_**(**ImL2**)**_2_** complexes showed good thermal stability at 341 °C and 365 °C, respectively. Photophysical properties, including UV-Vis absorption spectra in ethanol solution and photoluminescence (PL) in both solid state and ethanol solution, were determined. UV-Vis adsorption data indicated that both free ligands had similar UV-Vis absorption properties, while their Zn (II) complexes had distinctive absorption characteristics. The fluorescence spectra show that both ligands and their corresponding Zn (II) complexes emit violet to cyan luminescence in the solid state at room temperature, while in ethanol solution at the same temperature, they exhibit efficient photoluminescence properties in the UV-A emission spectral region. Because of these photophysical properties, the synthesized ligands and their cognate Zn (II) complexes can be used as scaffolds for the potential development of optoelectronic materials.

## 1. Introduction

Many researchers have developed an interest in the design and synthesis of hybrid organic-inorganic systems as efficient optoelectronic materials because of their exciting potential for use in latent fingerprint (LFP) detection, organic light-emitting diodes (OLEDs), anti-counterfeiting, bio-imaging, and sensing applications [1,2,3,4,5]. The construction of these hybrid organic-inorganic systems with a fascinating range of structures and topologies is dependent on the organic ligands used and the nature of the metal ions. The careful choice of nitrogen (*N*)-containing organic ligands linked by a spacer and/or donors with a certain positional orientation is critical in building and controlling the architecture of complexes with desirable photophysical properties [6,7,8]. Several *N*-heterocyclic skeletons have been deemed building synthon ligands in organometallic and coordination chemistry because of their ability to serve as donor units, resulting in desirable properties [9,10,11,12,13]. Examples of *N*-heterocyclic systems, such as imidazole and its derivatives, have piqued the curiosity of researchers interested in developing hybrid organic-inorganic systems for various applications [2,14,15,16,17,18]. The unique properties of imidazole-based complexes, such as excellent electrochemical and photoluminescent properties, excellent thermal properties, and ease of modification, were taken into account in the design of various host materials such as electron transport, fluorescent, or phosphorescent molecules [19,20,21,22,23]. Over the years, several articles have reported the coordination of imidazole-based synthons with a variety of metal ions, including cobalt, nickel, iron, zinc, copper, and iridium, leading to the preparation of stable organic metal complexes [9,10,11,12,13,24,25,26,27]. In particular, d^10^ transition zinc (II) complexes have become the focus of an ever-growing research area owing to their attractive luminescence and the low cost of zinc metal sources [2,9,27,28,29]. This post-transition metal ion can coordinate with four donor atoms, which can easily be increased to five and six, resulting in geometry ranging from tetrahedral to trigonal bipyramid, square pyramidal, and octahedral [2,9,27,28,29]. As a result, the development of novel organic zinc (II) complexes has remained a key endeavor.

On the other hand, carbazole and diarylamine units are often regarded as important electron-donating units in donor-acceptor systems (D-A) due to their high triplet energies and good hole-transport properties [1,15,16,30,31,32,33]. The diarylamine structure consists of two benzene groups directly attached to the central nitrogen atom, while the carbazole structure is a tricyclic ring skeleton with pyrrole rings attached to two aromatic rings. These high photoluminescence efficiency, electron-rich, and inexpensive starting materials can be easily modified by substituting the central nitrogen atom [1,15,16,30,31,32,33,34,35]. Therefore, the carbazole and diarylamine frameworks remain good templates for the fabrication of OLED emitters and transparent optoelectronic materials.

In this framework, we present the synthesis, characterization, and photophysical characteristics of two novel fluorescent zinc (II) complexes, **ZnCl_2_**(**ImL1**)**_2_** and **ZnCl_2_**(**ImL_2_**)**_2_**, constructed from the ligands, 4-(1-octyl-1*H*-imidazol-4-yl)-*N*,*N*-diphenyl-[1,1-biphenyl]-4-yl)-4-amine **ImL1** and 9-(4-(1-octyl-1*H*-imidazol-4-yl)-[1,1-biphenyl]-4-yl)-9*H*-carbazole **ImL2**. The ligands were designed with an imidazole unit as the metal coordinator and two donor moieties, phenylcarbazole and triphenylamine, linked through a benzene moiety. The molecular structures of the newly synthesized free ligands and their corresponding zinc (II) complexes were analyzed and confirmed by proton (^1^H) and carbon-13 (^13^C) nuclear magnetic resonance (NMR), single-crystal X-ray diffraction, and Fourier-transform infrared spectroscopy (FTIR). The photophysical properties were also investigated employing ultraviolet-visible (UV-Vis) spectrophotometry and photoluminescent (PL) analysis.

## 2. Results and Discussion

### 2.1. Synthesis of the Free Ligands and Their Corresponding Zn(II) Complexes

**Figure 1:**
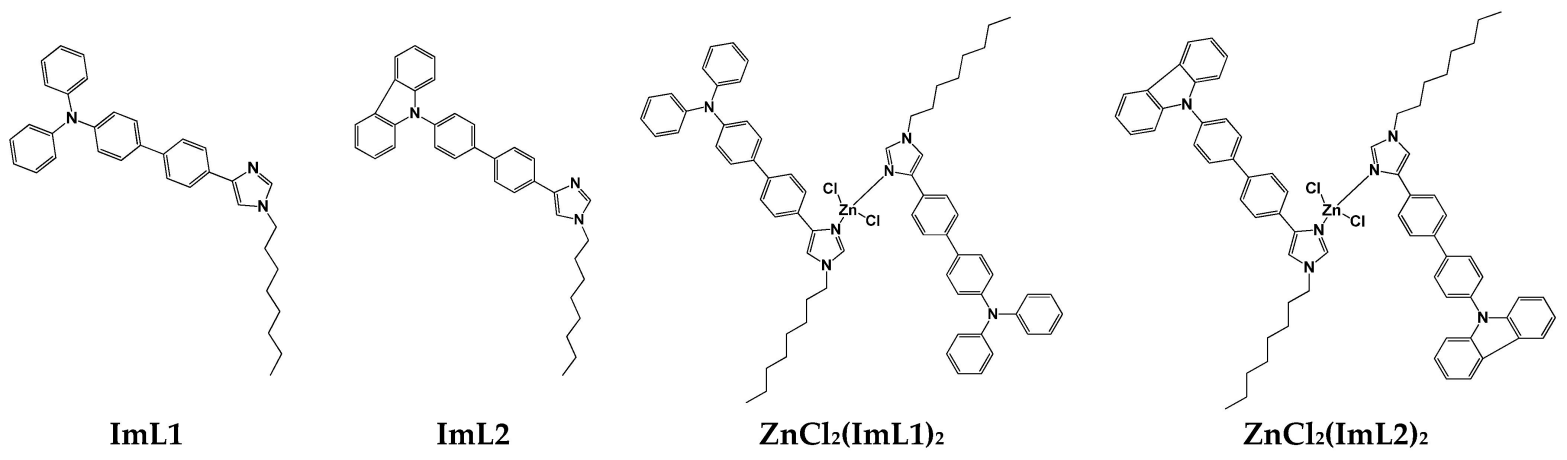
Chemical structures of the novel ligands, **ImL1** and **ImL2**, and their corresponding novel Zn (II) complexes, **ZnCl_2_**(**ImL1**)**_2_** and **ZnCl_2_**(**ImL2**)**_2_**.

**Scheme 1:**
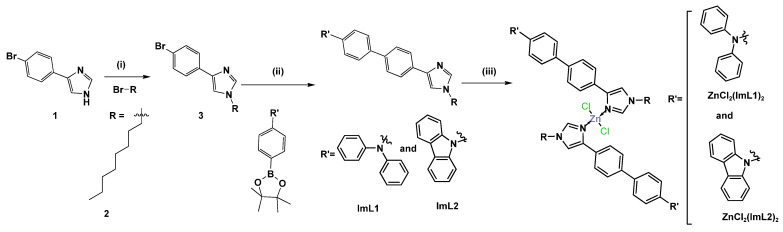
*Reagents and conditions*: (i): NaOH (50% in H_2_O), benzyltetraethylammonium chloride, toluene, 110 °C, 24 h, (ii): XPhos Pd G3 (1%) K_2_CO_3_, anhydrous dioxane, H_2_O, 100 °C, 48 h, inert argon atm, (iii) ZnCl_2_, MeOH, reflux, 24 h.

The molecular structures of the novel ligands (**ImL1** and **ImL2**) and their corresponding zinc (II) complexes, **ZnCl_2_**(**ImL1**)**_2_** and **ZnCl_2_**(**ImL2**)**_2_**, are shown in Figure 1. The desired ligands, **ImL1** and **ImL2**, were synthesized from 4-(4-bromophenyl)-1*H*-imidazole synthon in a two-step reaction (Scheme 1): the first step involved the alkylating of commercially available 4-(4-bromophenyl)-1*H*-imidazole **1** with 1-bromooctane **2** in the presence of benzyl tetraethylammonium chloride and an aqueous solution of sodium hydroxide in toluene, followed by heating at 110 °C for 18 h to obtain the 4-(4-bromophenyl)-1-octyl-1*H*-imidazole intermediate **3** with an isolated yield of 94% [36]. The second step is a Suzuki-Miyaura coupling reaction of intermediate **3** with boronic acid pinacol ester derivatives **4** and **5** catalyzed by XPhos Pd G3 in the presence of potassium carbonate in anhydrous dioxane and water under argon [37]. After 48 h of heating at 100 °C, the desired novel 4′-(1-octyl-1*H*-imidazol-4-yl)-*N*,*N*-diphenyl-[1,1′-biphenyl]-4-yl)-4-amine **ImL1** and 9-(4′-(1-octyl-1*H*-imidazol-4-yl)-[1,1′-biphenyl]-4-yl)-9*H*-carbazole **ImL2** were isolated with yields of 74% and 66%, respectively.

The Zn (II) complexes, **ZnCl_2_**(**ImL1**)**_2_** and **ZnCl_2_**(**ImL2**)**_2_**, were prepared by reacting one equivalent of ZnCl_2_ with two equivalents of the appropriate ligands **ImL1** and **ImL2**, respectively, and heating under reflux in methanol for 24 h, affording the complexes as solid materials with yields of 56% for **ZnCl_2_**(**ImL1**)**_2_** and 59% for **ZnCl_2_**(**ImL2**)**_2_** [9]. Chemical structures of the free ligands and their corresponding Zinc (II) complexes were confirmed by ^1^H NMR, ^13^C NMR, and FT-IR spectroscopy and single crystal X-ray diffraction studies of **ImL1**, **ImL2,** and **ZnCl_2_**(**ImL1**)**_2_**. The photophysical properties of the ligands and their corresponding Zn (II) complexes were also investigated by UV-Vis spectrophotometry and PL analysis.

### 2.2. NMR Characterization of the Free Ligands ImL1 and ImL2 as Well as and Their Corresponding Zn(II) Complexes ZnCl_2_(ImL)_2_

The ^1^H and ^13^C NMR spectra of both free ligands and their corresponding zinc (II) complexes supported the synthesized structures. It was found that **ImL1**, **ImL2**, and **ZnCl_2_**(**ImL1**)**_2_** were soluble in deuterated chloroform (CDCl_3_), whereas **ZnCl_2_**(**ImL2**)**_2_** was soluble in deuterated dimethyl sulfoxide (DMSO-*d*_6_). The total number of protons and carbons in the NMR spectra was congruent with the proposed title molecules; however, the proton spectra of the **ImL1** and complex **ZnCl_2_**(**ImL1**)**_2_** showed different chemical shift signatures. The proton spectrum of the free ligand **ImL1** in Figure S1 showed a signal from the 2-methine proton of the imidazole moiety overlapping between 7.48 and 7.50 ppm; this proton becomes downshifted to a chemical shift of 8.12 ppm upon zinc coordination in the **ZnCl_2_**(**ImL1**)**_2_** spectrum (Figure S5). In addition, the 4-methine proton signal of the imidazole nucleus was also shown to overlap in the region of 6.98–7.02 ppm, which also shifts to 6.63 ppm as a result of zinc (II) ion coordination. The carbon-13 spectra of **ImL1** and **ZnCl_2_**(**ImL1**)**_2_** had no significant chemical shift difference (Figures S2 and S6 in Supplementary Materials). In the case of **ImL2** and **ZnCl_2_**(**ImL2**)**_2_**, their solubility in different deuterated NMR solvents was a notable distinguishing feature. These NMR data demonstrate unequivocally that the ligands were effectively coordinated with the zinc (II) ion (Figures S1–S8).

### 2.3. Fourier Transform Infrared Spectroscopy (FT-IR) Characterization of the Free Ligands ImL1 and ImL2 as Well as and Their Corresponding Zn(II) Complexes ZnCl_2_(ImL)_2_

**Figure 2:**
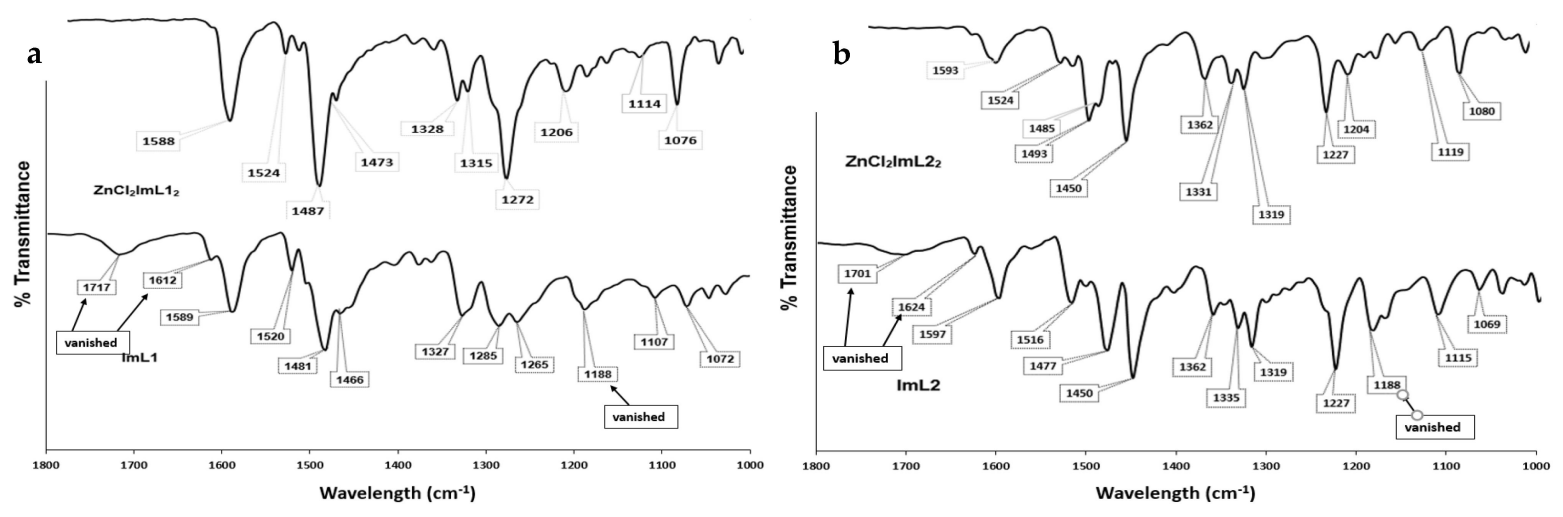
Stack FTIR spectra of (**a**) the ligand **ImL1** and **ZnCl_2_**(**ImL1**)**_2_** complex and (**b**) the ligand **ImL2** and **ZnCl_2_**(**ImL2**)**_2_** in a frequency range of 1000–1800 cm^−1^.

The FTIR spectra of the metal complexes were compared to those of the free ligands to investigate the structure of the zinc (II) complexes and the coordination mode of the ligands. The complete FTIR spectra of the free ligands and their associated Zn (II) complexes are displayed in Supplementary Figures S9–S12, while Figure 2 below presents the spectra across a selected frequency range of 1000–1800 cm^–1^, and Table 1 summarizes the data. The FT-IR spectra of ligands **ImL1** and **ImL2** shown in Figures S9 and S10 displayed absorption bands in the range from 2851 to 3125 cm^−1^, ascribed to C-H stretching vibrations of aromatic rings and alkyl groups, but these bands in the spectra of the Zn (II) complexes in Figure S11 weakened compared to their corresponding ligand. In addition, weak absorption peaks at 1705 and 1701 cm^−1^ were observed in the FTIR spectra of the free ligand in Figure 2a,b. However, these bands disappeared upon coordination of the ligands with the Zn (II) ion. Furthermore, ligand spectra show bands at wavenumbers 1612, 1589, and 1520 cm^−1^ for **ImL1** and 1624, 1597, and 1520 cm^−1^ for **ImL2**, assigned to C=N and C=C functional groups. These bands at 1612 and 1624 cm^−1^, however, completely disappeared in their respective complex spectra, while other bands were shifted to frequencies of 1588 and 1524 cm^−1^ for **ZnCl_2_**(**ImL1**)**_2_** and 1593 and 1524 cm^−1^ for **ZnCl_2_**(**ImL2**)**_2_**, respectively. Additionally, the spectra of both ligands show a band at 1188 cm^−1^ that is completely absent in the spectrum of the Zn (II) complex, further signifying the successful coordination of the ligands with the zinc metal ion.

### 2.4. Structural Characterization by Single Crystal X-ray Diffraction of the Free Ligands ImL1 and ImL2 as Well as and the Corresponding Zn (II) Complex ZnCl_2_(ImL1)_2_

#### 2.4.1. Description of the X-ray Crystal Structures the Free Ligands ImL1 and ImL2

The ligand **ImL1** crystals suitable for X-ray crystallography were prepared by reheating the ligand **ImL1** precipitates in a 50:50 mixture of THF and ethanol until the precipitate dissolved, followed by cooling and slowly evaporating the solvent for several days, yielding colourless crystals of **ImL1**. On the contrary, colourless **ImL2** crystals were produced after heating the **ImL2** precipitate in acetone and progressively evaporating the solvent for several days. The crystal structures for the free ligands **ImL1** and **ImL2** were solved by single crystal X-ray diffraction, and a summary of crystallographic data is presented in Table 2. For a complete collection of single-crystal X-ray diffraction data for **ImL1** and **ImL2**, see the Supplementary Materials from Tables S1–S10.

**Figure 3:**
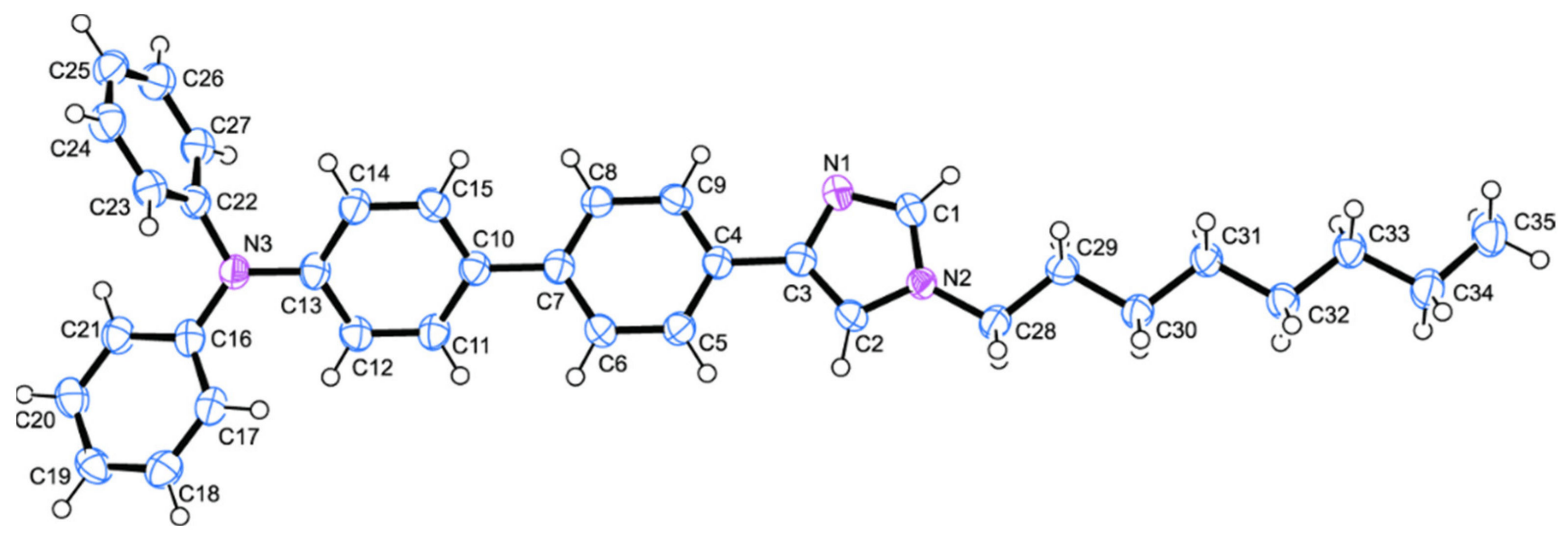
ORTEP diagrams of ligand **ImL1** showing the atom-numbering scheme. Displacement ellipsoids are drawn at the 50% probability level and hydrogen (H) atoms are shown as small spheres of arbitrary radii.

**Figure 4:**
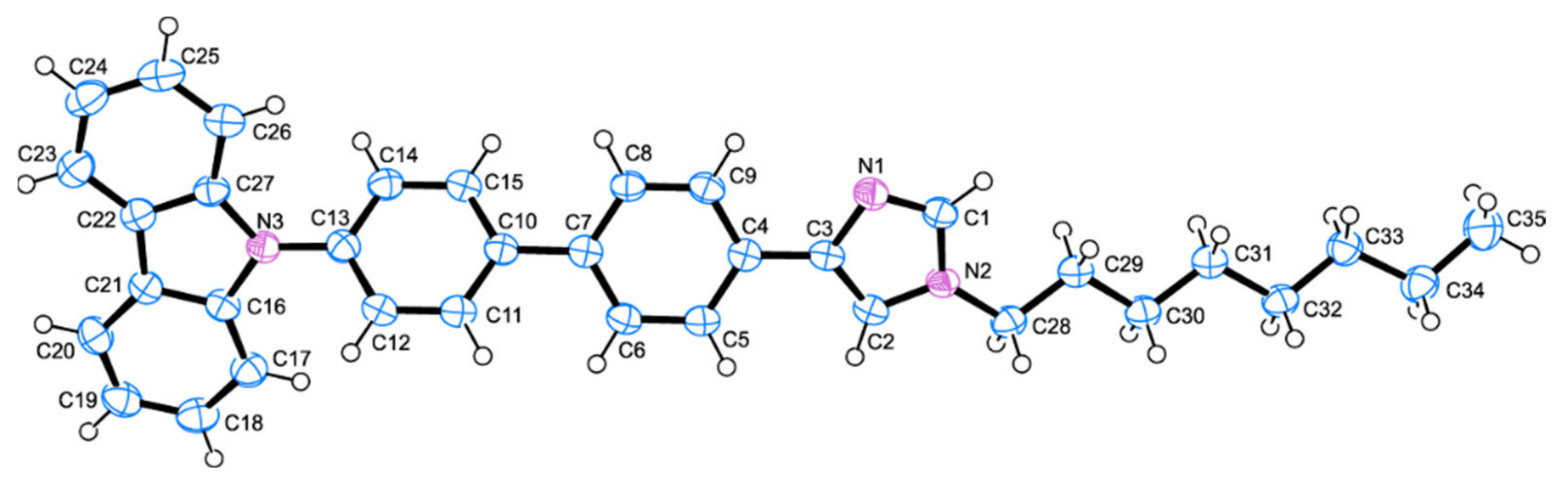
ORTEP Diagrams of ligand **ImL2** showing the atom-numbering scheme. Displacement ellipsoids are drawn at the 50% probability level and hydrogen (H) atoms are shown as small spheres of arbitrary radii.

Ligand **ImL2** crystallized as triclinic with space group P-1, while the ligand **ImL2** crystallized as monoclinic with space group P 21/c. Figure 3 and Figure 4 depict the general arrangement and labeling of the atoms in the free ligands **ImL1** and **ImL2**, respectively, while Table 3 lists the chosen bond lengths and angles. **ImL2**’s three C-N bond lengths range from 1.318 to 1.368 Å, whereas **ImL1**’s three C-N bond lengths range from 1.338 to 1.384 Å, which is comparable to previously reported imidazole derivatives [38,39]. Furthermore, the C-N bond lengths linking an alkyl group and the nitrogen (N2) atom of the imidazole moiety are 1.384 Å for **ImL1** and 1.386Å for **ImL2**, while the C-N bond length connecting three aromatic carbon atoms to the nitrogen (N3) atom was found to be about 1.4 Å for both ligands. The N-C-N angles in the imidazole moiety are found to be 112.6(3)° and 112.44(15)° for **ImL1** and **ImL2**, respectively. Moreover, the C-N-C bond angles of the imidazole motif are 104.3(3)°, 106.9(3)°, 127.0(3)°, and 126.1(3)° for **ImL1** and 105.19(14)° for C(1)-N(1)-C(3), 106.25(14)°, 127.62(14)°, and 126.09(14)° for **ImL2**. Furthermore, bond angles of 120.9(3)° and 129.3(3)° for N(1)-C(3)-C(4) and C(2)-C(3)-C(4) belong to the aromatic systems directly attached to the imidazole units for **ImL1**, while for **ImL2,** they are 121.39(15)° and 129.40(15)° for N(1)-C(3)-C(4) and C(2)-C(3)-C(4). The C(16)-N(3)-C(13) bond angle around the nitrogen atom (N3) directly attached to the carbon (16) atom is found to be 124.7(3)° for **ImL1** and 124.71(14) for **ImL2**. In addition, bond angles of 116.6(3)° for C(16)-N(3)-C(22) and 118.5(3)° for C(13)-N(3)-C(22) were described in **ImL1**, whereas **ImL2** contained bond angles of 108.04(13) for C(16)-N(3)-C(27) and 127.25(14)° for C(13)-N(3)-C(27). This discrepancy in bond angles C(16)-N(3)-C(22) and C(13)-N(3)-C(27) could be attributed to the fact that two aromatic rings in the carbazole moiety in **ImL2** adopt a planar shape, while the structure of the two aromatic rings in **ImL1** resembles propeller blades.

**Figure 5:**
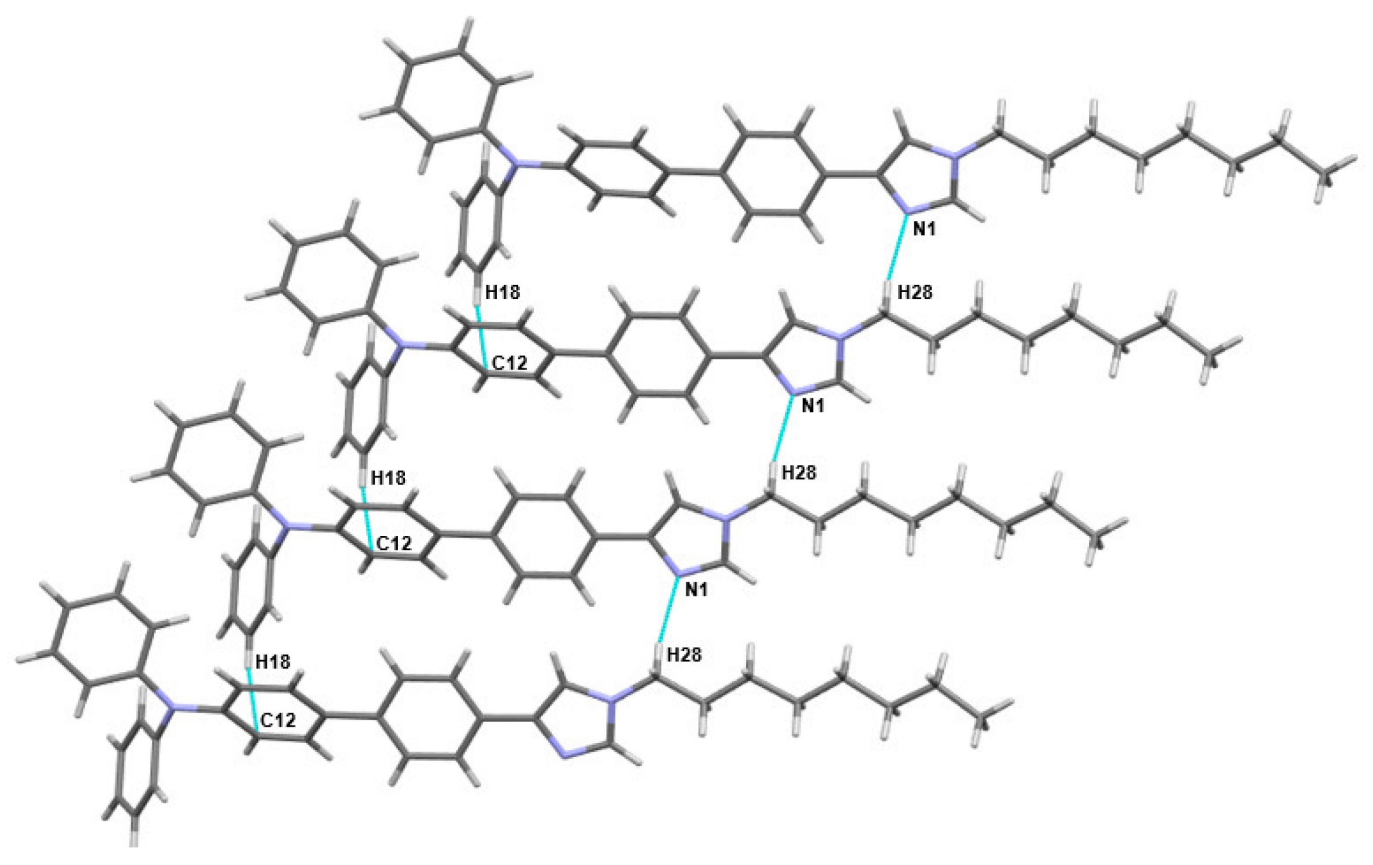
Part of the crystal structure of ligand **ImL1**, showing the intermolecular hydrogen bonds chain parallel to the c-axis and the N(1)----H(28) and H(18)-----C(12) hydrogen bonds (light blue dashed line) linking the adjacent units of this chain.

The **ImL1** ligand’s crystal packing is stabilized by two-hydrogen bonding (Figure 5), resulting in the development of a two-dimensional chain parallel to the crystallographic c-axis. The first intermolecular hydrogen bonding occurs between the imidazole rings’ nitrogen (N1) atom and one of the hydrogen atoms linked to the carbon (C28) atom of the methylene moiety, which is directly attached to the nitrogen (N2) atom of the imidazole rings. A second intermolecular hydrogen bonding occurs between the hydrogen (H18) atom bound to the carbon (C18) atom of the aromatic ring, similar to propeller shafts, and the carbon (C12) atom of the aromatic ring sandwiched between the nitrogen (N3) atom and the aromatic ring carbon bonded directly to the imidazole unit.

**Figure 6:**
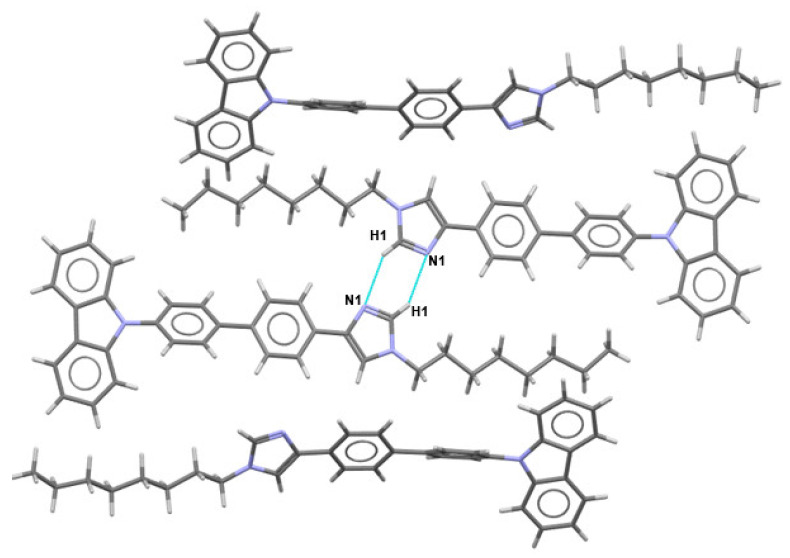
Part of the crystal structure of ligand **ImL2**, showing the polymeric chain parallel to the c-axis and the N(1)----H(1) hydrogen bonds (light blue dashed line) linking the adjacent units of this chain.

The crystal packing of the **ImL2** ligand, on the other hand, is also stabilized by two-hydrogen bonding (Figure 6), but the intermolecular hydrogen bonding occurs between the nitrogen (N1) atom of the imidazole rings and the hydrogen (H1) atom attached to the carbon (C1) atom of the neighbouring imidazole units. The difference in intramolecular interaction between **ImL1** and **ImL2** could be explained by the fact that the *N*-phenylcarbazole motif has a carbon-carbon linkage that forces two phenyl rings into planarity, whereas the two-phenyl rings of the triphenylamine unit have a propeller-like structure. This was reflected in the different bond angles of the *N*-phenylcarbazole motif [C16-N3-C27 with 108.04(13)° and C13-N3-C27 with 127.25(14)°] in comparison to the bond angles of the triphenylamine unit [116.6(3)° for C16-N3-C22 and 118.5 (3)° for C13-N3-C22] (see Table 3).

#### 2.4.2. Description of the X-ray Crystal Structure of Crystal ZnCl_2_(ImL1)_2_ Complex

**Figure 7:**
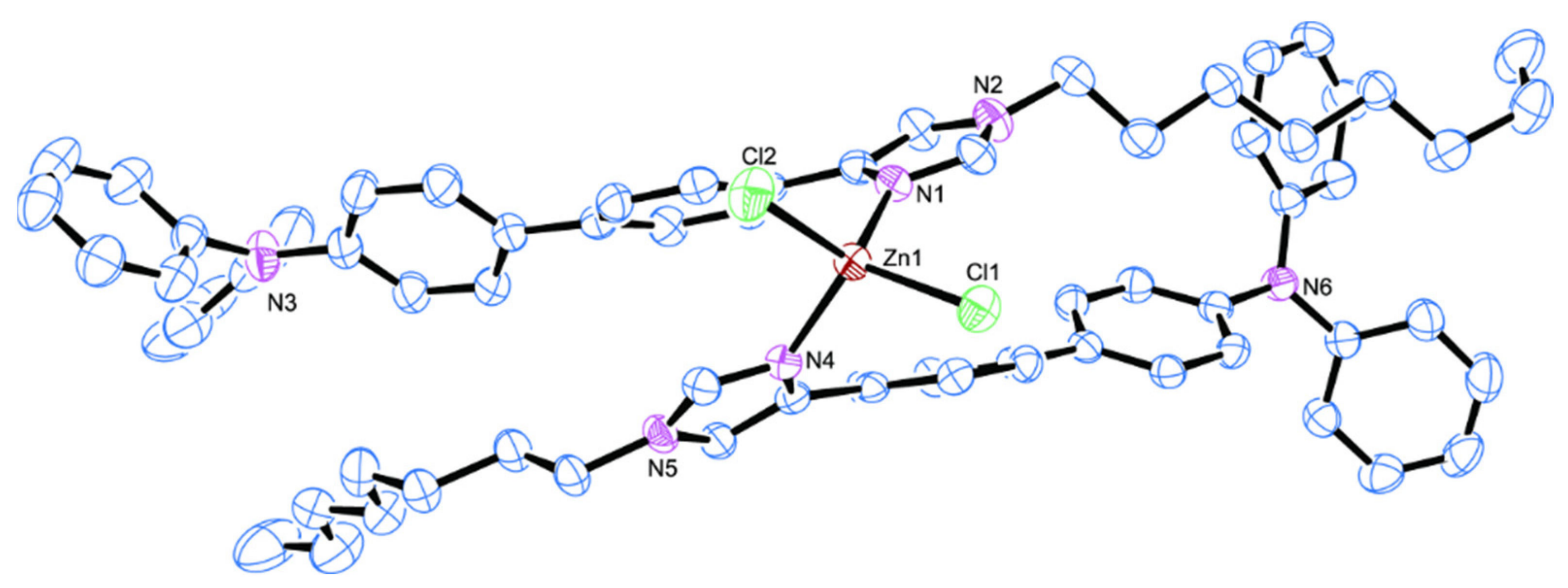
ORTEP Diagram of zinc (II) complex **ZnCl_2_**(**ImL1**)**_2_** showing the atom-numbering scheme. Displacement ellipsoids are drawn at the 50% probability level and H atoms are shown as small spheres of arbitrary radii. H atoms on C atoms are omitted for clarity. Atom labels on C atoms shown by spheres of arbitrary radii.

The **ZnCl_2_**(**ImL1**)**_2_** crystals for X-ray examination were grown in the same way as Ligand **L1** and obtained as pale yellow crystals. The crystallographic data and refinement information for the **ZnCl_2_**(**ImL1**)**_2_** complex are given in Table 2. The overall view and selected labelling of the atoms for clarity in the **ZnCl_2_**(**ImL1**)**_2_** complex are displayed in Figure 7, whereas the designated bond lengths and angles are tabulated in Table 4. A complete collection of single-crystal X-ray diffraction data for the **ZnCl_2_**(**ImL1**)**_2_** complex is given in the Supplementary Materials from Tables S11–S15.

The single X-ray crystal structure of the **ZnCl_2_L1_2_** complex revealed that the complex crystallized as a monoclinic with space group C2. The **ZnCl_2_**(**ImL1**)**_2_** complex has a distorted tetrahedral environment in the zinc (II) ion center, with two coordination sites occupied by the bidentate chelation of two **ImL1** ligands through the nitrogen atom of each ligand and the remaining two coordination sites occupied by two chlorine atoms. The Zn-N bond lengths between the nitrogen atoms of two ligands and the zinc ion were presented to be at distances of 2.029(2) Å and 2.030(2) Å, which are characteristic of Zn(II) complexes with neutral N donors, while the Zn-Cl length was exhibited to be at 2.2274(8) Å and 2.2453(8) Å, respectively. In addition, the N-Zn-N angles around Zn were found to be around the tetrahedral standard of 104.43(9)°, while the Cl-Zn-Cl angle of 110.74(3)° is also centered on the tetrahedral standard, which confirmed that the Zn center forms a tetrahedral coordination geometry. The N-Zn-Cl angles are shown to be 107,24(7)° for N(1)-Zn(1)-Cl(1), 113.76(7)° for N(4)-Zn(1)-Cl(1)°, 114.91(7)° for N(1)-Zn(1)-Cl(2), and 105.77(7)° for N(4)-Zn(1)-Cl(2), respectively. Furthermore, the N-C-N bond angles for the two imidazole moieties of the complex were at 116.6(3)° for N(1)-C(1)-N(2) and 111.6(2)° for N(4)-C(36)-N(5), while the C-N-C bond angles of the same systems ranged from 1056(3)° to 126.5(3)°. The N-Zn-N and Cl-Zn-Cl bond lengths fall within the range of bond lengths in complexes of tetrahedral coordinated Zn with two N-coordinated ligands and two halide atoms previously reported [39,40,41,42].

### 2.5. Photophysical Studies

#### UV-Visible Absorption Studies of the Free Ligands ImL1 and ImL2 as and Their Corresponding Zn (II) Complexes ZnCl_2_(ImL1)_2_

**Figure 8:**
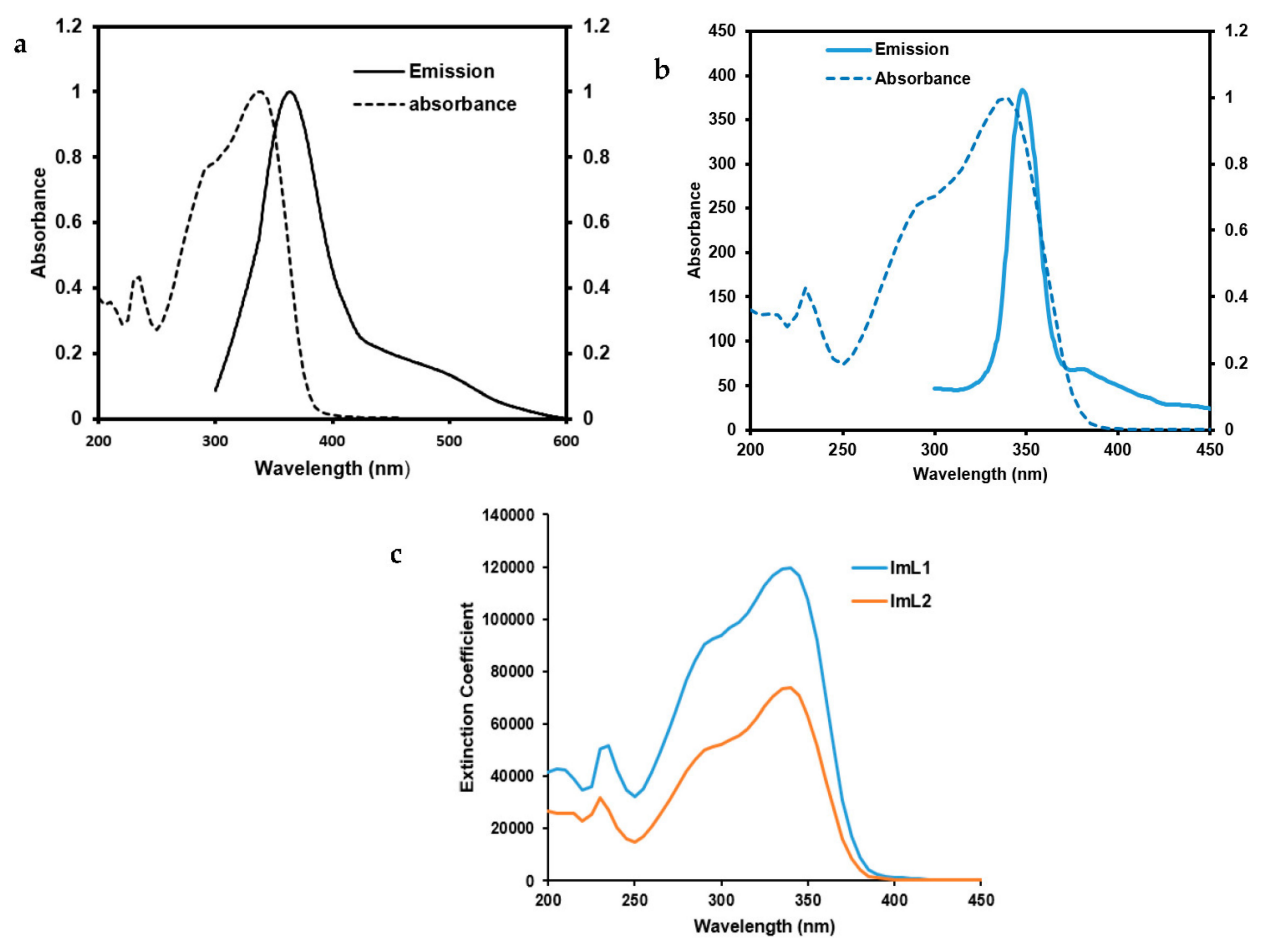
(**a**) Normalized UV−vis absorption (black dotted curve) and emission (black solid curve) spectra of **ImL1** in ethanol. (**b**) UV−vis absorption (blue dotted curve) and emission (blue solid curve) spectra of **ImL2** in ethanol. (**c**) Extinction coefficient of the free ligands **ImL1** (blue) and **ImL2** (orange) in ethanol.

**Figure 9:**
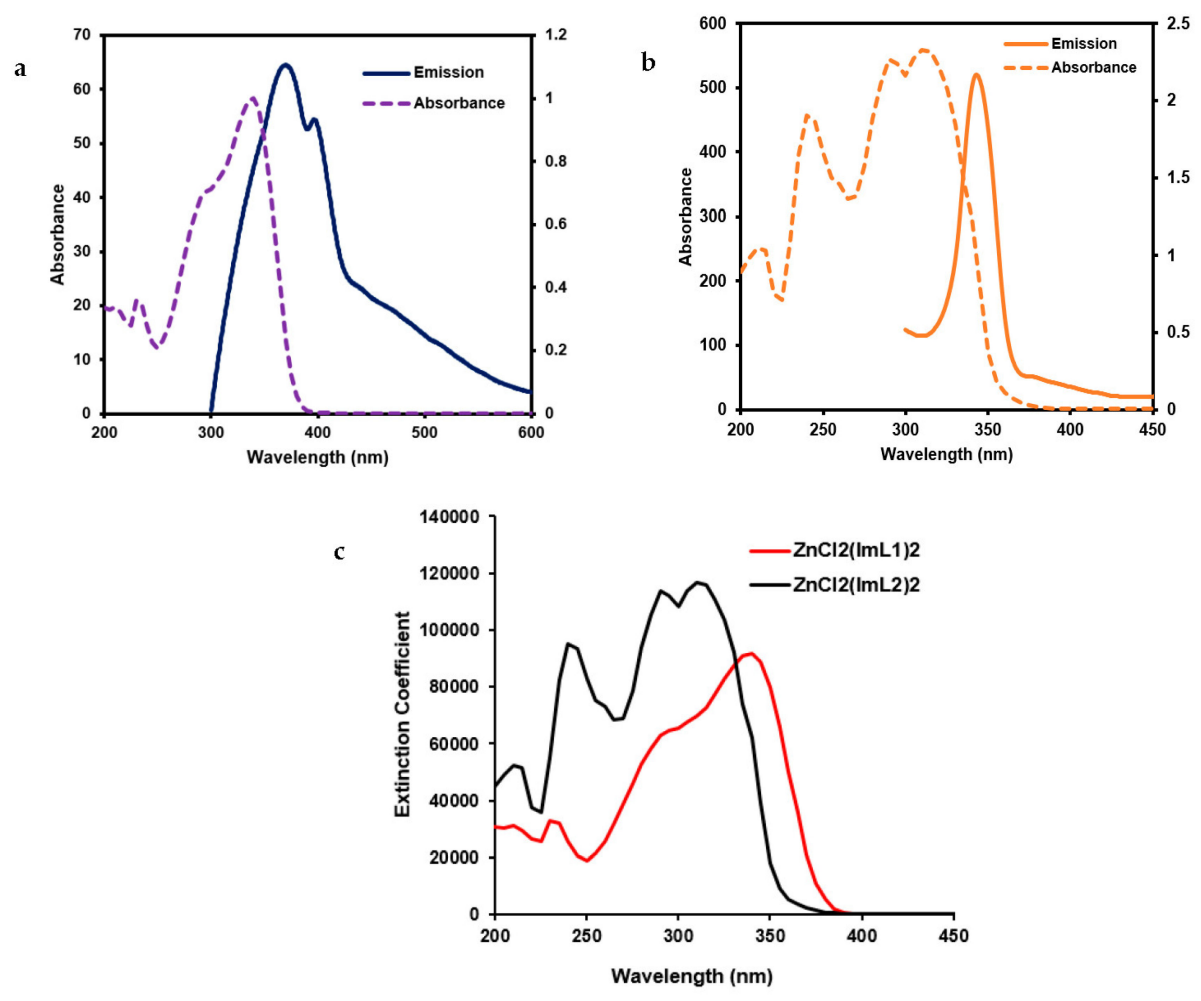
(**a**) Normalized UV−vis absorption (purple dotted curve) and emission (purple solid curve) spectra of **ZnCl_2_**(**ImL1**)**_2_** in ethanol. (**b**) UV−vis absorption (orange dotted curve) and emission (orange solid curve) spectra of **ZnCl_2_**(**ImL2**)**_2_** in ethanol. (**c**) Extinction coefficient of the zinc (II) complexes **ZnCl_2_**(**ImL1**)**_2_** (red) and **ZnCl_2_**(**ImL2**)**_2_** (black).

**Figure 10:**
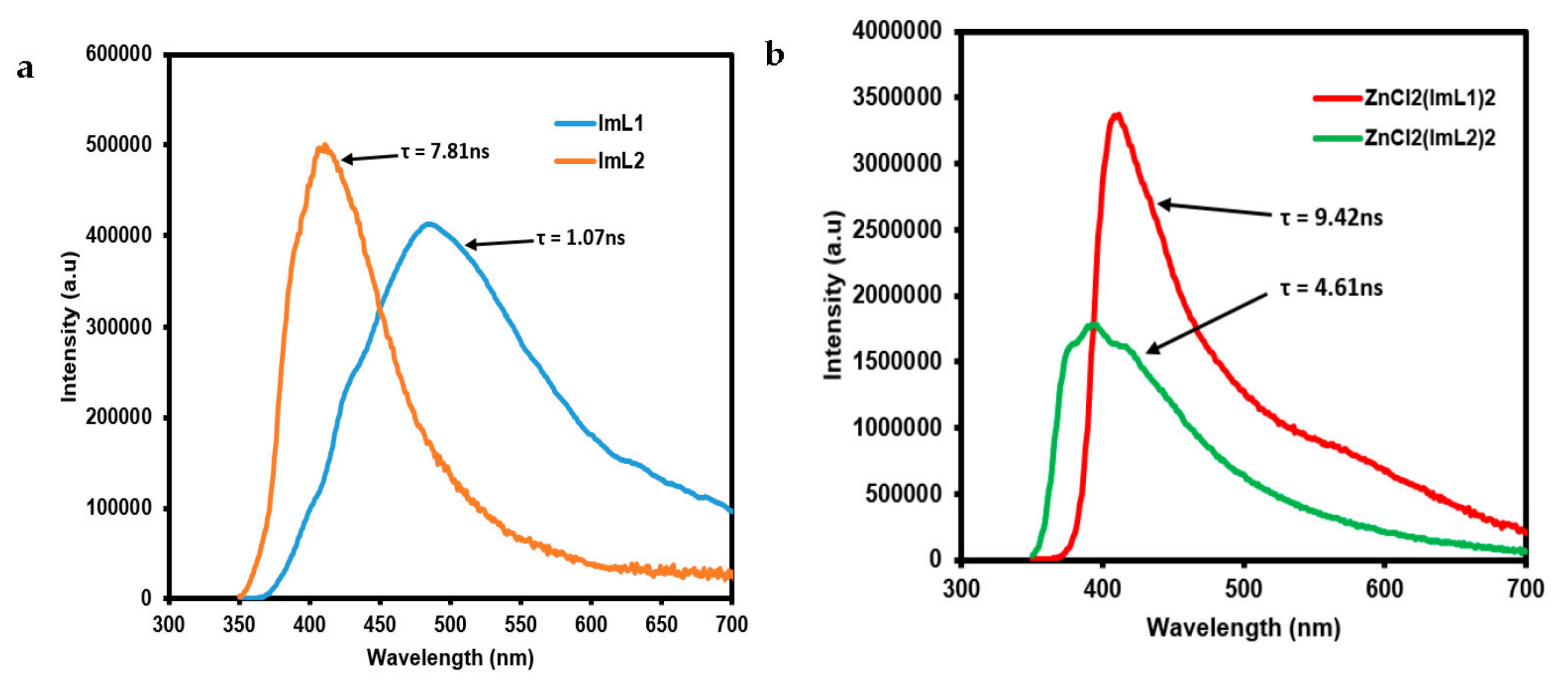
Emission spectra (**a**) of the free ligands (**left**), **ImL1** (Blue) and **ImL2** (orange) as well as (**b**) their zinc (II) complexes (**right**), **ZnCl_2_**(**ImL1**)**_2_** (red) and **ZnCl_2_**(**ImL2**)**_2_** (green) in the solid state at room temperature.

The optical properties of the free ligands and their corresponding complexes were studied by UV-Vis absorption in ethanol solution recorded at room temperature (at a concentration of 2 × 10^−4^ mol/dm^3^) and PL emission spectroscopy in both solid and ethanol solutions. The absorption and emission spectra, including the corresponding extinction coefficients, are shown in Figure 8, Figure 9 and Figure 10, while their numerical data are tabulated in Table 5. The UV-Vis absorption spectra in Figure 8a,b of the free ligands show four bands of similar profile, each with a shoulder peak at 210 nm for **ImL1** (black dotted curve) and 215 nm for **ImL2** (blue dotted curve), which could be attributed to the π–π* transition of the benzene rings, and a second absorption peak at 230 nm for both ligands, which could be attributed to electronic π–π* transfers of the imidazole moieties [43,44,45]. Further, each ligand showed a third shoulder band centering around 295 nm and a fourth main peak at 340, which might be attributed to the usual n–π* transition of the *N*-phenyl-carbazole and triphenylamine moieties [46,47,48,49]. The more intense π–π* transitions of the ligands have extinction coefficients (ε) ranging from 31,660 to 119,415 M^−1^ cm^−1^, with **ImL1** (blue curve in Figure 8c) having the highest value and **ImL2** having the lowest (orange curve in Figure 8c). A high ε results indicate that the compounds absorb light well at the specified wavelength.

Figure 9a,b show the UV-Vis absorption spectra of **ZnCl_2_**(**ImL1**)**_2_** and **ZnCl_2_**(**ImL_2_**)**_2_**, each dissolved in an ethanol solution, which differ greatly from one another. The absorption spectrum of **ZnCl_2_**(**ImL1**)**_2_** displays four bands at 210, 235, 295, and 315 nm (Figure 9a, purple dotted graph) with an extinction coefficient ranging from 31,005 to 91,685 M^−1^cm^−1^ (Figure 9c, black curve), while the **ZnCl_2_**(**ImL2**)**_2_** complex showed five absorption peaks at wavelengths of 210, 240, 260, 290, and 315 nm (Figure 9b, orange dotted graph). The absorption bands at 210, 220, 240, and 290 nm with an extinction coefficient falling in a range of 52,480 to 115,805 M^−1^cm^−1^ (Figure 9c, red curve) correspond to those in the free ligands, which could therefore be assigned to an intraligand (IL) ^1^ π–π* transition, whereas the shoulder at 235 and 335 nm for the **ZnCl_2_**(**ImL1**)**_2_** complex as well as 260 and 315 nm for the **ZnCl_2_**(**ImL2**)**_2_** complex could be assigned to a metal perturbed ligand π–π* transition. The results demonstrate that the complex, **ZnCl_2_**(**ImL2**)**_2_**, absorbs more and reflects less than **ImL1** and **ImL2**, as well as **ZnCl_2_**(**ImL1**)**_2_**.

The study of solid-state luminescence is intriguing because of the potential use of luminescent materials in the fabrication of transparent optoelectronic materials [50,51]. The solid-state photoluminescence of the free ligands and their corresponding Zn (II) complexes at room temperature were investigated, and their data are presented in Figure 10, and the photophysical data are summarized in Table 5. When the samples were excited with a photon source of 300 nm wavelength, emissions ranging from 350 to 800 nm were recorded in the solid state. It is worth mentioning that the intensity of the recorded emission was attenuated by a factor of 100 to prevent detector saturation. The free ligand **ImL1** (Figure 10a, blue curve) displays structured cyan emission with wavelength maxima centered at 488 nm, while the emission spectrum of **ImL2** (Figure 10a, orange curve) is intensely structured in the violet region with wavelength maxima centered at 411 nm.

The luminescence lifetimes of the free ligands in the solid state at room temperature were also measured. The measured luminescence time profiles of the free ligands and their corresponding Zn (II) complexes were well matched with one exponential decay component, and the resulting decay lifetimes (τ) are presented in Table 5 and Supplementary Figures S13–S16. The lifetimes of **ImL1** and **ImL2** were measured to be 1.07 and 7.81 nanoseconds, respectively.

It was observed that the related Zn (II) complexes in the solid state exhibit violet fluorescence emission characteristics, with **ZnCl_2_**(**ImL1**)**_2_** (Figure 10b, red curve) emitting at 410 nm with a fluorescence decay lifetime of 9.42 nanoseconds, whereas **ZnCl_2_**(**ImL2**)**_2_** (Figure 10b, green curve) emitting at 395 nm with a fluorescence decay lifetime of 4.61 nanoseconds. The luminescence in the violet region could be attributed to π–π* intraligand charge transfer (ILCT) transition. Additionally, the fluorescence intensity values of Zn (II) complexes were observed to be much larger than those of their corresponding free ligands, which is consistent with the complexes’ homoleptic nature.

However, when excited at 340 nm in ethanol solution at room temperature, both the free ligands and their corresponding zinc (II) complexes exhibited UV-A emission, with wavelength maxima occurring at 366 nm for **ImL1** (Figure 8a, black solid curve), 350 nm for **ImL2** (Figure 8b, blue solid curve), 372 nm for **ZnCl_2_**(**ImL1**)**_2_** (Figure 9b, purple solid curve), and 345 nm for **ZnCl_2_**(**ImL2**)**_2_** (Figure 9b, orange solid curve). Furthermore, it was observed that the maximum wavelength emission of the **ZnCl_2_**(**ImL1**)**_2_** complex at 372 nm (Figure 9b, violet solid curve) was accompanied by an additional shoulder emission band in a violet region at 399 nm. The free ligands exhibited a small Stokes shift of 26 nm and 10 nm for **ImL1** and **ImL2**, respectively. It is worth mentioning that the complexes hypsochromic emission shift had little effect on the Stokes shift, as demonstrated by stroke shifts of 32 nm for **ZnCl_2_**(**ImL1**)**_2_** and 10 nm for **ZnCl_2_**(**ImL2**)**_2_**, which were equivalent to those of the free ligands.

**Figure 11:**
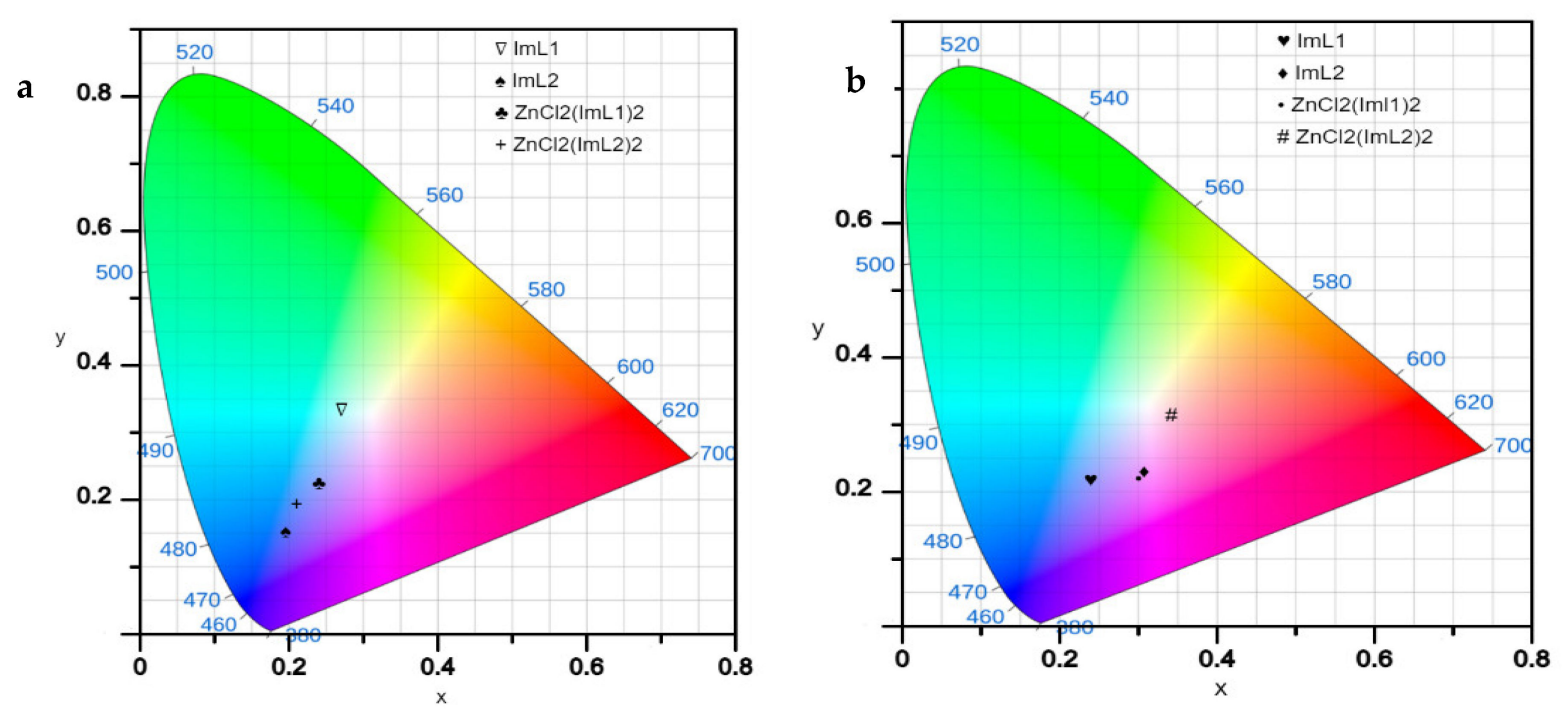
(**a**) CIE chromaticity plots of the free ligands and their corresponding Zn (II) complexes in solid state. (**b**) CIE chromaticity plots of the free ligands and their corresponding Zn (II) complexes in solution state.

Figure 11a shows the Commission Internationale de l’Éclairage (CIE) 1931 colour space of the PL emission in the solid state of the free ligands and the corresponding Zn (II) complexes. The **ImL1** ligand emits cyan light with CIE x and y coordinates of 0.27 and 0.333, while the **ImL2** ligand exhibits violet light with CIE coordinates of 0.196 and 0.152 in solid form. Both Zn (II) complexes produce violet light colours, with CIE coordinates of (0.239, 0.224) for **ZnCl_2_**(**ImL1**)**_2_** and (0.21, 0.193) for **ZnCl_2_**(**ImL2**)**_2_**, respectively. In the solution phase, however, the colours of both free ligands and their corresponding Zinc (II) complexes change to violet, with CIE coordinates of (0.239, 0.216) for **ImL1**, (0.308, 0.23) for **ImL2**, (0.303, 0.219) for **ZnCl_2_**(**ImL1**)**_2_**, and (0.342, 0.313) for **ZnCl_2_**(**ImL1**)**_2_** (Figure 11b).

### 2.6. Thermal Stability Studies

**Figure 12:**
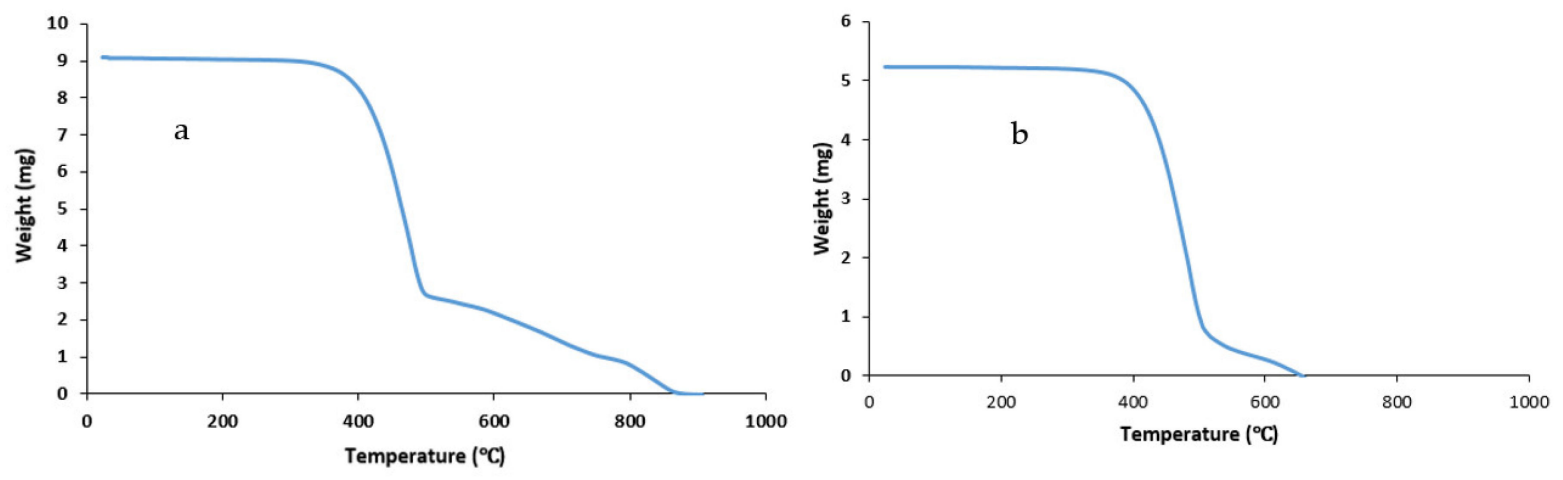
Thermogravimetric analysis (TGA) of the zinc (II) complexes, (**a**) **ZnCl_2_**(**ImL1**)**_2_** and (**b**) **ZnCl_2_**(**ImL2**)**_2_**.

The thermal stability of the zinc (II) complexes **ZnCl_2_**(**ImL1**)**_2_** and **ZnCl_2_**(**ImL_2_**)**_2_** was investigated using thermogravimetric analysis (TGA). TGA tests were carried out in an inert environment at a heating rate of 10 °C per minute throughout a temperature range of 23–900 °C. The extraordinary heat stability of the zinc (II) complex is overcome by the beginning of complex disintegration (Td), which corresponds to a 2% weight loss. The TGA profile for the **ZnCl_2_**(**ImL1**)**_2_** complex in Figure 12a shows a two-step profile: the first decomposition phase takes place at 341 °C, losing 2% of the composite’s weight, while the second step occurs at 500 °C, demonstrating the loss of the entire organic moiety with 32.5% of the complex’s total weight. Despite having a similar TGA profile to the **ZnCl_2_**(**ImL1**)**_2_**, the **ZnCl_2_**(**ImL2**)**_2_** complex in Figure 12b had a 2% weight decomposition at 365 °C, followed by a 32.5% weight loss at 514 °C due to the disintegration of entire organic units. A convoluted pattern of weight losses began during the second stage of the TGA profiles for the complexes, and it lasted until all was lost at 656 °C for **ZnCl_2_**(**ImL2**)**_2_** and 861 °C for **ZnCl_2_**(**ImL1**)**_2_**.

## 3. Materials and Methods

### 3.1. General Methods

All chemical reagents were bought from Merck and used without additional purification. All dried solvents were used directly from an LC-Tech SP-1 argon gas-stored solvent purifier. Unless otherwise stated, all reactions were monitored by analytical thin layer chromatography (TLC) using precoated Merck silica gel F254 plates and visualized under UV light (254 nm). Unless otherwise stated, the products were separated by column chromatography using Merck Silica Gel 60 [particle size 0.040–0.063 mm (230–400 mesh)] using various ratio of ethyl acetate to hexane. FTIR data were acquired using an Attenuated Total Reflectance (ATR) Fourier Transform Infrared spectroscope (Thermo Nicolet 5700 FTIR). NMR data were obtained at 298 K with a 400 MHz Bruker Avance spectrometer equipped with a BBI 5 mm probe. The solvent peaks for proton NMR of 7.26 (CDCl_3_) and 2.50 (DMSO-*d*_6_) and 77.0 (CDCl_3_) and 39.52 (DMSO-*d*_6_) for carbon-13 NMR were used as internal reference, chemical shifts are expressed in parts per million (ppm). Single X-ray crystallography data on intensity were collected using an Oxford Cryostream 600 cooler on a Bruker D8 Venture Microfocus with Photon III CCD area detector diffractometer with graphite-monochromator MoKα_1_ (λ = 0.71073 Å) radiation at 173 K. SAINT+, version 6.02 [52], was used to reduce the data, and SADABS [50] was used to make empirical absorption corrections. XPREP [52] was used to create space group assignments. The structure was solved using SHELXT [51] and refined using full-matrix least-squares or difference Fourier techniques on F2 using SHELXL-2017 in the WinGX [53] Suite of tools [54]. All C-bound hydrogen atoms were idealized and refined as riding atoms with isotropic characteristics that were 1.2 or 1.5 times those of their parent atoms. ORTEP-3 [54] and PLATON [55] were used to create diagrams and publication content. The absorbance properties of the free ligands and their reacting complexes were measured employing a Thermo Logical Multiskan GO UV/Vis spectrophotometer. Information was collected on tests kept in 1-cm plastic cuvettes with ethanol as the reference solvent. The molar extinction coefficient was calculated using the formula in Equation (1):

A = εbc(1)

where A is the absorbance, ε is the molar extinction coefficient, b is the path length of the incident photon and c is the concentration of the material in solution. Photoluminescence was measured in quartz cuvettes (1 cm path length) employing a xenon light (150 W) and ethanol as the reference solvent, utilizing Perkin Elmer LS photoluminescence spectroscopy. Steady-state PL spectra were acquired at room temperature using a Horiba QM8000 spectrofluorometer with a Xenon lamp. An excitation wavelength of 300 nm was used for all four powder samples. The luminescence was attenuated by a Neutral Density filter with Optical Density 2 (ND2) in order to prevent saturation of the detector due to the intensity of the luminescence. The lifetimes of the samples were measured using the TCSPC mode of the same instrument and a pulsed diode laser at 377 nm as the excitation source. The CIE 1931 (x, y) chromaticity diagram was plotted using an online interactive CIE 1931 chromaticity diagram application developed by the department of physics at the University of the Free State [56]. Thermogravimetric investigation (TGA) was performed on a Perkin Elmer Pyris 6 TGA from 23 °C up to 900 °C beneath a nitrogen environment (stream rate 20 mL/min) with a warming rate of 10 °C/min.

Crystallographic data reported in this article has been deposited with the Cambridge Crystallographic Data Center, under deposition codes 2,270,110 for **ImL1**, 2,270,107 for **ImL2**, and 2,270,111 for **ZnCl_2_**(**ImL1**)**_2_**.

### 3.2. Synthesis of the Free Ligands

#### 3.2.1. Synthesis of 4-(4-Bromophenyl)-1-Octyl-1*H*-Imidazole 3

In a two-necked round-bottom flask containing a mixture of 4-(4-bromophenyl)-1*H*-imidazole **1** (2.15 g, 9.17 mmol), 1-bromooctane **2** (3.4 mL, 19.5 mmol), NaOH (50% in H_2_O, 10 mL, 0.54 mol), and benzyltetraethylammonium chloride (1.43 g, 6.28 mmol), was added toluene (30 mL). After 24 h of heating at 110 °C, the mixture was cooled to room temperature. It was then poured into water and extracted with ethyl acetate after cooling. The organic extracts were dried with anhydrous MgSO_4_ after being rinsed with a brine solution. The crude product was separated by silica gel chromatography (eluting with 1% ethyl acetate in a hexane ratio) to give 4-(4-bromophenyl)-1-octyl-1*H*-imidazole 3 as a white solid (2.77 g, 90%). ^1^H NMR (400 MHz, CDCl_3_) δ ppm: 7.62 (2H, q, *J* = 8.4 Hz, ArH), 7.45–7.47 (3H, m, ArH), 7.18 (1H, s, ArH), 3.89 (2H, t, *J* = 7.0 Hz, CH_2_), 1.79 (2H, m, Hz, CH_2_), 1.29–1.31 (10H, m, 5 × CH_2_), 0.85 (3H, t, *J* = 6.4 Hz, CH_3_) ppm. ^13^C NMR (125 MHz, CDCl_3_) δ ppm: 141.0, 137.3, 133.3, 131.5, 126.2, 120.1, 114.8 (ArC), 47.2, 31.6, 30.9, 29.0, 28.9, 26.5, 22.5 (7 × CH_2_), 14.0 (CH_3_).

#### 3.2.2. Synthesis of 4′-(1-Octyl-1*H*-Imidazol-4-yl)-*N*,*N*-Diphenyl-[1,1′-Biphenyl]-4-yl)-4-Amine ImL1

A solution of 4-(4-bromophenyl)-1-octyl-1*H*-imidazole **3** (1.32 g; 3.94 mmol), 4-(diphenylamino)phenylboronic acid pinacol ester **4** (1.53 g; 4.14 mmol), XPhos Pd G3 (202.29 mg; 0.16 mmol), and potassium carbonate (5.84 g; 17.92 mmol) in anhydrous 1,4-dioxane (60 mL) was sonicated under argon for 5 min. To this was added water (1 mL), and the reaction mixture was heated to 100 °C for 20 h. The cooled reaction mixture was diluted with water (250 mL), acidified with HCl (2 M), and extracted with ethyl acetate (4 × 100 mL). The organic layer was filtered through celite, dried over MgSO_4_, filtered, and concentrated under reduced pressure to afford brown solids. This solid was purified by column chromatography over silica (eluent: 20–100% (1:9 EtOAc: Hexane)), and clean fractions were combined and concentrated under reduced pressure to give 4′-(1-octyl-1*H*-imidazol-4-yl)-*N*,*N*-diphenyl-[1,1′-biphenyl]-4-yl)-4-amine **ImL1** as a yellow solid (1.3 g, 49.6% yield). ^1^H NMR (400 MHz, CDCl_3_) δ ppm: 7.79 (2H, d, *J* = 8.4 Hz, ArH), 7.56 (2H, *J* = 8.4 Hz, ArH), 7.49–7.50 (3H, m, ArH), 7.20–7.26 (5H, m, ArH), 7.10–7.12 (6H, m, ArH), 6.98 (2H, t, *J* = 7.4 Hz, 2H), 3.90 (2H, t, *J* = 7.2 Hz, CH_2_), 1.78 (2H, t, *J* = 6.8 Hz, CH_2_), 1.22–1.30 (10H, m, 5 × CH_2_), 0.84 (3H, t, *J* = 6.8 Hz, CH_3_). ^13^C NMR (101 MHz, CDCl_3_) δ ppm: 148.1, 147.3, 143.0, 137.5, 135.8, 132.5, 129.7, 129.3, 127.7, 127.3, 124.7, 124.6, 123.3, 123.2, 122.5, 118.7, 118.6 (ArC), 50.3, 35.3, 31.6, 30.1, 29.0, 26.3, 22.5 (7 × CH_2_), 14.0(CH_3_). FTIR *ν*_max_/cm^−1^: 3121, 3032, 2920, 2851, 1705, 1589, 1520, 1481, 1466, 1327, 1285, 1281, 1188, 1103, 1069, 941, 818, 752, 694, 617, 517, 447.

#### 3.2.3. Synthesis of 9-(4′-(1-Octyl-1*H*-Imidazol-4-yl)-[1,1′-Biphenyl]-4-yl)-9*H*-Carbazole ImL2

A solution of 4-(4-bromophenyl)-1-octyl-1*H*-imidazole **3** (3.00 g; 5.97 mmol), 9*H*-Carbazole-9-(4-phenyl) boronic acid pinacol ester **5** (1.76 g; 5.97 mmol), XPhos Pd G3 (202.29 mg; 0.24 mmol), and potassium carbonate (5.84 g; 17.92 mmol) in anhydrous 1,4-dioxane (60.00 mL) were sonicated under argon for 5 min. To this was added water (1.00 mL), and the reaction mixture was heated to 100 °C for 20 h. The cooled reaction mixture was diluted with water (250 mL), acidified with HCl (2 M), and extracted with ethyl acetate (4 × 100 mL). The organic layer was filtered through celite, dried over MgSO_4_, filtered, and concentrated under reduced pressure to afford brown solids. This solid was purified by column chromatography over silica (eluent: 20–100% (1:9 EtOAc: Hexane)), and clean fractions were combined and concentrated under reduced pressure to give-(4’-(1-octyl-1H-imidazol-4-yl)-[1,1’-biphenyl]-4-yl)-9*H*-carbazole **ImL2 as a** pale yellow solid (1.3 g, 49.6% yield). ^1^H NMR (400 MHz, CDCl_3_) δ ppm: 7.18 & 7.97 (2H, 2 × s, ArH), 7.87 91H, d, *J* = 8.4 Hz, ArH), 7.63–7.65 (2H, m, ArH), 7.52–7.57 (2H, m, ArH), 7.44–7.49 (7H, m, ArH), 7.30–7.49 (2H, m, ArH), 7.19 (1H, d, *J* = 3.2 Hz, ArH), 6.61 (1H, d, *J* = 2.4 Hz, ArH), 4.16 (2H, t, *J* = 7.2 Hz, CH_2_), 1.88 (2H, t, *J* = 6.4 Hz, CH_2_), 1.29–1.37 (10H, m, 5 × CH_2_), 0.89 (3H, t, *J* = 6.4 Hz, CH_3_). ^13^C NMR (101 MHz, CDCl_3_) δ ppm: 141.8, 141.0, 135.8, 135.7, 131.7, 129.1, 128.8, 128.6, 127.2, 125.9, 123.4, 121.1, 120.2, 119.9, 119.6, 110.0, 109.9, 101.4 (ArC), 46.6, 31.8, 30.4, 29.3, 29.1, 27.1, 22.7 (7 × CH_2_), 14.1 (CH_3_). FTIR *ν*_max_/cm^−1^: 3109, 3055, 2940, 2855, 1701, 1597, 1520, 1477, 1450, 1362, 1319, 1227, 1188, 1111, 941, 829, 745, 718, 625, 5559, 517, 520.

### 3.3. Preparation Zinc Complexes

#### 3.3.1. Synthesis of ZnCl_2_(ImL1)_2_ Complex

A mixture of zinc (II) chloride (41 mg, 0.30 mmol) and 4-(1-octyl-1*H*-imidazol-4-yl)-*N*,*N*-diphenyl-[1,1-biphenyl]-4-yl)-4-Amine **ImL1** (300 mg, 0.60 mmol) in methanol (10 mL) was refluxed for 24 h. The reaction mixture was allowed to cool and kept at room temperature for three days. The resulting precipitates were separated by filtration, washed with water (2 × 3 mL), and air dried to obtain the desired novel **ZnCl_2_**(**ImL1**)**_2_** complex as pale yellow solids. Yield: 375 mg, 55% based on ligand **ImL1**. ^1^H NMR (400 MHz, CDCl_3_) δ ppm: 8.13 (2H, s, ArH), 7.30–7.34 (12H, m, overlapping signals, ArH), 7.22–7.24 (12H, m, ArH), 7.02–7.10 (10H, m, ArH), 7.00–7.10 (14H, m, ArH), 6.63 (2H, s, ArH, 3.70 (4H, *t*, *J* = 7.2 Hz, CH_2_), 1.56 (4H, d, *J* = 20 Hz, CH_2_), 1.18–1.25 (20H, m, 5 × CH_2_), 0.80 (6H, t, *J* = 6.8 Hz, CH_3_).^13^C NMR (101 MHz, CDCl_3_) δ ppm: 147.5, 147.4, 140.9, 139.8, 139.6, 133.8, 129.4, 129.2, 127.9, 127.2, 125.5, 123.5, 123.2, 115.8 (ArC), 48.2, 31.7, 30.6, 29.1, 28.9, 26.4, 22.6 (7 × CH_2_), 14.1 (CH_3_). FTIR *ν*_max_/cm^−1^: 3125, 1531, 1516, 1327, 1288, 1088, 934, 826, 934, 826, 768, 660, 621.

#### 3.3.2. Synthesis of ZnCl_2_(ImL2)_2_ Complex

A mixture of zinc (II) chloride (35 mg, 0.256 mmol) and 9-(4’-(1-octyl-1*H*-imidazol-4-yl)-[1,1’-biphenyl]-4-yl)-9*H*-carbazole **ImL2** (255 mg, 0.512 mmol) in methanol (10 mL) was refluxed for 24 h. The reaction mixture was allowed to cool and kept at room temperature for three days. The resulting precipitates were separated by filtration, washed with water (2 × 3 mL), and air dried to obtain the desired novel **ZnCl_2_**(**ImL2**)**_2_** complex as white solids. Yield: 342 mg, 59% based on ligand **ImL2**. ^1^H NMR (400 MHz, CDCl_3_) δ ppm: 8.26 (4H, d, *J* = 8.0 Hz, ArH), 7.91 (4H, d, *J* = 8.8 Hz, ArH), 7.78 (4H, d, *J* = 8.4 Hz, ArH), 7.69–7.77 (8H, m, ArH), 7.45 (4H, d, *J* = 4.0 Hz, ArH), 8.26 (4H, d, *J* = 8.0 Hz, ArH), 7.30–7.33 (12H, m, ArH), 4.00 (4H, *t*, *J* = 7.0 Hz, CH_2_), 1.74 (4H, d, *J* = 6.8 Hz, CH_2_), 1.18–1.25 (20H, m, 5 × CH_2_), 0.83 (6H, t, *J* = 6.8 Hz, CH_3_). ^13^C NMR (101 MHz, CDCl_3_) δ ppm: 140.1, 140.0, 139.0, 138.2, 136.8, 135.9, 134.0, 128.0, 127.0, 126.8, 126.3, 125.0, 122.8, 1206, 116.3, 109.7 (ArC), 46.4, 31.2, 30.4, 28.6, 28.5, 26.0, 22.1 (7 × CH_2_), 14.0 (CH_3_). FTIR *ν*_max_/cm^−1^: 3125, 3044, 2924, 2851, 1593, 1501, 1493, 1450, 1362, 1319, 1227, 1115, 1076, 1003, 818, 745, 721, 637, 563, 536, 478, 420.

## 4. Conclusions

Two new fluorescent ligands, 4-(1-octyl-1*H*-imidazol-4-yl)-*N*,*N*-diphenyl-[1,1-biphenyl]-4-yl)-4-amine **ImL1** and 9-(4 -(1-Octyl-1*H*-imidazol-4-yl)-[1,1-biphenyl]-4-yl)-9*H*-carbazole **ImL2** were synthesized in 74% and 66% yields, respectively. These ligands were later treated with zinc chloride to give their corresponding zinc (II) chloride complexes, **ZnCl_2_**(**ImL1**)**_2_** and **ZnCl_2_**(**ImL2**)**_2_,** in up to 59% yield. FTIR, ^1^H, and ^13^C-NMR confirmed the molecular structures of the free ligands and their corresponding Zn (II) complexes. The crystal structure analysis shows that the **ImL1** ligand and the **ZnCl_2_**(**ImL1**)**_2_** complex crystallized in triclinic with space groups P-1 and C2, respectively, while the **ImL2** ligand crystallized in monoclinic with space group P 21/c. In addition, the **ZnCl_2_**(**ImL1**)**_2_** complex shows a distorted tetrahedron geometry with two coordination sites occupied by the bidentate chelation of two **ImL1** ligands through the nitrogen atom of each ligand and the remaining two coordination sites occupied by two chlorine atoms. Both zinc complexes are thermally stable at 341 °C for **ZnCl_2_**(**ImL1**)**_2_** and 365 °C for **ZnCl_2_**(**ImL2**)**_2_**, corresponding to 2% weight loss. Both the free ligands and their complexes display efficient photoluminescence properties in the violet to cyan spectral range from 410 to 488 nm in the solid state at room temperature, with fluorescence decay lifetimes ranging from 1 to 9 nanoseconds, as well as good photoluminescence properties in the UV-A emission area from 345 to 372 nm in ethanol solutions. Because of these potential photophysical features, additional research is being conducted to assess these products as optoelectronic materials.

## Figures and Tables

**Table 1:** FTIR signals of ligands **ImL1** and **ImL2** as well as their related **ZnCl_2_**(**ImL**)**_2_** complexes.

ImL1	ZnCl_2_(ImL1)_2_	ImL2	ZnCl_2_(ImL2)_2_
3121w ^1^ 3063w 3032w 2920sh ^2^ 2851sh	3117w 3060w 3033w 2931sh 2852sh	3109w 3055w 2924sh 2855sh	3125w 3044w 2920sh 2851w
1717sh 1612sho ^3^ 1589s ^4^ 1520sho 1481sh 1466sho 1327m 1285m 1265m 1188m 1107w 1072w 941sh	1588s 1524w 1487s 1473sho 1328sh 1315sho 1272s 1206sh 1076sh 975sh	1701sh 1624sho 1597sh 1520sh 1516sh 1477s 1450s 1362sho 1335sho 1319sh 1227s 1188sh 1115sh 1069sh 941sh	1593sh 1524sho 1493sh 1450sh 1362sh 1362sh 1319sh 1227s 1245sh 1119w 1080sh 976sh

Abbreviations: ^1^ w = weak, ^2^ sh = sharp, ^3^ sho = shoulder, ^4^ s = strong.

**Table 2:** Crystal data of ligands **ImL1** and **ImL2** as well as zinc (II) complex, **ZnCl_2_(ImL1)_2_**.

	Ligand ImL1	Ligand ImL2	ZnCl_2_(ImL1)_2_ Complex
Empirical Formula	C_35_H_37_N_3_	C_35_H_35_N_3_	C_70_H_74_Cl_2_N_6_Zn
Fw/g/mol	499.67	497.66	1135.62
T/K	173(2)	173(2)	173(2)
Wavelength/Å	0.71073	0.17073	0.17073
Crystal size/mm^3^	0.283 × 0.173 × 0.116	0.343 × 0.105 × 0.038	0.343 × 0.173 × 0.080
Crystal system	Triclinic	Monoclinic	Monoclinic
Space group	P-1	P 21/c	C2
a/Å	6.0762(8)	18.4314(13)	15.126(4)
b/Å	8.7675(12)	9.1307(7)	8.9495(19)
c/Å	25.828(3)	16.3253(10)	44.620(11)
α/°	81.639(5)	90	90
β/°	84.848(6)	101.904(3)	98.154
γ/°	86.516(5)	90	90
V/Å [3]	1354.2(3)	2688.3(3)	5979(2)
Z	2	4	4
Dc/g·cm^−3^	1.225	1.230	1.262
F(000)	536	1064	2400
Theta range for data collection	2.351 to 25.249°	2.259 to 25.500°.	2.306 to 27.999°
Completeness to theta = 25.242°	99.5%	100.0%	99.8%
Refinement method	Full-matrix least square on F^2^	Full-matrix least square on F^2^	Full-matrix least square on F^2^
Data/restraints/parameters	5029/0/344	4999/0/344	14,393/1/714
Reflections collected	22,301	110,205	110,278
Goodness-of-fit on F^2^	1.083	1.077	1.045
Independent reflections	5029 [R(int) = 0.0631]	4999 [R(int) = 0.1232]	14,393 [R(int) = 0.0557]
R1, wR2 (I > 2σ(I))	R_1_ = 0.0809, wR_2_ = 0.2122	R1 = 0.0411, wR2 = 0.0941	R_1_ = 0.0365, wR_2_ = 0.0843
R_1_, wR_2_ (all data)	R_1_ = 0.0992, wR_2_ = 0.2244	R1 = 0.0685, wR2 = 0.1129	R_1_ = 0.0414, wR_2_ = 0.0866
Large diff. and hole/e.Å^−3^	0.296 and −0.272	0.161 and −0.170	0.430 and −0.247

**Table 3:** Selected bond lengths [Å] and angles [°] for ligands **ImL1** and **ImL2**.

Ligand ImL1			
	Bond Lengths [Å]		Bond Angles [°]
C(1)-N(1)	1.328(4)	N(1)-C(1)-N(2)	112.6(3)
C(1)-N(2)	1.337(4)	N(1)-C(3)-C(4)	120.9(3
C(2)-N(2)	1.368(4)	C(2)-C(3)-C(4)	129.3(3)
C(3)-N(1)	1.384(4)	C(1)-N(1)-C(3)	104.3(3)
C(13)-N(3)	1.418(4)	C(1)-N(2)-C(2)	106.9(3)
C(16)-N(3)	1.414(4)	C(1)-N(2)-C(28)	127.0(3)
C(22)-N(3)	1.434(4	C(2)-N(2)-C(28)	126.1(3)
C(28)-N(2)	1.471(4)	C(16)-N(3)-C(13)	124.7(3)
-	-	C(16)-N(3)-C(22)	116.6(3)
-	-	C(13)-N(3)-C(22)	118.5(3)
**Ligand** **ImL2**			
	**Bond lengths [Å]**		**Bond angles [°]**
C(1)-N(1)	1.318(2)	N(1)-C(1)-N(2)	112.44(15)
C(1)-N(2)	1.357(2)	N(1)-C(3)-C(4)	121.39(15)
C(2)-N(2)	1.369(2)	C(2)-C(3)-C(4)	129.40(15)
C(3)-N(1)	1.386(2)	C(1)-N(1)-C(3)	105.19(14)
C(13)-N(3)	1.420(2)	C(1)-N(2)-C(2)	106.25(14)
C(16)-N(3)	1.396(2)	C(1)-N(2)-C(28)	127.62(14)
C(27)-N(3)	1.398(2)	C(2)-N(2)-C(28)	126.09(14)
C(28)-N(2)	1.467(2)	C(16)-N(3)-C(13)	124.71(14)
		C(16)-N(3)-C(27)	108.04(13)
		C(13)-N(3)-C(27)	127.25(14)

**Table 4:** Selected bond lengths [Å] and angles [°] for **ZnCl_2_(ImL1)_2_**.

	Bond Lengths [Å]		Bond Angles [°]
C(1)-N(1)	1.318(4)	N(1)-C(1)-N(2)	111.6(3)
C(1)-N(2)	1.336(4)	N(4)-C(36)-N(5)	111.6(2)
C(2)-N(2)	1.371(4)	C(38)-C(37)-N(5)	106.5(2)
C(3)-N(1)	1.389(4)	C(37)-C(38)-N(4)	108.6(2)
C(36)-N(4)	1.319(3)	C(1)-N(1)-C(3)	105.6(2)
C(36)-N(5)	1.327(4)	C(1)-N(1)-Zn(1)	120.21(19)
C(37)-N(5)	1.377(4	C(3)-N(1)-Zn(1)	132.68(19)
C(38)-N(4)	1.386(3)	C(1)-N(2)-C(2)	107.2(2)
N(1)-Zn(1)	2.029(2)	C(1)-N(2)-C(28)	126.5(3)
N(4)-Zn(1)	2.030(2)	C(2)-N(2)-C(28)	126.2(3)
Zn(1)-Cl(1)	2.2274(8)	C(13)-N(3)-C(16)	122.2(3)
Zn(1)-Cl(2)	2.2453(8)	C(13)-N(3)-C(22)	120.0(3)
-	-	C(16)-N(3)-C(22)	117.1(3)
-	-	C(36)-N(4)-C(38)	105.9(2)
-	-	C(36)-N(4)-Zn(1)	120.34(19)
-	-	C(38)-N(4)-Zn(1)	131.11(18)
-	-	C(36)-N(5)-C(37)	107.4(2)
-	-	C(36)-N(5)-C(63)	126.4(2)
-	-	C(37)-N(5)-C(63)	126.1(2)
-	-	C(51)-N(6)-C(48)	119.1(2)
-	-	C(51)-N(6)-C(57)	121.0(2)
-	-	C(48)-N(6)-C(57)	118.2(2)
-	-	N(1)-Zn(1)-N(4)	104.43(9)
-	-	N(1)-Zn(1)-Cl(1)	107.24(7)
-	-	N(4)-Zn(1)-Cl(1)	113.76(7)
-	-	N(1)-Zn(1)-Cl(2)	114.91(7)
-	-	N(4)-Zn(1)-Cl(2)	105.77(7)
-	-	Cl(1)-Zn(1)-Cl(2)	110.74(3)

**Table 5:** UV-Vis absorption in ethanol, molar extinction coefficient (ε), as well as photoluminescent, decay lifetimes (τ), CIE (x, y) coordinates data in the solid state and ethanol of **ImL1**, **ImL2**, **ZnCl_2_(ImL1)_2_**, and **ZnCl_2_(ImL2)_2_** at room temperature.

Compounds	UV-Vis Absorption λmax, nm	Molar Extinction Coeffecient (ε = M^−1^cm^−1^)	PL Solid				PL in Ethanol Solution	
			*Λ*max ^1^ (nm) (Excitation)	*λ*max (nm) (Emission)	Decays Lifetime τ ^3^ [ns] ^4^	CIE coordinates (x,y)	*λ*max (nm) (Emission)	CIE Coordinates (x,y)
**ImL1**	210sh ^2^ 230 295sh 340	42,400 50,530 92,645 119,415	300	488	1.07	0.27, 0.333	366	0.239. 0.216
**ImL2**	215sh 230 295sh 340	25,655 31,660 51,390 73,975	300	411	7.81	0.196, 0.152	350	0.308, 0.23
**ZnCl_2_**(**ImL1**)**_2_**	210sh 235sh 295 335	31,005 31,910 64,610 91,685	300	410	9.42	0.239, 0.224	372, 399	0.303, 0.219
**ZnCl_2_**(**ImL2**)**_2_**	210 240 260sh 290 315	52,480 95,160 72,905 113,490 115,805	300	395	4.61	0.21, 0.193	345	0.342, 03.31

Abbreviation and symbol: ^1^ λmax = wavelength maximum, ^2^ Sh = shoulder, ^3^ τ = fluorescence decay lifetime, ^4^ ns = nanoseconds.

## Data Availability

The data presented in this study are available on request from the corresponding author.

## Supplementary Materials

The following supporting information can be downloaded at: https://www.mdpi.com/article/10.3390/ijms241512260/s1.
