# Supplementary material for: Novel Fluorescent Tetrahedral Zinc (II) Complexes Derived from 4-Phenyl-1-octyl-1H-imidazole Fused with Aryl-9H-Carbazole and Triarylamine Donor Units: Synthesis, Crystal Structures, and Photophysical Properties
Source: Int J Mol Sci. 2023 Jul 31;24(15):12260. doi: 10.3390/ijms241512260 (PMC10418610; doi:10.3390/ijms241512260)
Supplement: Supplementary file 1 [file ijms-24-12260-s001.zip › ijms-2492368-supplementary.pdf]

International Journal of *Molecular Sciences*

MDPI

Article supplementary information

Novel Fluorescent Tetrahedral Zinc (II) complexes derived from 4-Phenyl-1-octyl-1*H*-imidazole Fused with Aryl-9*H*-carbazole and Triarylamine Donor Units: Synthesis, Crystal Structures, and Photophysical Properties

Thompho Jason Rashamuse [1]*, Elena Mabel Coyanis [1], Rudolph Erasmus [2], Nomampondo Penelope Magwa [3]

[1] Nanotechnology Innovation Centre, Health Platform, Advanced Materials Division, Mintek, Private Bag X3015, Randburg, 2125, South Africa; jasonr@mintek.co.za (T.J.R) and mabelc@mintek.co.za (E.M.C)

[2] Materials for Energy Research group, Material Physics Research Institute, School of Physics, University of the Witwatersrand, Private Bag 3, Wits, 2050 Johannesburg, South Africa; Rudolph.Erasmus@wits.ac.za (R.E)

[3] Department of chemistry, University of South Africa, Private Bag X6, Florida, Roodepoort, Gauteng, 1710, South Africa; magwanp@unisa.ac.za.(N.P.M)

* Correspondence: jasonr@mintek.co.za; Tel.: (+27117094492)

Table of contents

1. NMR spectra of ligands and their correspoing Zn (II) complexes --------------------------- 2

2. FTIR spectra of the synthesised free ligands and their corresponding Zn (II) complexes --- 6

3. Single crystal X-ray diffraction full data set of 4'-(1-octyl-1H-imidazol-4-yl)-N,N-diphenyl-[1,1'-biphenyl]-4-yl)-4-amine ImL1 -- 8

4. Single crystal X-ray diffraction full date set of 9-(4'-(1-octyl-1H-imidazol-4-yl)-[1,1'-biphenyl]-4-yl)-9H-carbazole ImL2 --- 19

5. Single crystal X-ray diffraction full date set of $ZnCl_2(ImL1)_2$ complex--------------------- 30

6. Fluorescence decay curves -- 51

1. NMR spectra of ligands and their correspoing Zn (II) complexes

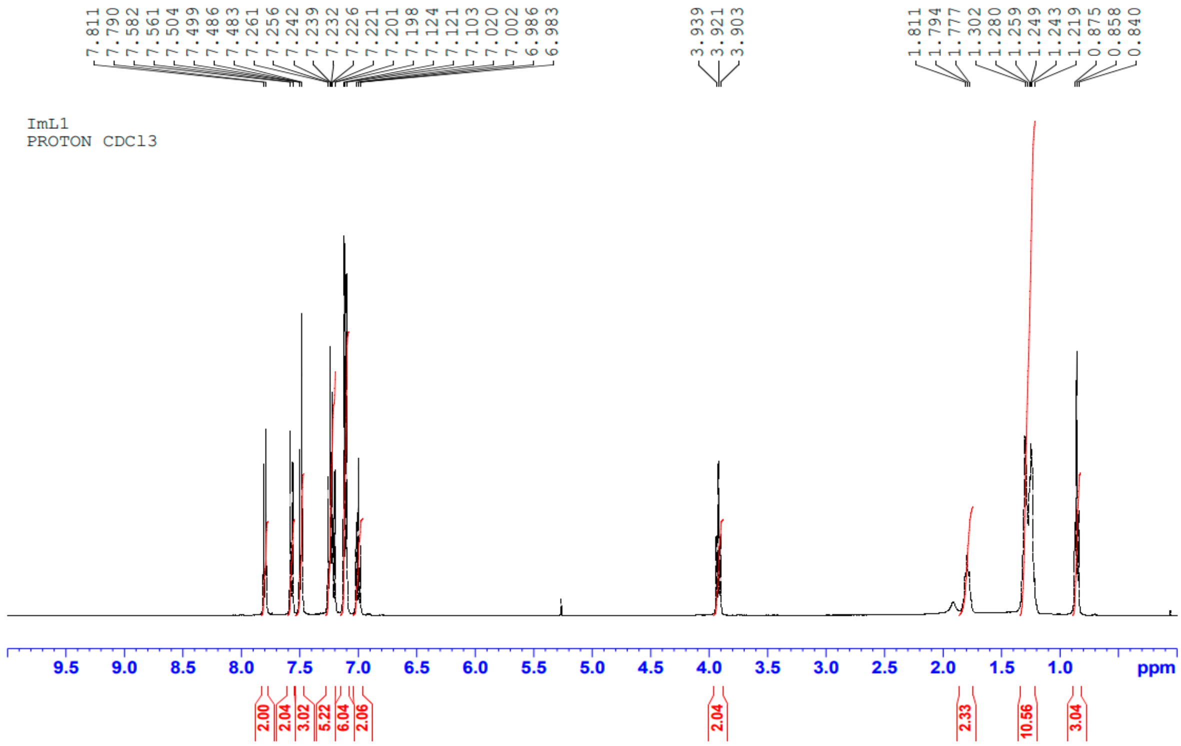

Figure S1**.** Proton NMR spectrum of 4'-(1-octyl-1*H*-imidazol-4-yl)-*N*,*N*-diphenyl-[1,1'-biphenyl]-4-yl)-4-amine **ImL1** in $CDCl_3$.

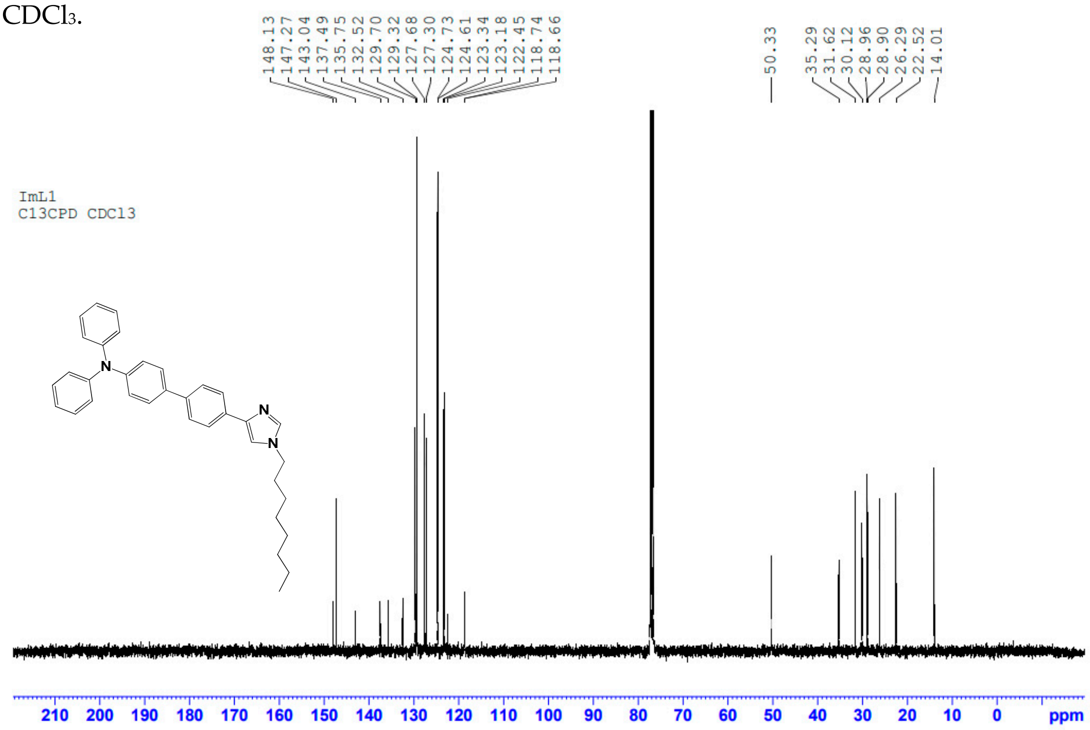

Figure S2. ^{13}C NMR spectrum of 4'-(1-octyl-1H-imidazol-4-yl)-*N*,*N*-diphenyl-[1,1'-biphenyl]-4-yl)-4-amine **ImL1** ($CDCl_3$, 101 MHz).

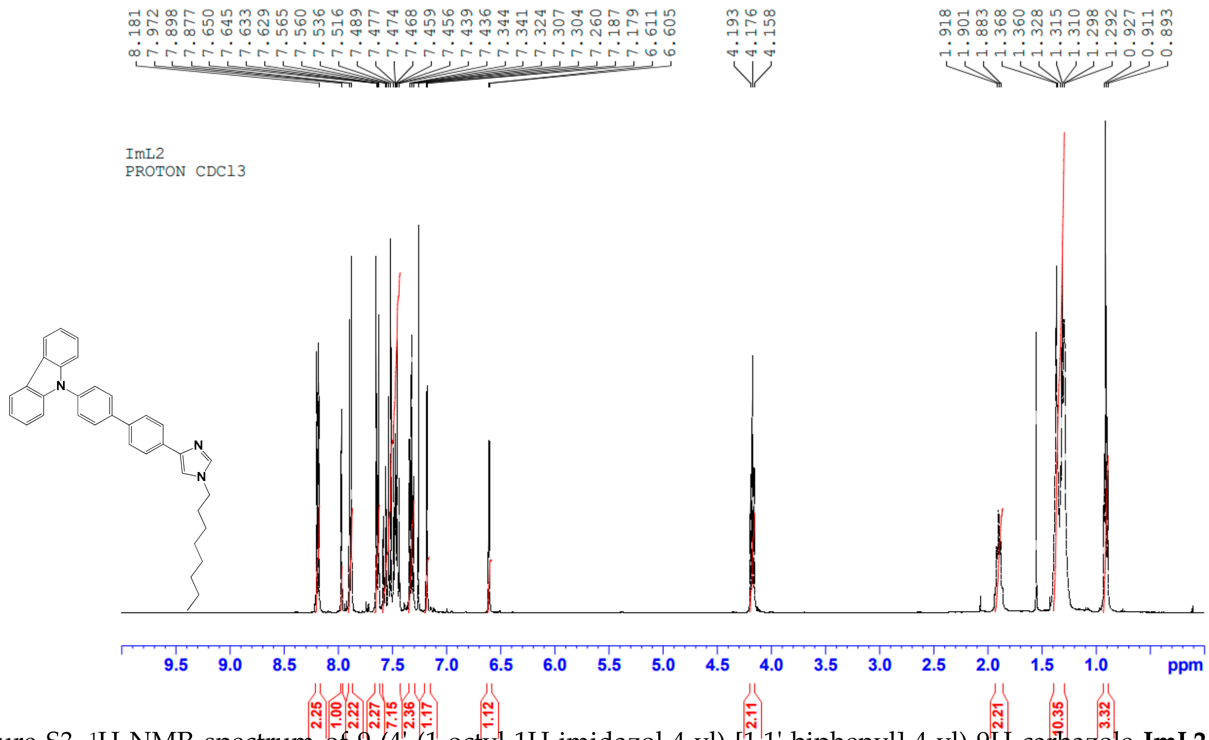

Figure S3. ¹H NMR spectrum of 9-(4'-(1-octyl-1H-imidazol-4-yl)-[1,1'-biphenyl]-4-yl)-9H-carbazole **ImL2** ($CDCl_3$, 400 MHz).

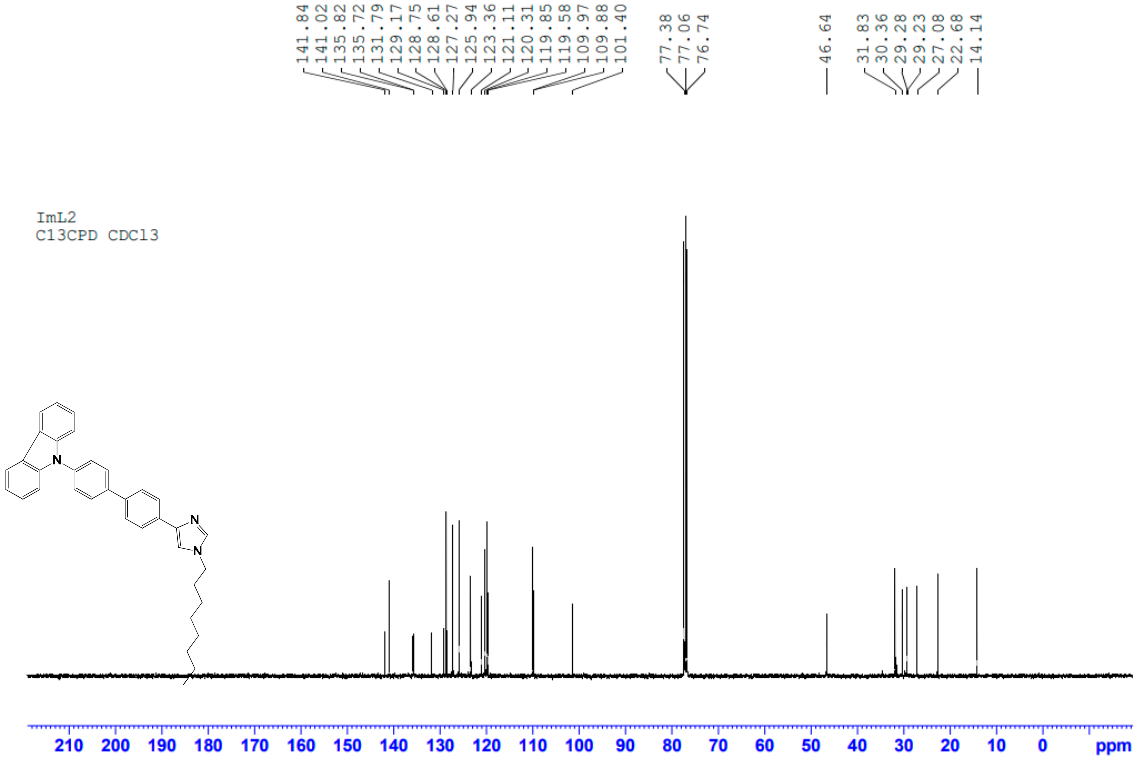

Figure S4. ¹³C NMR spectrum of 9-(4'-(1-octyl-1H-imidazol-4-yl)-[1,1'-biphenyl]-4-yl)-9H-carbazole **ImL2** ($CDCl_3$, 101 MHz).

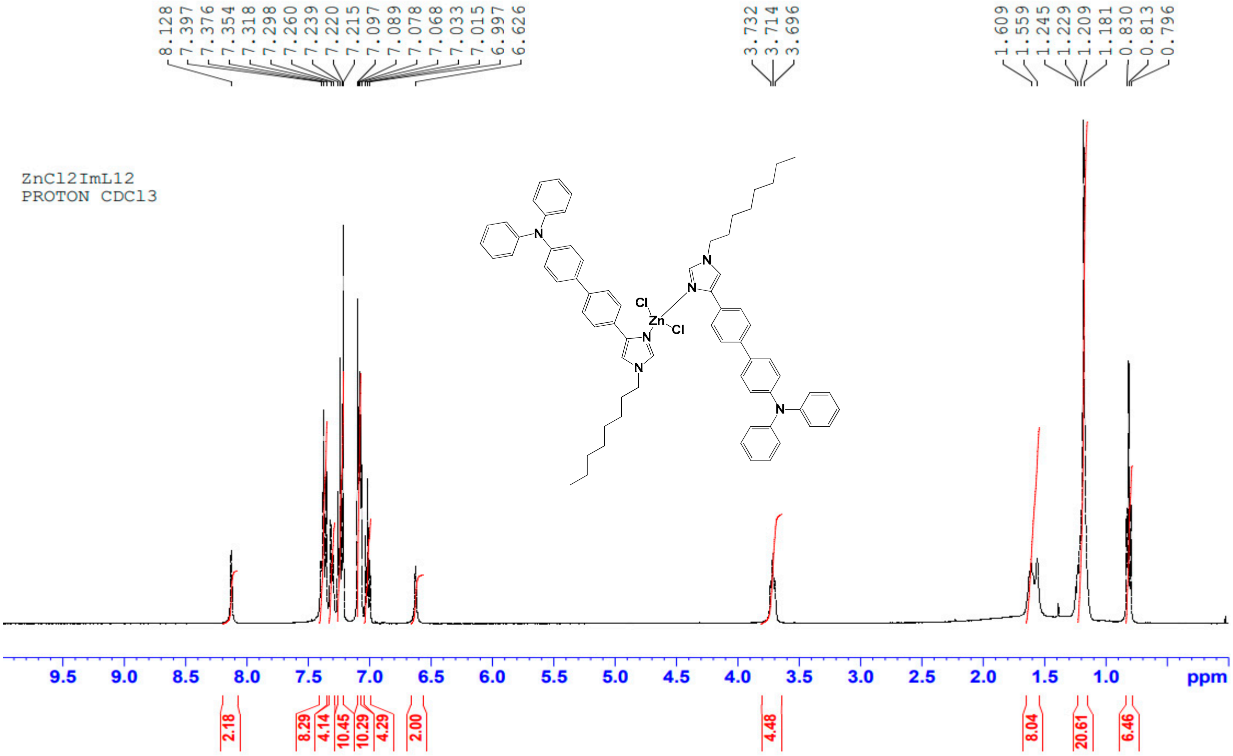

Figure S5. Proton NMR spectrum of **ZnCl₂(ImL1)₂** in CDCl₃.

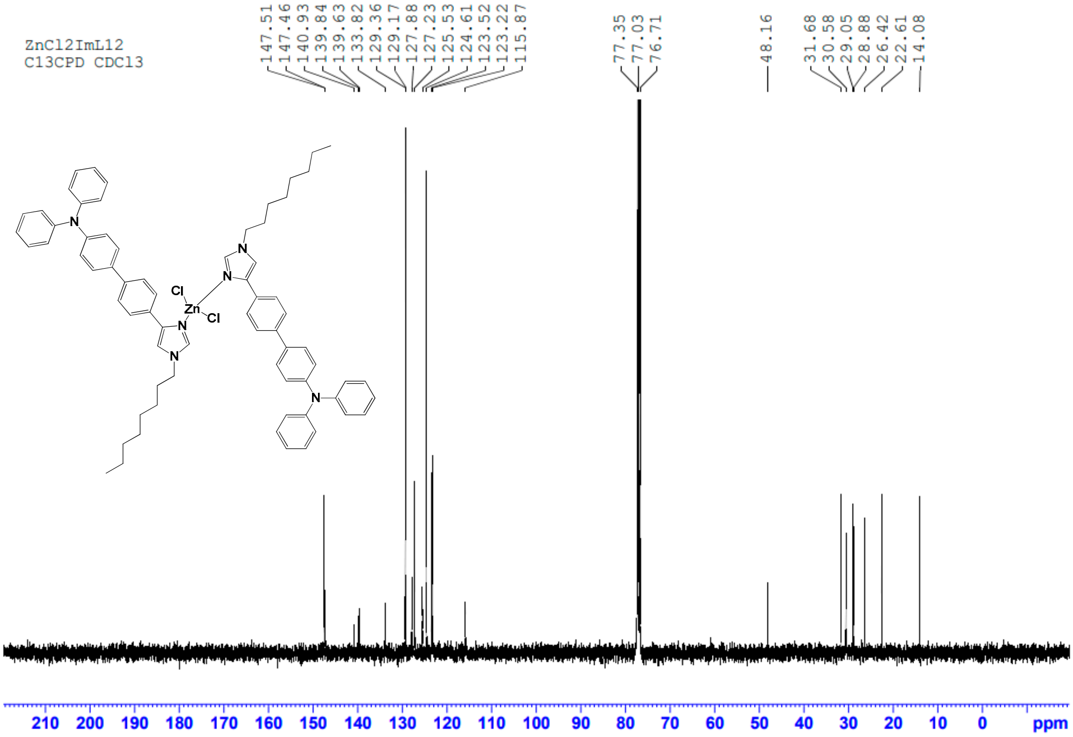

Figure S6. ^{13}C NMR spectrum of **$ZnCl_2(ImL1)_2$** complex ($CDCl_3$, 101 MHz).

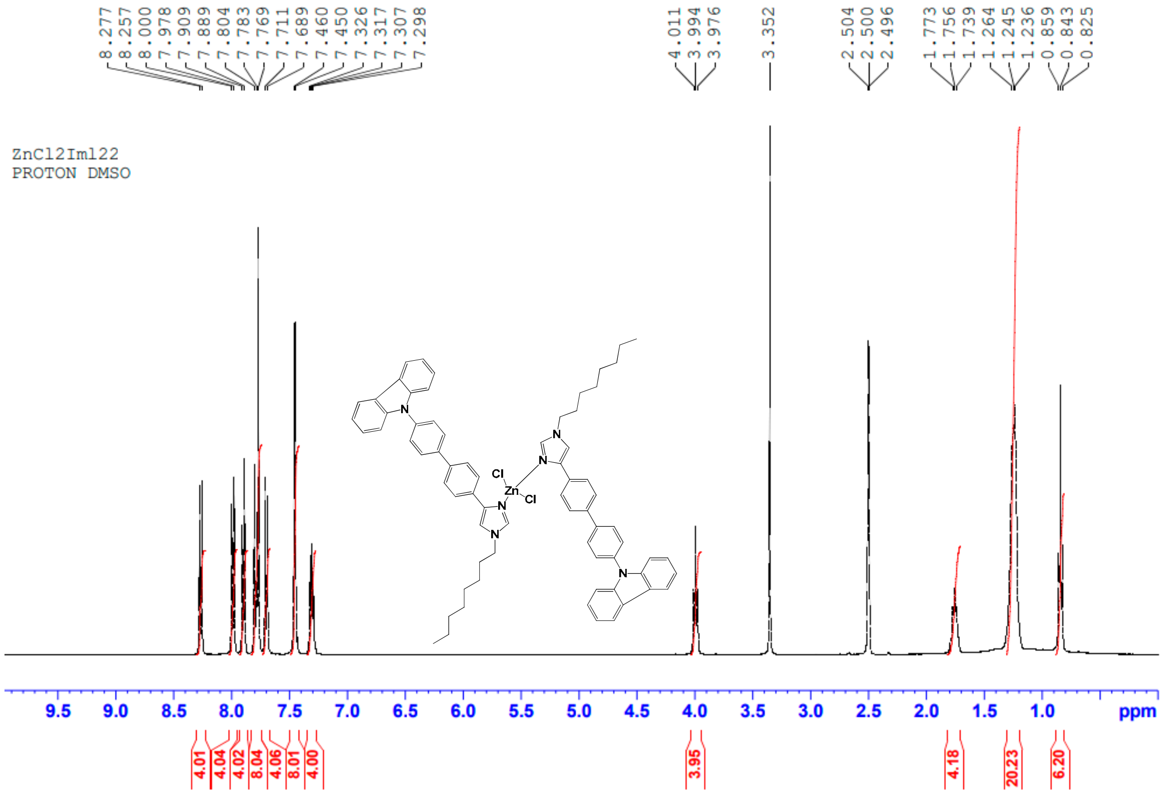

Figure S7. ^{1}H NMR spectrum of **ZnCl$_2$(ImL2)$_2$** complex (DMSO-*d6*, 400 MHz).

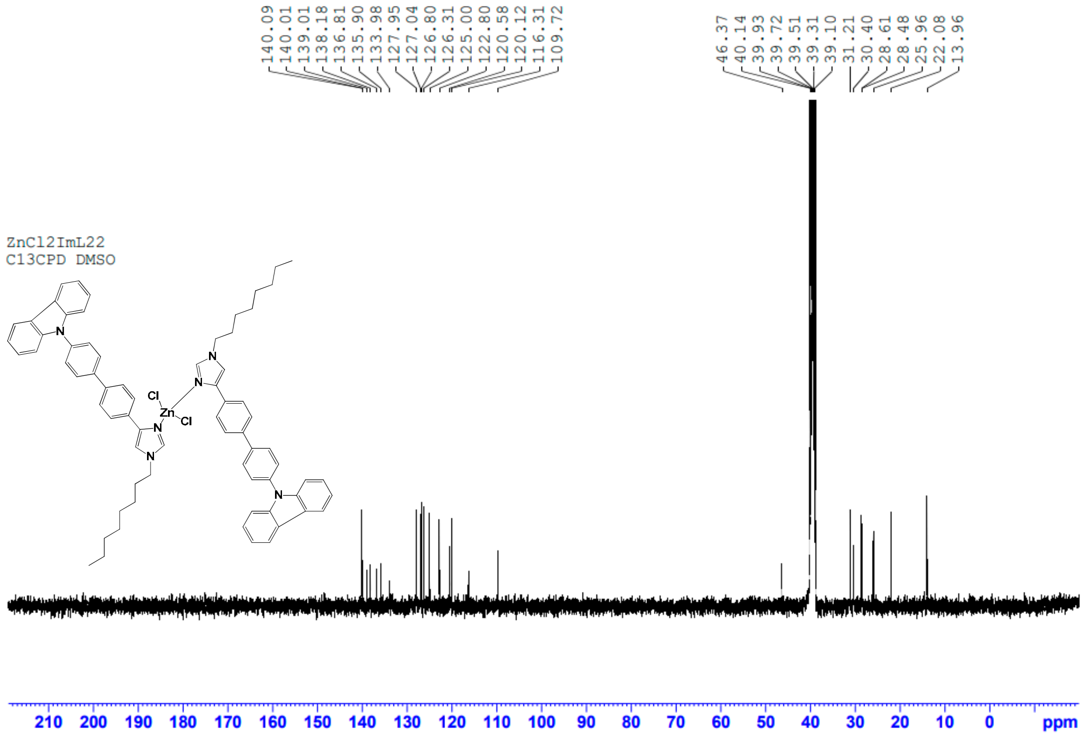

Figure S8. ^{13}C NMR spectrum of **ZnCl$_2$(ImL2)$_2$** complex (DMSO-*d6*, 101 MHz).

2. FTIR spectra of the synthesised free ligands and their corresponding Zn (II) complexes

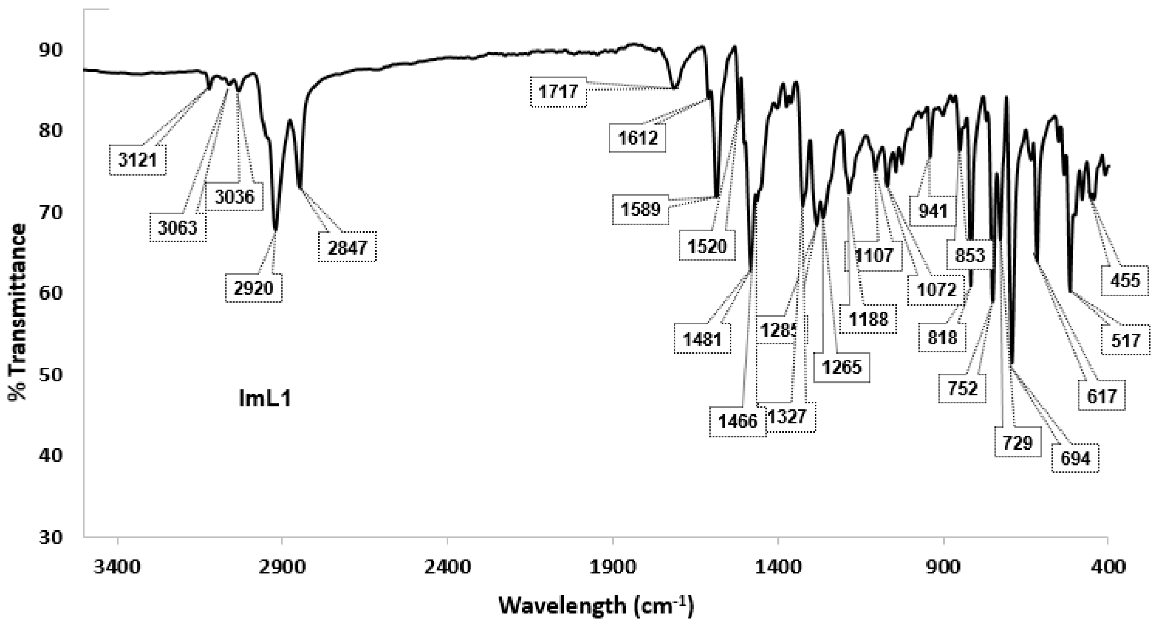

Figure S9. FTIR spectrum of the free ligand **ImL1**.

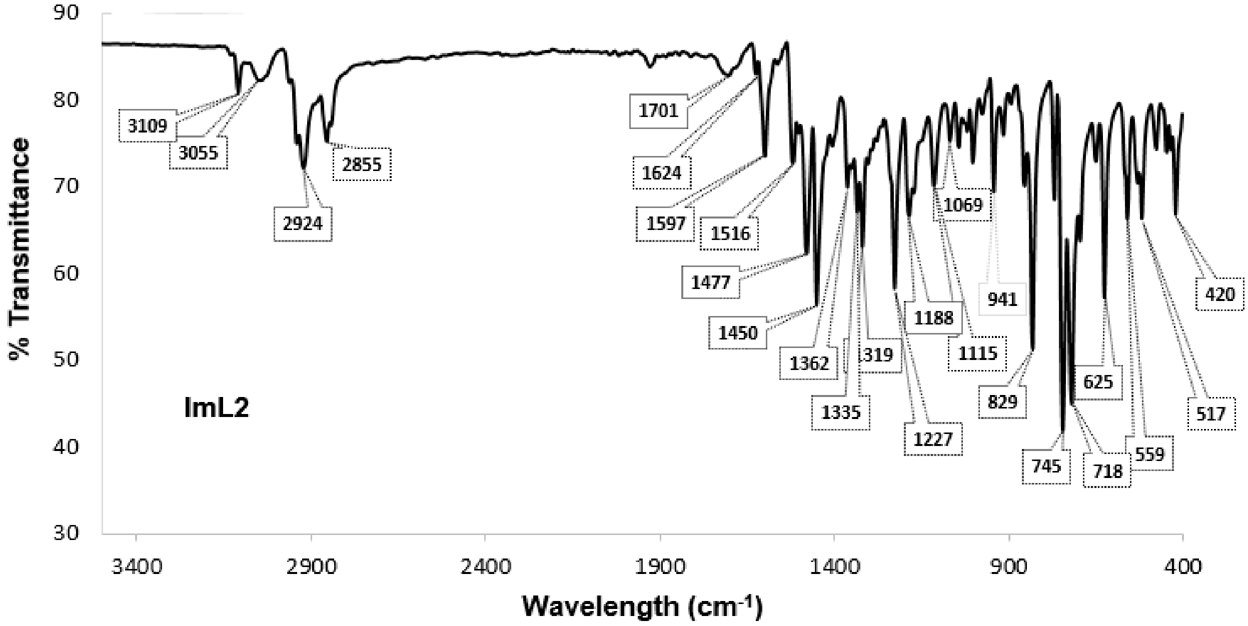

Figure S10. FTIR spectrum of the free ligand **ImL2**.

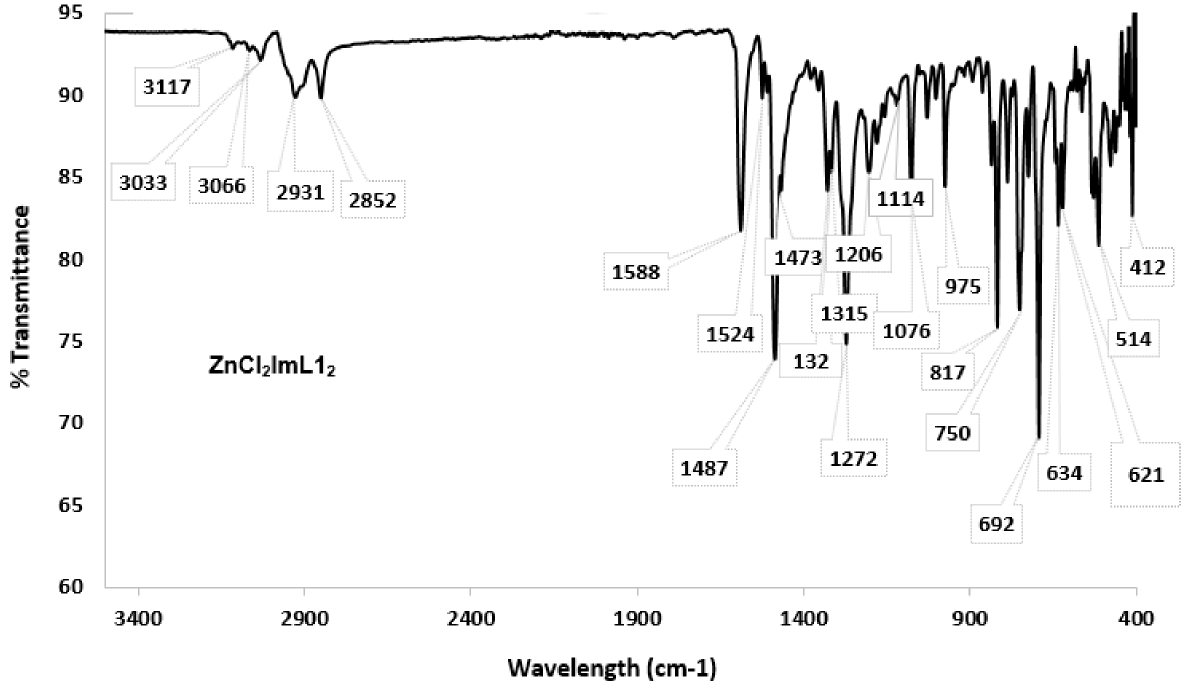

Figure S11. FTIR spectrum of the Zn (II) complex **ZnCl2(ImL1)2**.

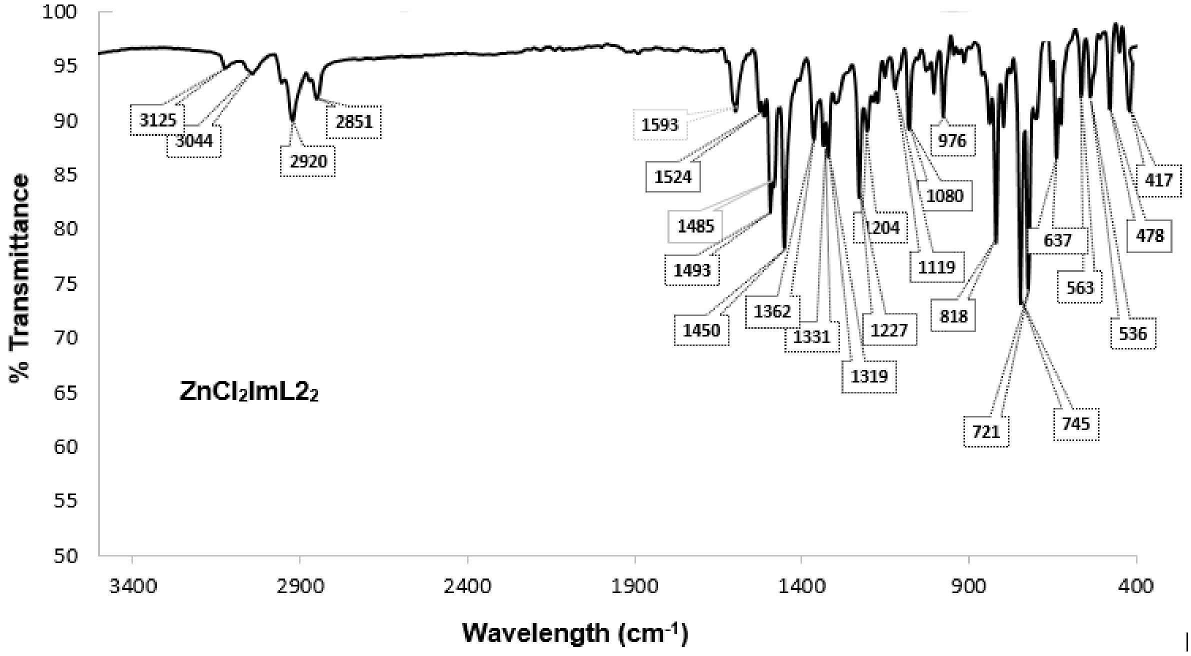

Figure S12. FTIR spectrum of the Zn (II) complex **ZnCl2(ImL2)2**.

3. Single crystal X-ray diffraction full data set of 4'-(1-octyl-1H-imidazol-4-yl)-*N*,*N*-diphenyl-[1,1'-biphenyl]-4-yl)-4-amine ImL1

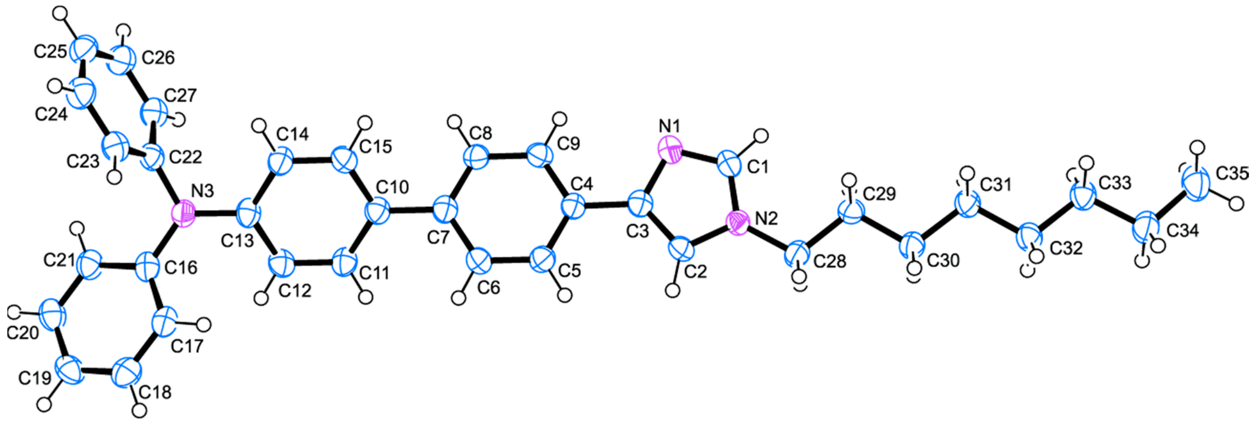

Figure 3 from the main text. Perspective view of **ImL1** (showing the atom-numbering scheme. Displacement ellipsoids are drawn at the 50% probability level and H atoms are shown as small spheres of arbitrary radii.

Table S1. Atomic coordinates (x 104) and equivalent isotropic displacement parameters (Å2x 103) for **ImL1.**

C(1)	**6173(6)**	**1576(4)**	**5038(1)**
C(2)	7374(5)	3832(4)	4717(1)
C(3)	5560(5)	3572(4)	4476(1)
C(4)	4481(5)	4553(4)	4051(1)
C(5)	5349(6)	5939(4)	3816(1)
C(6)	4383(6)	6832(4)	3399(1)
C(7)	2517(5)	6365(4)	3203(1)
C(8)	1630(5)	4990(4)	3443(1)
C(9)	2602(5)	4091(4)	3861(1)
C(10)	1544(5)	7296(4)	2744(1)
C(11)	1576(6)	8897(4)	2649(1)
C(12)	685(6)	9757(4)	2219(1)
C(13)	-205(5)	9025(4)	1845(1)
C(14)	-252(6)	7428(4)	1936(1)
C(15)	574(6)	6580(4)	2378(1)
C(16)	-532(5)	11399(4)	1180(1)
C(17)	1502(6)	12015(4)	1213(1)

Copyright: © 2023 by the authors. Licensee MDPI, Basel, Switzerland. This article is an open access article distributed under the terms and conditions of the Creative Commons Attribution (CC BY) license (https://creativecommons.org/licenses/by/4.0/).

C(18)	1891(6)	13526(4)	999(1)
C(19)	303(6)	14453(4)	741(1)
C(20)	-1696(6)	13838(4)	696(1)
C(21)	-2125(6)	12345(4)	912(1)
C(22)	-2340(5)	9084(4)	1081(1)
C(23)	-1527(6)	8914(4)	569(1)
C(24)	-2789(6)	8242(4)	254(1)
C(25)	-4836(6)	7712(4)	445(1)
C(26)	-5622(6)	7844(4)	958(1)
C(27)	-4385(5)	8538(4)	1273(1)
C(28)	9516(5)	2310(4)	5434(1)
C(29)	9570(5)	721(4)	5753(1)
C(30)	11421(5)	517(4)	6120(1)
C(31)	11526(6)	-1077(4)	6442(1)
C(32)	13315(6)	-1284(4)	6826(1)
C(33)	13194(6)	-2804(4)	7191(1)
C(34)	14866(6)	-3033(4)	7604(1)
C(35)	14535(7)	-4530(5)	7974(2)
N(1)	4787(5)	2137(3)	4680(1)
N(2)	7747(4)	2547(3)	5073(1)
N(3)	-1039(5)	9865(3)	1387(1)

Table S2. Bond lengths [Å] and angles [°] for **ImL1**.

**Bond lengths [Å]**	
C(1)-N(1)	1.328(4)
C(1)-N(2)	1.337(4)
C(1)-H(1)	0.95
C(2)-C(3)	1.361(5)
C(2)-N(2)	1.368(4)
C(2)-H(2)	0.95
C(3)-N(1)	1.384(4)
C(3)-C(4)	1.470(4)
C(4)-C(9)	1.386(4)
C(4)-C(5)	1.389(5)
C(5)-C(6)	1.388(5)
C(5)-H(5)	0.95
C(6)-C(7)	1.389(5)
C(6)-H(6)	0.95
C(7)-C(8)	1.389(5)
C(7)-C(10)	1.486(4)
C(8)-C(9)	1.395(4)

C(8)-H(8)	0.95
C(9)-H(9)	0.95
C(10)-C(11)	1.391(5)
C(10)-C(15)	1.398(5)
C(11)-C(12)	1.382(5)
C(11)-H(11)	0.95
C(12)-C(13)	1.398(5)
C(12)-H(12)	0.95
C(13)-C(14)	1.388(5)
C(13)-N(3)	1.418(4)
C(14)-C(15)	1.385(5)
C(14)-H(14)	0.95
C(15)-H(15)	0.95
C(16)-C(17)	1.393(5)
C(16)-C(21)	1.400(4)
C(16)-N(3)	1.414(4)
C(17)-C(18)	1.384(5)
C(17)-H(17)	0.95
C(18)-C(19)	1.378(5)
C(18)-H(18)	0.95
C(19)-C(20)	1.379(5)
C(19)-H(19)	0.95
C(20)-C(21)	1.378(5)
C(20)-H(20)	0.95
C(21)-H(21)	0.95
C(22)-C(27)	1.382(5)
C(22)-C(23)	1.396(5)
C(22)-N(3)	1.434(4)
C(23)-C(24)	1.380(5)
C(23)-H(23)	0.95
C(24)-C(25)	1.379(5)
C(24)-H(24)	0.95
C(25)-C(26)	1.385(5)
C(25)-H(25)	0.95
C(26)-C(27)	1.381(5)
C(26)-H(26)	0.95
C(27)-H(27)	0.95
C(28)-N(2)	1.471(4)
C(28)-C(29)	1.511(4)
C(28)-H(28A)	0.99
C(28)-H(28B)	0.99

C(29)-C(30)	1.521(4)
C(29)-H(29A)	0.99
C(29)-H(29B)	0.99
C(30)-C(31)	1.520(4)
C(30)-H(30A)	0.99
C(30)-H(30B)	0.99
C(31)-C(32)	1.521(4)
C(31)-H(31A)	0.99
C(31)-H(31B)	0.99
C(32)-C(33)	1.517(5)
C(32)-H(32A)	0.99
C(32)-H(32B)	0.99
C(33)-C(34)	1.524(5)
C(33)-H(33A)	0.99
C(33)-H(33B)	0.99
C(34)-C(35)	1.519(5)
C(34)-H(34A)	0.99
C(34)-H(34B)	0.99
C(35)-H(35A)	0.98
C(35)-H(35B)	0.98
C(35)-H(35C)	0.98
**Bond angles [°]**	
N(1)-C(1)-N(2)	112.6(3)
N(1)-C(1)-H(1)	123.7
N(2)-C(1)-H(1)	123.7
C(3)-C(2)-N(2)	106.4(3)
C(3)-C(2)-H(2)	126.8
N(2)-C(2)-H(2)	126.8
C(2)-C(3)-N(1)	109.8(3)
C(2)-C(3)-C(4)	129.3(3)
N(1)-C(3)-C(4)	120.9(3)
C(9)-C(4)-C(5)	118.1(3)
C(9)-C(4)-C(3)	120.6(3)
C(5)-C(4)-C(3)	121.3(3)
C(6)-C(5)-C(4)	121.2(3)
C(6)-C(5)-H(5)	119.4
C(4)-C(5)-H(5)	119.4
C(5)-C(6)-C(7)	121.0(3)
C(5)-C(6)-H(6)	119.5
C(7)-C(6)-H(6)	119.5
C(6)-C(7)-C(8)	117.8(3)

C(6)-C(7)-C(10)	120.7(3)
C(8)-C(7)-C(10)	121.5(3)
C(7)-C(8)-C(9)	121.3(3)
C(7)-C(8)-H(8)	119.4
C(9)-C(8)-H(8)	119.4
C(4)-C(9)-C(8)	120.7(3)
C(4)-C(9)-H(9)	119.7
C(8)-C(9)-H(9)	119.7
C(11)-C(10)-C(15)	116.7(3)
C(11)-C(10)-C(7)	122.6(3)
C(15)-C(10)-C(7)	120.7(3)
C(12)-C(11)-C(10)	122.4(3)
C(12)-C(11)-H(11)	118.8
C(10)-C(11)-H(11)	118.8
C(11)-C(12)-C(13)	120.4(3)
C(11)-C(12)-H(12)	119.8
C(13)-C(12)-H(12)	119.8
C(14)-C(13)-C(12)	117.7(3)
C(14)-C(13)-N(3)	120.4(3)
C(12)-C(13)-N(3)	121.9(3)
C(15)-C(14)-C(13)	121.5(3)
C(15)-C(14)-H(14)	119.3
C(13)-C(14)-H(14)	119.3
C(14)-C(15)-C(10)	121.3(3)
C(14)-C(15)-H(15)	119.3
C(10)-C(15)-H(15)	119.3
C(17)-C(16)-C(21)	117.6(3)
C(17)-C(16)-N(3)	123.7(3)
C(21)-C(16)-N(3)	118.7(3)
C(18)-C(17)-C(16)	120.6(3)
C(18)-C(17)-H(17)	119.7
C(16)-C(17)-H(17)	119.7
C(19)-C(18)-C(17)	121.3(4)
C(19)-C(18)-H(18)	119.4
C(17)-C(18)-H(18)	119.4
C(18)-C(19)-C(20)	118.4(3)
C(18)-C(19)-H(19)	120.8
C(20)-C(19)-H(19)	120.8
C(21)-C(20)-C(19)	121.1(3)
C(21)-C(20)-H(20)	119.4
C(19)-C(20)-H(20)	119.4

C(20)-C(21)-C(16)	120.9(3)
C(20)-C(21)-H(21)	119.6
C(16)-C(21)-H(21)	119.6
C(27)-C(22)-C(23)	119.6(3)
C(27)-C(22)-N(3)	122.0(3)
C(23)-C(22)-N(3)	118.4(3)
C(24)-C(23)-C(22)	119.9(3)
C(24)-C(23)-H(23)	120
C(22)-C(23)-H(23)	120
C(25)-C(24)-C(23)	120.3(3)
C(25)-C(24)-H(24)	119.8
C(23)-C(24)-H(24)	119.8
C(24)-C(25)-C(26)	119.9(3)
C(24)-C(25)-H(25)	120.1
C(26)-C(25)-H(25)	120.1
C(27)-C(26)-C(25)	120.2(3)
C(27)-C(26)-H(26)	119.9
C(25)-C(26)-H(26)	119.9
C(26)-C(27)-C(22)	120.1(3)
C(26)-C(27)-H(27)	119.9
C(22)-C(27)-H(27)	119.9
N(2)-C(28)-C(29)	113.0(3)
N(2)-C(28)-H(28A)	109
C(29)-C(28)-H(28A)	109
N(2)-C(28)-H(28B)	109
C(29)-C(28)-H(28B)	109
H(28A)-C(28)-H(28B)	107.8
C(28)-C(29)-C(30)	111.5(3)
C(28)-C(29)-H(29A)	109.3
C(30)-C(29)-H(29A)	109.3
C(28)-C(29)-H(29B)	109.3
C(30)-C(29)-H(29B)	109.3
H(29A)-C(29)-H(29B)	108
C(31)-C(30)-C(29)	112.7(3)
C(31)-C(30)-H(30A)	109.1
C(29)-C(30)-H(30A)	109.1
C(31)-C(30)-H(30B)	109.1
C(29)-C(30)-H(30B)	109.1
H(30A)-C(30)-H(30B)	107.8
C(30)-C(31)-C(32)	113.4(3)
C(30)-C(31)-H(31A)	108.9

C(32)-C(31)-H(31A)	108.9
C(30)-C(31)-H(31B)	108.9
C(32)-C(31)-H(31B)	108.9
H(31A)-C(31)-H(31B)	107.7
C(33)-C(32)-C(31)	112.3(3)
C(33)-C(32)-H(32A)	109.1
C(31)-C(32)-H(32A)	109.1
C(33)-C(32)-H(32B)	109.1
C(31)-C(32)-H(32B)	109.1
H(32A)-C(32)-H(32B)	107.9
C(32)-C(33)-C(34)	114.6(3)
C(32)-C(33)-H(33A)	108.6
C(34)-C(33)-H(33A)	108.6
C(32)-C(33)-H(33B)	108.6
C(34)-C(33)-H(33B)	108.6
H(33A)-C(33)-H(33B)	107.6
C(35)-C(34)-C(33)	111.2(3)
C(35)-C(34)-H(34A)	109.4
C(33)-C(34)-H(34A)	109.4
C(35)-C(34)-H(34B)	109.4
C(33)-C(34)-H(34B)	109.4
H(34A)-C(34)-H(34B)	108
C(34)-C(35)-H(35A)	109.5
C(34)-C(35)-H(35B)	109.5
H(35A)-C(35)-H(35B)	109.5
C(34)-C(35)-H(35C)	109.5
H(35A)-C(35)-H(35C)	109.5
H(35B)-C(35)-H(35C)	109.5
C(1)-N(1)-C(3)	104.3(3)
C(1)-N(2)-C(2)	106.9(3)
C(1)-N(2)-C(28)	127.0(3)
C(2)-N(2)-C(28)	126.1(3)
C(16)-N(3)-C(13)	124.7(3)
C(16)-N(3)-C(22)	116.6(3)
C(13)-N(3)-C(22)	118.5(3)

Table S3. Anisotropic displacement parameters (Å2x 103) for **ImL1**.

	U^{11}	U^{22}	U^{33}	U^{23}	U^{13}	U^{12}

C(1)	41(2)	30(2)	43(2)	3(1)	-9(2)	-1(2)
C(2)	40(2)	25(2)	38(2)	-4(1)	-5(1)	0(1)
C(3)	33(2)	31(2)	33(2)	-9(1)	-2(1)	6(1)
C(4)	33(2)	29(2)	31(2)	-8(1)	-4(1)	5(1)
C(5)	40(2)	33(2)	41(2)	-7(1)	-11(2)	1(2)
C(6)	43(2)	29(2)	40(2)	-2(1)	-8(2)	0(1)
C(7)	37(2)	33(2)	33(2)	-8(1)	-5(1)	5(1)
C(8)	35(2)	31(2)	38(2)	-6(1)	-7(1)	-1(1)
C(9)	34(2)	29(2)	38(2)	-5(1)	-2(1)	-2(1)
C(10)	35(2)	33(2)	35(2)	-6(1)	-5(1)	4(1)
C(11)	42(2)	36(2)	32(2)	-9(1)	-7(1)	6(2)
C(12)	43(2)	30(2)	37(2)	-8(1)	-4(1)	6(1)
C(13)	34(2)	35(2)	34(2)	-6(1)	-5(1)	9(1)
C(14)	40(2)	38(2)	34(2)	-7(1)	-8(1)	3(2)
C(15)	45(2)	29(2)	40(2)	-4(1)	-8(2)	1(2)
C(16)	40(2)	35(2)	28(2)	-7(1)	-4(1)	8(1)
C(17)	40(2)	41(2)	34(2)	-8(1)	-8(1)	6(2)
C(18)	46(2)	46(2)	41(2)	-13(2)	-5(2)	0(2)
C(19)	57(2)	29(2)	42(2)	-5(2)	-1(2)	1(2)
C(20)	45(2)	37(2)	44(2)	-3(2)	-6(2)	10(2)
C(21)	37(2)	39(2)	41(2)	-6(2)	-7(2)	6(2)
C(22)	39(2)	29(2)	36(2)	-4(1)	-7(1)	5(1)
C(23)	39(2)	37(2)	38(2)	-4(1)	-1(1)	5(2)
C(24)	56(2)	45(2)	35(2)	-10(2)	-4(2)	2(2)
C(25)	52(2)	40(2)	45(2)	-11(2)	-15(2)	2(2)
C(26)	35(2)	45(2)	51(2)	-6(2)	-7(2)	1(2)
C(27)	35(2)	39(2)	41(2)	-6(2)	-3(1)	7(2)
C(28)	34(2)	37(2)	37(2)	-7(1)	-9(1)	1(1)
C(29)	35(2)	31(2)	39(2)	-8(1)	-5(1)	-1(1)
C(30)	34(2)	36(2)	36(2)	-6(1)	-6(1)	2(1)

C(31)	38(2)	33(2)	39(2)	-8(1)	-8(1)	5(1)
C(32)	39(2)	35(2)	40(2)	-7(1)	-7(1)	5(2)
C(33)	43(2)	37(2)	38(2)	-8(2)	-8(2)	7(2)
C(34)	51(2)	46(2)	43(2)	-8(2)	-16(2)	6(2)
C(35)	71(3)	51(2)	41(2)	-6(2)	-16(2)	9(2)
N(1)	42(2)	35(2)	44(2)	2(1)	-12(1)	0(1)
N(2)	33(1)	28(1)	34(1)	-5(1)	-6(1)	3(1)
N(3)	41(2)	33(2)	34(1)	-4(1)	-10(1)	2(1)

Table S4. Hydrogen coordinates (x 10^4) and isotropic displacement parameters (Å^{2}x 10^3) for **ImL1**.

	**y**	**z**	**U(eq)**	**x**
H(1)	6057	593	5245	46
H(2)	8214	4729	4651	41
H(5)	6628	6282	3942	45
H(6)	5006	7778	3246	45
H(8)	337	4657	3321	41
H(9)	1970	3151	4017	41
H(11)	2235	9419	2889	44
H(12)	676	10852	2178	44
H(14)	-863	6904	1689	44
H(15)	479	5490	2433	46
H(17)	2632	11394	1384	46
H(18)	3280	13931	1030	52
H(19)	579	15490	597	52
H(20)	-2796	14455	512	51
H(21)	-3521	11951	879	47
H(23)	-106	9261	437	46
H(24)	-2245	8144	-97	54
H(25)	-5706	7257	227	53
H(26)	-7017	7457	1093	53
H(27)	-4939	8640	1623	46
H(28A)	9304	3093	5677	42
H(28B)	10959	2468	5229	42
H(29A)	9784	-68	5512	41
H(29B)	8134	560	5961	41
H(30A)	12851	690	5910	42
H(30B)	11197	1305	6360	42

H(31A)	11800	-1861	6201	44
H(31B)	10074	-1265	6641	44
H(32A)	14784	-1239	6626	45
H(32B)	13160	-424	7038	45
H(33A)	13426	-3658	6977	47
H(33B)	11687	-2871	7371	47
H(34A)	16383	-3051	7429	55
H(34B)	14704	-2155	7809	55
H(35A)	13108	-4461	8181	81
H(35B)	15728	-4705	8211	81
H(35C)	14557	-5389	7769	81

Table S5. Torsion angles [°] for **ImL1**.

N(2)-C(2)-C(3)-N(1)	0.3(3)
N(2)-C(2)-C(3)-C(4)	-178.0(3)
C(2)-C(3)-C(4)-C(9)	-178.9(3)
N(1)-C(3)-C(4)-C(9)	3.0(4)
C(2)-C(3)-C(4)-C(5)	3.3(5)
N(1)-C(3)-C(4)-C(5)	-174.8(3)
C(9)-C(4)-C(5)-C(6)	-0.6(5)
C(3)-C(4)-C(5)-C(6)	177.2(3)
C(4)-C(5)-C(6)-C(7)	-0.2(5)
C(5)-C(6)-C(7)-C(8)	1.1(5)
C(5)-C(6)-C(7)-C(10)	-177.5(3)
C(6)-C(7)-C(8)-C(9)	-1.3(5)
C(10)-C(7)-C(8)-C(9)	177.4(3)
C(5)-C(4)-C(9)-C(8)	0.5(5)
C(3)-C(4)-C(9)-C(8)	-177.4(3)
C(7)-C(8)-C(9)-C(4)	0.4(5)
C(6)-C(7)-C(10)-C(11)	-33.4(5)
C(8)-C(7)-C(10)-C(11)	147.9(3)
C(6)-C(7)-C(10)-C(15)	145.4(3)
C(8)-C(7)-C(10)-C(15)	-33.2(5)
C(15)-C(10)-C(11)-C(12)	0.5(5)
C(7)-C(10)-C(11)-C(12)	179.4(3)
C(10)-C(11)-C(12)-C(13)	-3.0(5)
C(11)-C(12)-C(13)-C(14)	3.0(5)
C(11)-C(12)-C(13)-N(3)	-177.0(3)
C(12)-C(13)-C(14)-C(15)	-0.7(5)
N(3)-C(13)-C(14)-C(15)	179.3(3)
C(13)-C(14)-C(15)-C(10)	-1.8(5)

C(11)-C(10)-C(15)-C(14)	1.8(5)
C(7)-C(10)-C(15)-C(14)	-177.1(3)
C(21)-C(16)-C(17)-C(18)	1.7(5)
N(3)-C(16)-C(17)-C(18)	179.6(3)
C(16)-C(17)-C(18)-C(19)	-1.1(5)
C(17)-C(18)-C(19)-C(20)	-0.5(5)
C(18)-C(19)-C(20)-C(21)	1.3(5)
C(19)-C(20)-C(21)-C(16)	-0.6(5)
C(17)-C(16)-C(21)-C(20)	-0.9(5)
N(3)-C(16)-C(21)-C(20)	-178.8(3)
C(27)-C(22)-C(23)-C(24)	1.7(5)
N(3)-C(22)-C(23)-C(24)	-176.5(3)
C(22)-C(23)-C(24)-C(25)	-1.1(5)
C(23)-C(24)-C(25)-C(26)	-0.5(5)
C(24)-C(25)-C(26)-C(27)	1.5(5)
C(25)-C(26)-C(27)-C(22)	-0.9(5)
C(23)-C(22)-C(27)-C(26)	-0.7(5)
N(3)-C(22)-C(27)-C(26)	177.5(3)
N(2)-C(28)-C(29)-C(30)	179.8(3)
C(28)-C(29)-C(30)-C(31)	-179.6(3)
C(29)-C(30)-C(31)-C(32)	-177.9(3)
C(30)-C(31)-C(32)-C(33)	172.6(3)
C(31)-C(32)-C(33)-C(34)	-176.7(3)
C(32)-C(33)-C(34)-C(35)	176.3(3)
N(2)-C(1)-N(1)-C(3)	0.0(4)
C(2)-C(3)-N(1)-C(1)	-0.2(4)
C(4)-C(3)-N(1)-C(1)	178.3(3)
N(1)-C(1)-N(2)-C(2)	0.2(4)
N(1)-C(1)-N(2)-C(28)	178.7(3)
C(3)-C(2)-N(2)-C(1)	-0.3(3)
C(3)-C(2)-N(2)-C(28)	-178.8(3)
C(29)-C(28)-N(2)-C(1)	5.6(4)
C(29)-C(28)-N(2)-C(2)	-176.2(3)
C(17)-C(16)-N(3)-C(13)	33.1(5)
C(21)-C(16)-N(3)-C(13)	-149.0(3)
C(17)-C(16)-N(3)-C(22)	-141.4(3)
C(21)-C(16)-N(3)-C(22)	36.5(4)
C(14)-C(13)-N(3)-C(16)	-161.3(3)
C(12)-C(13)-N(3)-C(16)	18.7(5)
C(14)-C(13)-N(3)-C(22)	13.1(4)
C(12)-C(13)-N(3)-C(22)	-166.9(3)

C(27)-C(22)-N(3)-C(16)	-119.5(3)
C(23)-C(22)-N(3)-C(16)	58.6(4)
C(27)-C(22)-N(3)-C(13)	65.6(4)
C(23)-C(22)-N(3)-C(13)	-116.2(3)

4. Single crystal X-ray diffraction full date set of 9-(4'-(1-octyl-1*H*-imidazol-4-yl)-[1,1'-biphenyl]-4-yl)-9*H*-carbazole **ImL2**

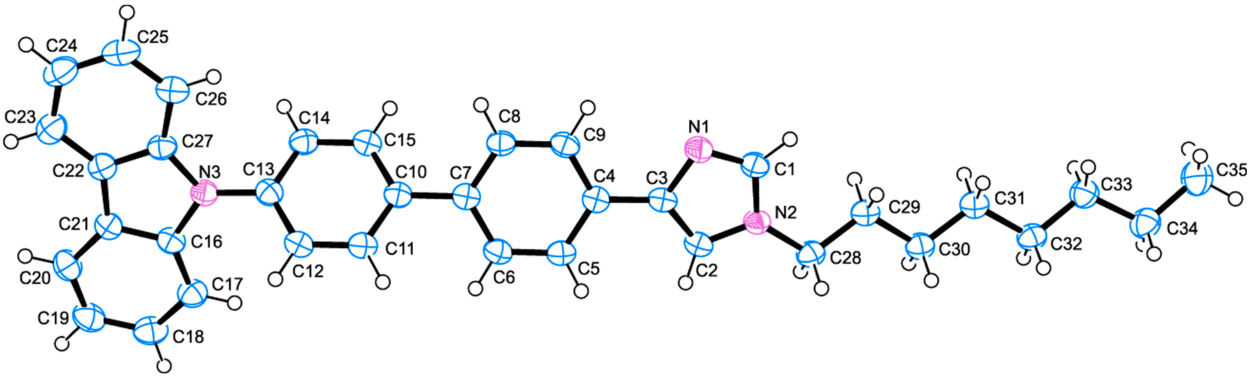

Figure 4 from the main text. Perspective view of **ImL2** showing the atom-numbering scheme. Displacement ellipsoids are drawn at the 50% probability level and H atoms are shown as small spheres of arbitrary radii.

Table S6. Atomic coordinates (x 104) and equivalent isotropic displacement parameters (Å2x 103) for **ImL2**.

	x	y	z	U(eq)
C(1)	5271(1)	6607(2)	4696(1)	36(1)
C(2)	5227(1)	8415(2)	3815(1)	36(1)
C(3)	4811(1)	7268(2)	3434(1)	32(1)
C(4)	4363(1)	7164(2)	2587(1)	31(1)
C(5)	4424(1)	8201(2)	1974(1)	35(1)
C(6)	3969(1)	8136(2)	1189(1)	36(1)
C(7)	3432(1)	7046(2)	977(1)	33(1)
C(8)	3387(1)	5991(2)	1584(1)	34(1)
C(9)	3846(1)	6044(2)	2369(1)	34(1)
C(10)	2920(1)	7036(2)	148(1)	32(1)
C(11)	3140(1)	7555(2)	-566(1)	36(1)
C(12)	2663(1)	7562(2)	-1336(1)	36(1)
C(13)	1937(1)	7079(2)	-1414(1)	35(1)
C(14)	1706(1)	6554(2)	-712(1)	36(1)
C(15)	2194(1)	6526(2)	58(1)	36(1)
C(16)	1352(1)	8378(2)	-2730(1)	34(1)
C(17)	1664(1)	9765(2)	-2588(1)	41(1)
C(18)	1444(1)	10814(2)	-3195(1)	45(1)
C(19)	928(1)	10513(2)	-3926(1)	46(1)

C(20)	618(1)	9140(2)	-4063(1)	42(1)
C(21)	829(1)	8052(2)	-3461(1)	34(1)
C(22)	599(1)	6560(2)	-3385(1)	35(1)
C(23)	104(1)	5643(2)	-3907(1)	44(1)
C(24)	-3(1)	4243(2)	-3643(1)	50(1)
C(25)	383(1)	3742(2)	-2869(1)	47(1)
C(26)	881(1)	4622(2)	-2341(1)	41(1)
C(27)	980(1)	6042(2)	-2606(1)	34(1)
C(28)	6015(1)	8859(2)	5255(1)	39(1)
C(29)	6116(1)	8203(2)	6121(1)	41(1)
C(30)	6632(1)	9104(2)	6777(1)	40(1)
C(31)	6736(1)	8432(2)	7644(1)	44(1)
C(32)	7315(1)	9183(2)	8305(1)	39(1)
C(33)	7392(1)	8473(2)	9159(1)	42(1)
C(34)	8040(1)	9010(2)	9820(1)	46(1)
C(35)	8142(1)	8117(3)	10619(1)	63(1)
N(1)	4842(1)	6129(2)	4001(1)	37(1)
N(2)	5516(1)	7992(2)	4621(1)	34(1)
N(3)	1445(1)	7152(2)	-2207(1)	36(1)

Table S7. Bond lengths [Å] and angles [°] for **ImL2.**

**Bond lengths [Å]**	
C(1)-N(1)	1.318(2)
C(1)-N(2)	1.357(2)
C(1)-H(1)	0.95
C(2)-N(2)	1.369(2)
C(2)-C(3)	1.369(2)
C(2)-H(2)	0.95
C(3)-N(1)	1.386(2)
C(3)-C(4)	1.460(2)
C(4)-C(9)	1.393(2)
C(4)-C(5)	1.399(2)
C(5)-C(6)	1.381(2)
C(5)-H(5)	0.95
C(6)-C(7)	1.396(2)
C(6)-H(6)	0.95
C(7)-C(8)	1.396(2)
C(7)-C(10)	1.482(2)
C(8)-C(9)	1.383(2)
C(8)-H(8)	0.95

C(9)-H(9)	0.95
C(10)-C(11)	1.394(2)
C(10)-C(15)	1.396(2)
C(11)-C(12)	1.377(2)
C(11)-H(11)	0.95
C(12)-C(13)	1.389(2)
C(12)-H(12)	0.95
C(13)-C(14)	1.389(2)
C(13)-N(3)	1.420(2)
C(14)-C(15)	1.386(2)
C(14)-H(14)	0.95
C(15)-H(15)	0.95
C(16)-C(17)	1.391(2)
C(16)-N(3)	1.396(2)
C(16)-C(21)	1.403(2)
C(17)-C(18)	1.377(3)
C(17)-H(17)	0.95
C(18)-C(19)	1.392(3)
C(18)-H(18)	0.95
C(19)-C(20)	1.377(3)
C(19)-H(19)	0.95
C(20)-C(21)	1.394(2)
C(20)-H(20)	0.95
C(21)-C(22)	1.440(2)
C(22)-C(23)	1.393(2)
C(22)-C(27)	1.402(2)
C(23)-C(24)	1.375(3)
C(23)-H(23)	0.95
C(24)-C(25)	1.394(3)
C(24)-H(24)	0.95
C(25)-C(26)	1.380(3)
C(25)-H(25)	0.95
C(26)-C(27)	1.391(2)
C(26)-H(26)	0.95
C(27)-N(3)	1.398(2)
C(28)-N(2)	1.467(2)
C(28)-C(29)	1.512(2)
C(28)-H(28A)	0.99
C(28)-H(28B)	0.99
C(29)-C(30)	1.519(2)
C(29)-H(29A)	0.99

C(29)-H(29B)	0.99
C(30)-C(31)	1.519(2)
C(30)-H(30A)	0.99
C(30)-H(30B)	0.99
C(31)-C(32)	1.516(2)
C(31)-H(31A)	0.99
C(31)-H(31B)	0.99
C(32)-C(33)	1.518(2)
C(32)-H(32A)	0.99
C(32)-H(32B)	0.99
C(33)-C(34)	1.517(2)
C(33)-H(33A)	0.99
C(33)-H(33B)	0.99
C(34)-C(35)	1.516(3)
C(34)-H(34A)	0.99
C(34)-H(34B)	0.99
C(35)-H(35A)	0.98
C(35)-H(35B)	0.98
C(35)-H(35C)	0.98
**Bond angles [°]**	
N(1)-C(1)-N(2)	112.44(15)
N(1)-C(1)-H(1)	123.8
N(2)-C(1)-H(1)	123.8
N(2)-C(2)-C(3)	106.95(15)
N(2)-C(2)-H(2)	126.5
C(3)-C(2)-H(2)	126.5
C(2)-C(3)-N(1)	109.17(15)
C(2)-C(3)-C(4)	129.40(15)
N(1)-C(3)-C(4)	121.39(15)
C(9)-C(4)-C(5)	117.75(15)
C(9)-C(4)-C(3)	120.89(15)
C(5)-C(4)-C(3)	121.34(15)
C(6)-C(5)-C(4)	120.78(16)
C(6)-C(5)-H(5)	119.6
C(4)-C(5)-H(5)	119.6
C(5)-C(6)-C(7)	121.63(16)
C(5)-C(6)-H(6)	119.2
C(7)-C(6)-H(6)	119.2
C(6)-C(7)-C(8)	117.30(15)
C(6)-C(7)-C(10)	121.03(15)
C(8)-C(7)-C(10)	121.66(15)

C(9)-C(8)-C(7)	121.30(16)
C(9)-C(8)-H(8)	119.4
C(7)-C(8)-H(8)	119.3
C(8)-C(9)-C(4)	121.17(15)
C(8)-C(9)-H(9)	119.4
C(4)-C(9)-H(9)	119.4
C(11)-C(10)-C(15)	117.64(16)
C(11)-C(10)-C(7)	121.42(15)
C(15)-C(10)-C(7)	120.94(15)
C(12)-C(11)-C(10)	121.49(16)
C(12)-C(11)-H(11)	119.3
C(10)-C(11)-H(11)	119.3
C(11)-C(12)-C(13)	120.33(16)
C(11)-C(12)-H(12)	119.8
C(13)-C(12)-H(12)	119.8
C(12)-C(13)-C(14)	119.15(16)
C(12)-C(13)-N(3)	119.44(15)
C(14)-C(13)-N(3)	121.40(15)
C(15)-C(14)-C(13)	120.17(16)
C(15)-C(14)-H(14)	119.9
C(13)-C(14)-H(14)	119.9
C(14)-C(15)-C(10)	121.19(16)
C(14)-C(15)-H(15)	119.4
C(10)-C(15)-H(15)	119.4
C(17)-C(16)-N(3)	129.33(16)
C(17)-C(16)-C(21)	121.48(16)
N(3)-C(16)-C(21)	109.09(15)
C(18)-C(17)-C(16)	117.64(17)
C(18)-C(17)-H(17)	121.2
C(16)-C(17)-H(17)	121.2
C(17)-C(18)-C(19)	121.81(18)
C(17)-C(18)-H(18)	119.1
C(19)-C(18)-H(18)	119.1
C(20)-C(19)-C(18)	120.39(17)
C(20)-C(19)-H(19)	119.8
C(18)-C(19)-H(19)	119.8
C(19)-C(20)-C(21)	119.25(17)
C(19)-C(20)-H(20)	120.4
C(21)-C(20)-H(20)	120.4
C(20)-C(21)-C(16)	119.43(16)
C(20)-C(21)-C(22)	133.71(17)

C(16)-C(21)-C(22)	106.81(15)
C(23)-C(22)-C(27)	119.64(17)
C(23)-C(22)-C(21)	133.11(17)
C(27)-C(22)-C(21)	107.24(15)
C(24)-C(23)-C(22)	119.07(19)
C(24)-C(23)-H(23)	120.5
C(22)-C(23)-H(23)	120.5
C(23)-C(24)-C(25)	120.60(18)
C(23)-C(24)-H(24)	119.7
C(25)-C(24)-H(24)	119.7
C(26)-C(25)-C(24)	121.65(18)
C(26)-C(25)-H(25)	119.2
C(24)-C(25)-H(25)	119.2
C(25)-C(26)-C(27)	117.48(18)
C(25)-C(26)-H(26)	121.3
C(27)-C(26)-H(26)	121.3
C(26)-C(27)-N(3)	129.63(16)
C(26)-C(27)-C(22)	121.55(16)
N(3)-C(27)-C(22)	108.81(14)
N(2)-C(28)-C(29)	112.65(14)
N(2)-C(28)-H(28A)	109.1
C(29)-C(28)-H(28A)	109.1
N(2)-C(28)-H(28B)	109.1
C(29)-C(28)-H(28B)	109.1
H(28A)-C(28)-H(28B)	107.8
C(28)-C(29)-C(30)	112.54(15)
C(28)-C(29)-H(29A)	109.1
C(30)-C(29)-H(29A)	109.1
C(28)-C(29)-H(29B)	109.1
C(30)-C(29)-H(29B)	109.1
H(29A)-C(29)-H(29B)	107.8
C(31)-C(30)-C(29)	112.23(15)
C(31)-C(30)-H(30A)	109.2
C(29)-C(30)-H(30A)	109.2
C(31)-C(30)-H(30B)	109.2
C(29)-C(30)-H(30B)	109.2
H(30A)-C(30)-H(30B)	107.9
C(32)-C(31)-C(30)	114.49(15)
C(32)-C(31)-H(31A)	108.6
C(30)-C(31)-H(31A)	108.6
C(32)-C(31)-H(31B)	108.6

C(30)-C(31)-H(31B)	108.6
H(31A)-C(31)-H(31B)	107.6
C(31)-C(32)-C(33)	112.23(15)
C(31)-C(32)-H(32A)	109.2
C(33)-C(32)-H(32A)	109.2
C(31)-C(32)-H(32B)	109.2
C(33)-C(32)-H(32B)	109.2
H(32A)-C(32)-H(32B)	107.9
C(34)-C(33)-C(32)	115.21(16)
C(34)-C(33)-H(33A)	108.5
C(32)-C(33)-H(33A)	108.5
C(34)-C(33)-H(33B)	108.5
C(32)-C(33)-H(33B)	108.5
H(33A)-C(33)-H(33B)	107.5
C(35)-C(34)-C(33)	112.18(17)
C(35)-C(34)-H(34A)	109.2
C(33)-C(34)-H(34A)	109.2
C(35)-C(34)-H(34B)	109.2
C(33)-C(34)-H(34B)	109.2
H(34A)-C(34)-H(34B)	107.9
C(34)-C(35)-H(35A)	109.5
C(34)-C(35)-H(35B)	109.5
H(35A)-C(35)-H(35B)	109.5
C(34)-C(35)-H(35C)	109.5
H(35A)-C(35)-H(35C)	109.5
H(35B)-C(35)-H(35C)	109.5
C(1)-N(1)-C(3)	105.19(14)
C(1)-N(2)-C(2)	106.25(14)
C(1)-N(2)-C(28)	127.62(14)
C(2)-N(2)-C(28)	126.09(14)
C(16)-N(3)-C(27)	108.04(13)
C(16)-N(3)-C(13)	124.71(14)
C(27)-N(3)-C(13)	127.25(14)

Table S8. Anisotropic displacement parameters (Å2x 103) for **ImL2**.

	U^{11}	U^{22}	U^{33}	U^{23}	U^{13}	U^{12}
C(1)	41(1)	29(1)	40(1)	4(1)	8(1)	-1(1)
C(2)	41(1)	31(1)	38(1)	5(1)	11(1)	-1(1)
C(3)	34(1)	27(1)	38(1)	1(1)	14(1)	2(1)
C(4)	32(1)	28(1)	35(1)	-1(1)	11(1)	5(1)

C(5)	36(1)	31(1)	41(1)	1(1)	11(1)	-2(1)
C(6)	37(1)	32(1)	39(1)	5(1)	11(1)	1(1)
C(7)	32(1)	30(1)	37(1)	0(1)	11(1)	4(1)
C(8)	37(1)	28(1)	39(1)	0(1)	11(1)	-2(1)
C(9)	40(1)	26(1)	39(1)	3(1)	13(1)	3(1)
C(10)	35(1)	27(1)	37(1)	1(1)	10(1)	3(1)
C(11)	35(1)	33(1)	42(1)	3(1)	11(1)	0(1)
C(12)	40(1)	34(1)	37(1)	4(1)	12(1)	1(1)
C(13)	39(1)	28(1)	37(1)	0(1)	9(1)	2(1)
C(14)	33(1)	34(1)	43(1)	2(1)	10(1)	0(1)
C(15)	38(1)	34(1)	38(1)	4(1)	12(1)	0(1)
C(16)	38(1)	28(1)	37(1)	2(1)	11(1)	4(1)
C(17)	44(1)	32(1)	45(1)	0(1)	9(1)	0(1)
C(18)	48(1)	29(1)	62(1)	3(1)	15(1)	2(1)
C(19)	50(1)	37(1)	52(1)	13(1)	12(1)	7(1)
C(20)	44(1)	43(1)	39(1)	5(1)	8(1)	8(1)
C(21)	34(1)	35(1)	35(1)	-1(1)	11(1)	3(1)
C(22)	34(1)	36(1)	37(1)	-4(1)	13(1)	1(1)
C(23)	41(1)	47(1)	45(1)	-8(1)	12(1)	-3(1)
C(24)	49(1)	47(1)	57(1)	-15(1)	18(1)	-14(1)
C(25)	50(1)	35(1)	64(1)	-7(1)	28(1)	-8(1)
C(26)	45(1)	35(1)	48(1)	1(1)	18(1)	-1(1)
C(27)	36(1)	30(1)	40(1)	-3(1)	13(1)	-2(1)
C(28)	42(1)	33(1)	41(1)	-2(1)	8(1)	-6(1)
C(29)	43(1)	38(1)	42(1)	2(1)	8(1)	-7(1)
C(30)	43(1)	34(1)	42(1)	0(1)	8(1)	-3(1)
C(31)	45(1)	40(1)	45(1)	3(1)	6(1)	-6(1)
C(32)	39(1)	32(1)	45(1)	-2(1)	10(1)	-2(1)
C(33)	44(1)	42(1)	41(1)	-2(1)	8(1)	-2(1)
C(34)	48(1)	42(1)	48(1)	-5(1)	7(1)	-4(1)
C(35)	68(2)	74(2)	42(1)	-4(1)	4(1)	-12(1)
N(1)	41(1)	29(1)	40(1)	3(1)	8(1)	0(1)
N(2)	37(1)	28(1)	36(1)	1(1)	8(1)	-2(1)
N(3)	40(1)	30(1)	37(1)	3(1)	6(1)	-1(1)

Table S9. Hydrogen coordinates (x 104) and isotropic displacement parameters (Å2x 103) for **ImL2.**

	x	y	z	U(eq)
H(1)	5395	6045	5195	44
H(2)	5300	9331	3568	43
H(5)	4783	8959	2099	42

H(6)	4022	8850	782	43
H(8)	3035	5222	1455	41
H(9)	3807	5304	2767	41
H(11)	3631	7912	-521	44
H(12)	2830	7898	-1815	44
H(14)	1212	6213	-759	43
H(15)	2031	6152	532	43
H(17)	2016	9981	-2091	49
H(18)	1651	11769	-3112	55
H(19)	789	11260	-4333	56
H(20)	265	8936	-4561	51
H(23)	-157	5979	-4437	53
H(24)	-344	3613	-3992	60
H(25)	301	2770	-2700	57
H(26)	1145	4272	-1816	50
H(28A)	5812	9862	5264	46
H(28B)	6504	8935	5098	46
H(29A)	6319	7201	6112	49
H(29B)	5626	8127	6278	49
H(30A)	7121	9185	6619	48
H(30B)	6428	10104	6790	48
H(31A)	6255	8460	7825	52
H(31B)	6877	7391	7611	52
H(32A)	7178	10225	8343	46
H(32B)	7799	9148	8133	46
H(33A)	6929	8646	9363	51
H(33B)	7443	7402	9094	51
H(34A)	8499	8956	9596	56
H(34B)	7958	10049	9949	56
H(35A)	7694	8189	10852	94
H(35B)	8567	8496	11026	94
H(35C)	8230	7090	10495	94

Table S10. Torsion angles [°] for **ImL2**.

N(2)-C(2)-C(3)-N(1)	-0.10(19)
N(2)-C(2)-C(3)-C(4)	177.55(15)
C(2)-C(3)-C(4)-C(9)	-165.11(17)
N(1)-C(3)-C(4)-C(9)	12.3(2)
C(2)-C(3)-C(4)-C(5)	13.2(3)
N(1)-C(3)-C(4)-C(5)	-169.37(15)
C(9)-C(4)-C(5)-C(6)	2.0(2)

C(3)-C(4)-C(5)-C(6)	-176.40(15)
C(4)-C(5)-C(6)-C(7)	0.2(3)
C(5)-C(6)-C(7)-C(8)	-2.0(2)
C(5)-C(6)-C(7)-C(10)	176.65(15)
C(6)-C(7)-C(8)-C(9)	1.4(2)
C(10)-C(7)-C(8)-C(9)	-177.17(15)
C(7)-C(8)-C(9)-C(4)	0.8(3)
C(5)-C(4)-C(9)-C(8)	-2.5(2)
C(3)-C(4)-C(9)-C(8)	175.88(15)
C(6)-C(7)-C(10)-C(11)	32.3(2)
C(8)-C(7)-C(10)-C(11)	-149.19(16)
C(6)-C(7)-C(10)-C(15)	-146.78(16)
C(8)-C(7)-C(10)-C(15)	31.8(2)
C(15)-C(10)-C(11)-C(12)	-0.2(3)
C(7)-C(10)-C(11)-C(12)	-179.28(15)
C(10)-C(11)-C(12)-C(13)	1.6(3)
C(11)-C(12)-C(13)-C(14)	-1.7(3)
C(11)-C(12)-C(13)-N(3)	177.37(15)
C(12)-C(13)-C(14)-C(15)	0.5(3)
N(3)-C(13)-C(14)-C(15)	-178.55(15)
C(13)-C(14)-C(15)-C(10)	0.8(3)
C(11)-C(10)-C(15)-C(14)	-1.0(2)
C(7)-C(10)-C(15)-C(14)	178.08(15)
N(3)-C(16)-C(17)-C(18)	-176.24(17)
C(21)-C(16)-C(17)-C(18)	-0.3(3)
C(16)-C(17)-C(18)-C(19)	0.1(3)
C(17)-C(18)-C(19)-C(20)	0.2(3)
C(18)-C(19)-C(20)-C(21)	-0.2(3)
C(19)-C(20)-C(21)-C(16)	0.0(3)
C(19)-C(20)-C(21)-C(22)	176.83(18)
C(17)-C(16)-C(21)-C(20)	0.2(3)
N(3)-C(16)-C(21)-C(20)	176.96(15)
C(17)-C(16)-C(21)-C(22)	-177.36(16)
N(3)-C(16)-C(21)-C(22)	-0.66(18)
C(20)-C(21)-C(22)-C(23)	3.3(3)
C(16)-C(21)-C(22)-C(23)	-179.57(18)
C(20)-C(21)-C(22)-C(27)	-176.12(18)
C(16)-C(21)-C(22)-C(27)	1.01(18)
C(27)-C(22)-C(23)-C(24)	0.2(3)
C(21)-C(22)-C(23)-C(24)	-179.14(18)
C(22)-C(23)-C(24)-C(25)	-0.6(3)

C(23)-C(24)-C(25)-C(26)	0.2(3)
C(24)-C(25)-C(26)-C(27)	0.5(3)
C(25)-C(26)-C(27)-N(3)	-179.62(17)
C(25)-C(26)-C(27)-C(22)	-0.9(3)
C(23)-C(22)-C(27)-C(26)	0.5(3)
C(21)-C(22)-C(27)-C(26)	-179.95(15)
C(23)-C(22)-C(27)-N(3)	179.50(15)
C(21)-C(22)-C(27)-N(3)	-0.99(18)
N(2)-C(28)-C(29)-C(30)	-179.98(15)
C(28)-C(29)-C(30)-C(31)	-179.59(16)
C(29)-C(30)-C(31)-C(32)	173.08(16)
C(30)-C(31)-C(32)-C(33)	179.68(16)
C(31)-C(32)-C(33)-C(34)	170.94(16)
C(32)-C(33)-C(34)-C(35)	-172.47(17)
N(2)-C(1)-N(1)-C(3)	0.58(19)
C(2)-C(3)-N(1)-C(1)	-0.28(19)
C(4)-C(3)-N(1)-C(1)	-178.15(15)
N(1)-C(1)-N(2)-C(2)	-0.65(19)
N(1)-C(1)-N(2)-C(28)	-178.42(15)
C(3)-C(2)-N(2)-C(1)	0.44(18)
C(3)-C(2)-N(2)-C(28)	178.25(15)
C(29)-C(28)-N(2)-C(1)	-13.6(2)
C(29)-C(28)-N(2)-C(2)	169.08(16)
C(17)-C(16)-N(3)-C(27)	176.42(17)
C(21)-C(16)-N(3)-C(27)	0.05(18)
C(17)-C(16)-N(3)-C(13)	-4.2(3)
C(21)-C(16)-N(3)-C(13)	179.40(15)
C(26)-C(27)-N(3)-C(16)	179.44(17)
C(22)-C(27)-N(3)-C(16)	0.60(18)
C(26)-C(27)-N(3)-C(13)	0.1(3)
C(22)-C(27)-N(3)-C(13)	-178.73(15)
C(12)-C(13)-N(3)-C(16)	-47.5(2)
C(14)-C(13)-N(3)-C(16)	131.52(18)
C(12)-C(13)-N(3)-C(27)	131.67(18)
C(14)-C(13)-N(3)-C(27)	-49.3(2)

5. Single crystal X-ray diffraction full date set of **$ZnCl_2(ImL1)_2$** complex

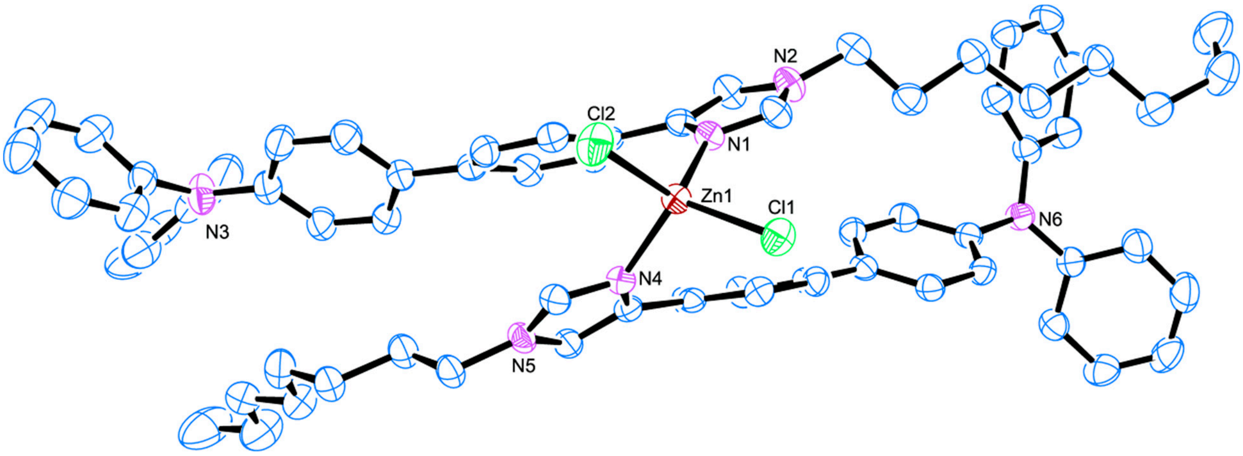

Figure 7 from the main text. Perspective view of **$ZnCl_2(ImL1)_2$** showing the atom-numbering scheme. Displacement ellipsoids are drawn at the 50% probability level and H atoms are shown as small spheres of arbitrary radii. H atoms on C atoms are omitted for clarity. Atom labels on C atoms are not shown.

Table S11. Atomic coordinates (x 104) and equivalent isotropic displacement parameters (Å2x 103) for **$ZnCl_2(ImL1)_2$** complex.

	x	y	z	U(eq
C(1)	8772(2)	7526(3)	2162(1)	35(1)
C(2)	7542(2)	7761(3)	2360(1)	39(1)
C(3)	8054(2)	6690(3)	2512(1)	31(1)
C(4)	7857(2)	5816(3)	2772(1)	31(1)
C(5)	6980(2)	5445(3)	2802(1)	34(1)
C(6)	6783(2)	4659(3)	3050(1)	39(1)
C(7)	7450(2)	4220(3)	3281(1)	35(1)
C(8)	8326(2)	4558(3)	3245(1)	38(1)
C(9)	8525(2)	5345(3)	2998(1)	35(1)
C(10)	7233(2)	3491(3)	3560(1)	39(1)
C(11)	6500(2)	2533(4)	3557(1)	43(1)
C(12)	6304(3)	1899(4)	3822(1)	46(1)
C(13)	6824(2)	2180(4)	4097(1)	45(1)
C(14)	7547(3)	3143(4)	4102(1)	51(1)
C(15)	7744(2)	3778(4)	3837(1)	47(1)
C(16)	5765(2)	1188(4)	4423(1)	46(1)

C(17)	5061(3)	2141(6)	4353(1)	71(1)
C(18)	4202(3)	1707(7)	4429(1)	77(1)
C(19)	4106(3)	410(6)	4568(1)	75(1)
C(20)	4772(3)	-534(6)	4631(1)	72(1)
C(21)	5605(2)	-158(5)	4562(1)	58(1)
C(22)	7346(2)	1310(4)	4614(1)	43(1)
C(23)	7257(3)	1827(5)	4900(1)	53(1)
C(24)	7930(3)	1606(6)	5136(1)	64(1)
C(25)	8692(3)	884(5)	5098(1)	62(1)
C(26)	8793(3)	354(5)	4814(1)	57(1)
C(27)	8118(2)	556(4)	4573(1)	51(1)
C(28)	7748(2)	9552(3)	1936(1)	41(1)
C(29)	7341(2)	9061(4)	1624(1)	42(1)
C(30)	7110(2)	10471(4)	1429(1)	42(1)
C(31)	6821(2)	10115(4)	1100(1)	43(1)
C(32)	6588(2)	11486(3)	902(1)	39(1)
C(33)	6248(3)	11023(4)	578(1)	50(1)
C(34)	6071(3)	12325(5)	356(1)	62(1)
C(35)	5328(3)	13285(6)	419(1)	75(1)
C(36)	9504(2)	2169(3)	2732(1)	31(1)
C(37)	8124(2)	1455(3)	2604(1)	32(1)
C(38)	8296(2)	2606(3)	2424(1)	28(1)
C(39)	7685(2)	3305(3)	2177(1)	27(1)
C(40)	6770(2)	3277(3)	2187(1)	31(1)
C(41)	6186(2)	3959(3)	1965(1)	33(1)
C(42)	6487(2)	4700(3)	1723(1)	30(1)
C(43)	7403(2)	4678(3)	1708(1)	30(1)
C(44)	7992(2)	3984(3)	1931(1)	30(1)
C(45)	5854(2)	5501(3)	1493(1)	30(1)
C(46)	5992(2)	5610(3)	1193(1)	32(1)
C(47)	5427(2)	6442(3)	984(1)	34(1)
C(48)	4693(2)	7164(3)	1072(1)	32(1)
C(49)	4530(2)	7026(3)	1369(1)	36(1)
C(50)	5107(2)	6221(3)	1576(1)	34(1)
C(51)	3995(2)	9590(3)	942(1)	35(1)
C(52)	4550(2)	10298(3)	1175(1)	37(1)
C(53)	4436(2)	11788(4)	1238(1)	40(1)
C(54)	3766(2)	12607(4)	1072(1)	47(1)
C(55)	3194(3)	11898(4)	851(1)	54(1)
C(56)	3292(2)	10403(4)	786(1)	48(1)
C(57)	3910(2)	7568(3)	561(1)	34(1)

C(58)	4005(2)	8452(4)	314(1)	48(1)
C(59)	3784(3)	7891(5)	23(1)	61(1)
C(60)	3465(3)	6465(5)	-24(1)	56(1)
C(61)	3375(2)	5584(4)	220(1)	51(1)
C(62)	3595(2)	6122(4)	513(1)	40(1)
C(63)	9048(2)	-9(4)	3024(1)	39(1)
C(64)	8384(2)	0(5)	3246(1)	43(1)
C(65)	8636(3)	-1214(4)	3484(1)	49(1)
C(66)	7998(3)	-1340(5)	3716(1)	57(1)
C(67)	7077(3)	-1890(4)	3598(1)	55(1)
C(68)	6532(3)	-2312(4)	3847(1)	58(1)
C(69)	5616(3)	-2881(6)	3735(1)	74(1)
C(70)	5093(4)	-3360(7)	3988(1)	98(2)
N(1)	8840(2)	6552(3)	2386(1)	30(1)
N(2)	8010(2)	8298(3)	2141(1)	38(1)
N(3)	6646(2)	1500(4)	4367(1)	50(1)
N(4)	9175(2)	3050(2)	2506(1)	27(1)
N(5)	8901(2)	1193(3)	2798(1)	33(1)
N(6)	4135(2)	8081(3)	865(1)	36(1)
Zn(1)	9812(1)	4974(1)	2426(1)	28(1)
Cl(1)	10389(1)	4960(1)	1993(1)	39(1)
Cl(2)	10890(1)	5275(1)	2822(1)	47(1)

Table S12. Bond lengths [Å] and angles [°] for **$ZnCl_2(ImL1)_2$** complex.

Bond lengths [Å]	
C(1)-N(1)	1.318(4)
C(1)-N(2)	1.336(4)
C(1)-H(1)	0.95
C(2)-C(3)	1.353(4)
C(2)-N(2)	1.371(4)
C(2)-H(2)	0.95
C(3)-N(1)	1.389(4)
C(3)-C(4)	1.467(4)
C(4)-C(9)	1.387(4)
C(4)-C(5)	1.393(4)
C(5)-C(6)	1.378(4)
C(5)-H(5)	0.95
C(6)-C(7)	1.393(4)
C(6)-H(6)	0.95
C(7)-C(8)	1.390(4)

C(7)-C(10)	1.481(4)
C(8)-C(9)	1.378(4)
C(8)-H(8)	0.95
C(9)-H(9)	0.95
C(10)-C(15)	1.388(5)
C(10)-C(11)	1.400(5)
C(11)-C(12)	1.381(5)
C(11)-H(11)	0.95
C(12)-C(13)	1.384(5)
C(12)-H(12)	0.95
C(13)-C(14)	1.390(5)
C(13)-N(3)	1.409(4)
C(14)-C(15)	1.382(5)
C(14)-H(14)	0.95
C(15)-H(15)	0.95
C(16)-C(17)	1.365(6)
C(16)-C(21)	1.393(6)
C(16)-N(3)	1.417(4)
C(17)-C(18)	1.442(7)
C(17)-H(17)	0.95
C(18)-C(19)	1.333(7)
C(18)-H(18)	0.95
C(19)-C(20)	1.314(7)
C(19)-H(19)	0.95
C(20)-C(21)	1.380(6)
C(20)-H(20)	0.95
C(21)-H(21)	0.95
C(22)-C(23)	1.381(5)
C(22)-C(27)	1.383(5)
C(22)-N(3)	1.425(4)
C(23)-C(24)	1.372(5)
C(23)-H(23)	0.95
C(24)-C(25)	1.354(6)
C(24)-H(24)	0.95
C(25)-C(26)	1.382(5)
C(25)-H(25)	0.95
C(26)-C(27)	1.385(5)
C(26)-H(26)	0.95
C(27)-H(27)	0.95
C(28)-N(2)	1.466(4)
C(28)-C(29)	1.508(5)

C(28)-H(28A)	0.99
C(28)-H(28B)	0.99
C(29)-C(30)	1.543(4)
C(29)-H(29A)	0.99
C(29)-H(29B)	0.99
C(30)-C(31)	1.507(4)
C(30)-H(30A)	0.99
C(30)-H(30B)	0.99
C(31)-C(32)	1.524(4)
C(31)-H(31A)	0.99
C(31)-H(31B)	0.99
C(32)-C(33)	1.520(4)
C(32)-H(32A)	0.99
C(32)-H(32B)	0.99
C(33)-C(34)	1.529(5)
C(33)-H(33A)	0.99
C(33)-H(33B)	0.99
C(34)-C(35)	1.472(6)
C(34)-H(34A)	0.99
C(34)-H(34B)	0.99
C(35)-H(35A)	0.98
C(35)-H(35B)	0.98
C(35)-H(35C)	0.98
C(36)-N(4)	1.319(3)
C(36)-N(5)	1.327(4)
C(36)-H(36)	0.95
C(37)-C(38)	1.355(4)
C(37)-N(5)	1.377(4)
C(37)-H(37)	0.95
C(38)-N(4)	1.386(3)
C(38)-C(39)	1.473(4)
C(39)-C(40)	1.391(4)
C(39)-C(44)	1.394(4)
C(40)-C(41)	1.375(4)
C(40)-H(40)	0.95
C(41)-C(42)	1.397(4)
C(41)-H(41)	0.95
C(42)-C(43)	1.397(4)
C(42)-C(45)	1.483(4)
C(43)-C(44)	1.383(4)
C(43)-H(43)	0.95

C(44)-H(44)	0.95
C(45)-C(46)	1.389(4)
C(45)-C(50)	1.395(4)
C(46)-C(47)	1.389(4)
C(46)-H(46)	0.95
C(47)-C(48)	1.388(4)
C(47)-H(47)	0.95
C(48)-C(49)	1.387(4)
C(48)-N(6)	1.422(4)
C(49)-C(50)	1.381(4)
C(49)-H(49)	0.95
C(50)-H(50)	0.95
C(51)-C(56)	1.391(4)
C(51)-C(52)	1.393(4)
C(51)-N(6)	1.417(4)
C(52)-C(53)	1.379(4)
C(52)-H(52)	0.95
C(53)-C(54)	1.380(5)
C(53)-H(53)	0.95
C(54)-C(55)	1.372(5)
C(54)-H(54)	0.95
C(55)-C(56)	1.381(5)
C(55)-H(55)	0.95
C(56)-H(56)	0.95
C(57)-C(58)	1.383(4)
C(57)-C(62)	1.385(4)
C(57)-N(6)	1.424(4)
C(58)-C(59)	1.386(5)
C(58)-H(58)	0.95
C(59)-C(60)	1.370(6)
C(59)-H(59)	0.95
C(60)-C(61)	1.367(5)
C(60)-H(60)	0.95
C(61)-C(62)	1.386(4)
C(61)-H(61)	0.95
C(62)-H(62)	0.95
C(63)-N(5)	1.470(4)
C(63)-C(64)	1.506(4)
C(63)-H(63A)	0.99
C(63)-H(63B)	0.99
C(64)-C(65)	1.528(4)

C(64)-H(64A)	0.99
C(64)-H(64B)	0.99
C(65)-C(66)	1.517(5)
C(65)-H(65A)	0.99
C(65)-H(65B)	0.99
C(66)-C(67)	1.500(5)
C(66)-H(66A)	0.99
C(66)-H(66B)	0.99
C(67)-C(68)	1.524(6)
C(67)-H(67A)	0.99
C(67)-H(67B)	0.99
C(68)-C(69)	1.495(6)
C(68)-H(68A)	0.99
C(68)-H(68B)	0.99
C(69)-C(70)	1.528(7)
C(69)-H(69A)	0.99
C(69)-H(69B)	0.99
C(70)-H(70A)	0.98
C(70)-H(70B)	0.98
C(70)-H(70C)	0.98
N(1)-Zn(1)	2.029(2)
N(4)-Zn(1)	2.030(2)
Zn(1)-Cl(1)	2.2274(8)
Zn(1)-Cl(2)	2.2453(8)
Bond angles [°]	
N(1)-C(1)-N(2)	111.6(3)
N(1)-C(1)-H(1)	124.2
N(2)-C(1)-H(1)	124.2
C(3)-C(2)-N(2)	106.7(3)
C(3)-C(2)-H(2)	126.6
N(2)-C(2)-H(2)	126.6
C(2)-C(3)-N(1)	108.8(3)
C(2)-C(3)-C(4)	127.5(3)
N(1)-C(3)-C(4)	123.7(2)
C(9)-C(4)-C(5)	117.7(3)
C(9)-C(4)-C(3)	121.9(3)
C(5)-C(4)-C(3)	120.5(3)
C(6)-C(5)-C(4)	121.1(3)
C(6)-C(5)-H(5)	119.5
C(4)-C(5)-H(5)	119.5
C(5)-C(6)-C(7)	121.4(3)

C(5)-C(6)-H(6)	119.3
C(7)-C(6)-H(6)	119.3
C(8)-C(7)-C(6)	117.1(3)
C(8)-C(7)-C(10)	121.4(3)
C(6)-C(7)-C(10)	121.4(3)
C(9)-C(8)-C(7)	121.6(3)
C(9)-C(8)-H(8)	119.2
C(7)-C(8)-H(8)	119.2
C(8)-C(9)-C(4)	121.1(3)
C(8)-C(9)-H(9)	119.5
C(4)-C(9)-H(9)	119.5
C(15)-C(10)-C(11)	117.4(3)
C(15)-C(10)-C(7)	120.3(3)
C(11)-C(10)-C(7)	122.3(3)
C(12)-C(11)-C(10)	120.8(3)
C(12)-C(11)-H(11)	119.6
C(10)-C(11)-H(11)	119.6
C(11)-C(12)-C(13)	121.3(3)
C(11)-C(12)-H(12)	119.3
C(13)-C(12)-H(12)	119.3
C(12)-C(13)-C(14)	118.3(3)
C(12)-C(13)-N(3)	121.7(3)
C(14)-C(13)-N(3)	120.0(3)
C(15)-C(14)-C(13)	120.4(3)
C(15)-C(14)-H(14)	119.8
C(13)-C(14)-H(14)	119.8
C(14)-C(15)-C(10)	121.8(3)
C(14)-C(15)-H(15)	119.1
C(10)-C(15)-H(15)	119.1
C(17)-C(16)-C(21)	117.5(4)
C(17)-C(16)-N(3)	124.0(4)
C(21)-C(16)-N(3)	118.4(3)
C(16)-C(17)-C(18)	118.6(5)
C(16)-C(17)-H(17)	120.7
C(18)-C(17)-H(17)	120.7
C(19)-C(18)-C(17)	120.2(5)
C(19)-C(18)-H(18)	119.9
C(17)-C(18)-H(18)	119.9
C(20)-C(19)-C(18)	122.0(5)
C(20)-C(19)-H(19)	119
C(18)-C(19)-H(19)	119

C(19)-C(20)-C(21)	119.4(5)
C(19)-C(20)-H(20)	120.3
C(21)-C(20)-H(20)	120.3
C(20)-C(21)-C(16)	122.2(4)
C(20)-C(21)-H(21)	118.9
C(16)-C(21)-H(21)	118.9
C(23)-C(22)-C(27)	118.7(3)
C(23)-C(22)-N(3)	121.0(3)
C(27)-C(22)-N(3)	120.3(3)
C(24)-C(23)-C(22)	120.2(4)
C(24)-C(23)-H(23)	119.9
C(22)-C(23)-H(23)	119.9
C(25)-C(24)-C(23)	121.6(3)
C(25)-C(24)-H(24)	119.2
C(23)-C(24)-H(24)	119.2
C(24)-C(25)-C(26)	119.1(4)
C(24)-C(25)-H(25)	120.4
C(26)-C(25)-H(25)	120.4
C(25)-C(26)-C(27)	120.1(4)
C(25)-C(26)-H(26)	120
C(27)-C(26)-H(26)	120
C(22)-C(27)-C(26)	120.4(3)
C(22)-C(27)-H(27)	119.8
C(26)-C(27)-H(27)	119.8
N(2)-C(28)-C(29)	113.1(3)
N(2)-C(28)-H(28A)	109
C(29)-C(28)-H(28A)	109
N(2)-C(28)-H(28B)	109
C(29)-C(28)-H(28B)	109
H(28A)-C(28)-H(28B)	107.8
C(28)-C(29)-C(30)	108.2(3)
C(28)-C(29)-H(29A)	110.1
C(30)-C(29)-H(29A)	110.1
C(28)-C(29)-H(29B)	110.1
C(30)-C(29)-H(29B)	110.1
H(29A)-C(29)-H(29B)	108.4
C(31)-C(30)-C(29)	112.8(3)
C(31)-C(30)-H(30A)	109
C(29)-C(30)-H(30A)	109
C(31)-C(30)-H(30B)	109
C(29)-C(30)-H(30B)	109

H(30A)-C(30)-H(30B)	107.8
C(30)-C(31)-C(32)	114.1(3)
C(30)-C(31)-H(31A)	108.7
C(32)-C(31)-H(31A)	108.7
C(30)-C(31)-H(31B)	108.7
C(32)-C(31)-H(31B)	108.7
H(31A)-C(31)-H(31B)	107.6
C(33)-C(32)-C(31)	110.6(3)
C(33)-C(32)-H(32A)	109.5
C(31)-C(32)-H(32A)	109.5
C(33)-C(32)-H(32B)	109.5
C(31)-C(32)-H(32B)	109.5
H(32A)-C(32)-H(32B)	108.1
C(32)-C(33)-C(34)	114.4(3)
C(32)-C(33)-H(33A)	108.7
C(34)-C(33)-H(33A)	108.7
C(32)-C(33)-H(33B)	108.7
C(34)-C(33)-H(33B)	108.7
H(33A)-C(33)-H(33B)	107.6
C(35)-C(34)-C(33)	113.0(3)
C(35)-C(34)-H(34A)	109
C(33)-C(34)-H(34A)	109
C(35)-C(34)-H(34B)	109
C(33)-C(34)-H(34B)	109
H(34A)-C(34)-H(34B)	107.8
C(34)-C(35)-H(35A)	109.5
C(34)-C(35)-H(35B)	109.5
H(35A)-C(35)-H(35B)	109.5
C(34)-C(35)-H(35C)	109.5
H(35A)-C(35)-H(35C)	109.5
H(35B)-C(35)-H(35C)	109.5
N(4)-C(36)-N(5)	111.6(2)
N(4)-C(36)-H(36)	124.2
N(5)-C(36)-H(36)	124.2
C(38)-C(37)-N(5)	106.5(2)
C(38)-C(37)-H(37)	126.7
N(5)-C(37)-H(37)	126.7
C(37)-C(38)-N(4)	108.6(2)
C(37)-C(38)-C(39)	127.8(3)
N(4)-C(38)-C(39)	123.6(2)
C(40)-C(39)-C(44)	118.5(2)

C(40)-C(39)-C(38)	119.4(2)
C(44)-C(39)-C(38)	122.1(2)
C(41)-C(40)-C(39)	120.6(3)
C(41)-C(40)-H(40)	119.7
C(39)-C(40)-H(40)	119.7
C(40)-C(41)-C(42)	121.5(3)
C(40)-C(41)-H(41)	119.2
C(42)-C(41)-H(41)	119.2
C(41)-C(42)-C(43)	117.6(2)
C(41)-C(42)-C(45)	120.9(2)
C(43)-C(42)-C(45)	121.5(2)
C(44)-C(43)-C(42)	121.0(2)
C(44)-C(43)-H(43)	119.5
C(42)-C(43)-H(43)	119.5
C(43)-C(44)-C(39)	120.6(2)
C(43)-C(44)-H(44)	119.7
C(39)-C(44)-H(44)	119.7
C(46)-C(45)-C(50)	117.5(2)
C(46)-C(45)-C(42)	121.9(3)
C(50)-C(45)-C(42)	120.6(2)
C(45)-C(46)-C(47)	121.5(3)
C(45)-C(46)-H(46)	119.2
C(47)-C(46)-H(46)	119.2
C(48)-C(47)-C(46)	120.0(3)
C(48)-C(47)-H(47)	120
C(46)-C(47)-H(47)	120
C(49)-C(48)-C(47)	119.1(3)
C(49)-C(48)-N(6)	120.3(3)
C(47)-C(48)-N(6)	120.6(3)
C(50)-C(49)-C(48)	120.4(3)
C(50)-C(49)-H(49)	119.8
C(48)-C(49)-H(49)	119.8
C(49)-C(50)-C(45)	121.5(3)
C(49)-C(50)-H(50)	119.3
C(45)-C(50)-H(50)	119.3
C(56)-C(51)-C(52)	118.2(3)
C(56)-C(51)-N(6)	120.5(3)
C(52)-C(51)-N(6)	121.3(3)
C(53)-C(52)-C(51)	120.9(3)
C(53)-C(52)-H(52)	119.5
C(51)-C(52)-H(52)	119.5

C(52)-C(53)-C(54)	120.4(3)
C(52)-C(53)-H(53)	119.8
C(54)-C(53)-H(53)	119.8
C(55)-C(54)-C(53)	118.8(3)
C(55)-C(54)-H(54)	120.6
C(53)-C(54)-H(54)	120.6
C(54)-C(55)-C(56)	121.6(3)
C(54)-C(55)-H(55)	119.2
C(56)-C(55)-H(55)	119.2
C(55)-C(56)-C(51)	119.9(3)
C(55)-C(56)-H(56)	120
C(51)-C(56)-H(56)	120
C(58)-C(57)-C(62)	118.8(3)
C(58)-C(57)-N(6)	122.5(3)
C(62)-C(57)-N(6)	118.7(3)
C(57)-C(58)-C(59)	120.1(3)
C(57)-C(58)-H(58)	120
C(59)-C(58)-H(58)	120
C(60)-C(59)-C(58)	120.9(3)
C(60)-C(59)-H(59)	119.5
C(58)-C(59)-H(59)	119.5
C(61)-C(60)-C(59)	119.2(3)
C(61)-C(60)-H(60)	120.4
C(59)-C(60)-H(60)	120.4
C(60)-C(61)-C(62)	120.8(3)
C(60)-C(61)-H(61)	119.6
C(62)-C(61)-H(61)	119.6
C(57)-C(62)-C(61)	120.2(3)
C(57)-C(62)-H(62)	119.9
C(61)-C(62)-H(62)	119.9
N(5)-C(63)-C(64)	113.3(3)
N(5)-C(63)-H(63A)	108.9
C(64)-C(63)-H(63A)	108.9
N(5)-C(63)-H(63B)	108.9
C(64)-C(63)-H(63B)	108.9
H(63A)-C(63)-H(63B)	107.7
C(63)-C(64)-C(65)	109.1(3)
C(63)-C(64)-H(64A)	109.9
C(65)-C(64)-H(64A)	109.9
C(63)-C(64)-H(64B)	109.9
C(65)-C(64)-H(64B)	109.9

H(64A)-C(64)-H(64B)	108.3
C(66)-C(65)-C(64)	113.9(3)
C(66)-C(65)-H(65A)	108.8
C(64)-C(65)-H(65A)	108.8
C(66)-C(65)-H(65B)	108.8
C(64)-C(65)-H(65B)	108.8
H(65A)-C(65)-H(65B)	107.7
C(67)-C(66)-C(65)	115.8(3)
C(67)-C(66)-H(66A)	108.3
C(65)-C(66)-H(66A)	108.3
C(67)-C(66)-H(66B)	108.3
C(65)-C(66)-H(66B)	108.3
H(66A)-C(66)-H(66B)	107.4
C(66)-C(67)-C(68)	113.4(3)
C(66)-C(67)-H(67A)	108.9
C(68)-C(67)-H(67A)	108.9
C(66)-C(67)-H(67B)	108.9
C(68)-C(67)-H(67B)	108.9
H(67A)-C(67)-H(67B)	107.7
C(69)-C(68)-C(67)	114.3(3)
C(69)-C(68)-H(68A)	108.7
C(67)-C(68)-H(68A)	108.7
C(69)-C(68)-H(68B)	108.7
C(67)-C(68)-H(68B)	108.7
H(68A)-C(68)-H(68B)	107.6
C(68)-C(69)-C(70)	113.6(4)
C(68)-C(69)-H(69A)	108.8
C(70)-C(69)-H(69A)	108.8
C(68)-C(69)-H(69B)	108.8
C(70)-C(69)-H(69B)	108.8
H(69A)-C(69)-H(69B)	107.7
C(69)-C(70)-H(70A)	109.5
C(69)-C(70)-H(70B)	109.5
H(70A)-C(70)-H(70B)	109.5
C(69)-C(70)-H(70C)	109.5
H(70A)-C(70)-H(70C)	109.5
H(70B)-C(70)-H(70C)	109.5
C(1)-N(1)-C(3)	105.6(2)
C(1)-N(1)-Zn(1)	120.21(19)
C(3)-N(1)-Zn(1)	132.68(19)
C(1)-N(2)-C(2)	107.2(2)

C(1)-N(2)-C(28)	126.5(3)
C(2)-N(2)-C(28)	126.2(3)
C(13)-N(3)-C(16)	122.2(3)
C(13)-N(3)-C(22)	120.0(3)
C(16)-N(3)-C(22)	117.1(3)
C(36)-N(4)-C(38)	105.9(2)
C(36)-N(4)-Zn(1)	120.34(19)
C(38)-N(4)-Zn(1)	131.11(18)
C(36)-N(5)-C(37)	107.4(2)
C(36)-N(5)-C(63)	126.4(2)
C(37)-N(5)-C(63)	126.1(2)
C(51)-N(6)-C(48)	119.1(2)
C(51)-N(6)-C(57)	121.0(2)
C(48)-N(6)-C(57)	118.2(2)
N(1)-Zn(1)-N(4)	104.43(9)
N(1)-Zn(1)-Cl(1)	107.24(7)
N(4)-Zn(1)-Cl(1)	113.76(7)
N(1)-Zn(1)-Cl(2)	114.91(7)
N(4)-Zn(1)-Cl(2)	105.77(7)
Cl(1)-Zn(1)-Cl(2)	110.74(3)

TableS13. Anisotropic displacement parameters (Å2x 103) for **$ZnCl_2(ImL1)_2$** complex.

	U^{11}	U^{22}	U^{33}	U^{23}	U^{13}	U^{12}
C(1)	31(1)	32(2)	43(2)	6(1)	11(1)	1(1)
C(2)	32(2)	38(2)	48(2)	7(1)	14(1)	6(1)
C(3)	30(1)	28(1)	35(1)	-2(1)	7(1)	0(1)
C(4)	35(2)	27(1)	32(1)	-5(1)	10(1)	5(1)
C(5)	33(1)	38(2)	34(1)	1(1)	7(1)	5(1)
C(6)	36(1)	41(2)	41(2)	2(1)	11(1)	0(1)
C(7)	45(2)	30(1)	33(1)	-3(1)	10(1)	4(1)
C(8)	44(2)	37(2)	33(1)	1(1)	4(1)	6(1)
C(9)	31(1)	35(2)	38(1)	-3(1)	4(1)	2(1)
C(10)	49(2)	34(2)	34(2)	4(1)	8(1)	1(1)
C(11)	53(2)	40(2)	34(2)	1(1)	-1(1)	-5(1)
C(12)	55(2)	44(2)	39(2)	5(1)	3(1)	-11(2)
C(13)	56(2)	43(2)	36(2)	7(1)	5(1)	-6(2)
C(14)	64(2)	56(2)	32(2)	2(1)	2(2)	-15(2)
C(15)	53(2)	46(2)	41(2)	3(1)	7(1)	-12(2)
C(16)	44(2)	60(2)	34(2)	-7(2)	4(1)	0(2)

C(17)	81(3)	78(3)	53(2)	-2(2)	5(2)	18(2)
C(18)	56(3)	120(4)	53(2)	-16(3)	2(2)	28(3)
C(19)	67(3)	103(4)	55(2)	-33(3)	13(2)	-16(3)
C(20)	77(3)	74(3)	71(3)	-22(2)	30(2)	-29(2)
C(21)	56(2)	64(2)	57(2)	-5(2)	16(2)	-11(2)
C(22)	49(2)	47(2)	35(2)	8(1)	7(1)	-14(2)
C(23)	50(2)	66(2)	47(2)	-8(2)	15(2)	-14(2)
C(24)	56(2)	95(3)	40(2)	-16(2)	8(2)	-25(2)
C(25)	51(2)	86(3)	45(2)	2(2)	-3(2)	-22(2)
C(26)	47(2)	69(3)	54(2)	-2(2)	2(2)	-3(2)
C(27)	53(2)	63(2)	38(2)	-2(2)	5(2)	-5(2)
C(28)	41(2)	33(2)	50(2)	5(1)	5(1)	-1(1)
C(29)	43(2)	36(2)	49(2)	-1(1)	15(1)	4(1)
C(30)	40(2)	40(2)	45(2)	7(1)	7(1)	3(1)
C(31)	45(2)	40(2)	46(2)	7(2)	14(1)	9(2)
C(32)	41(2)	36(2)	39(2)	4(1)	7(1)	3(1)
C(33)	51(2)	50(2)	47(2)	-6(2)	3(2)	3(2)
C(34)	63(2)	82(3)	38(2)	3(2)	-2(2)	9(2)
C(35)	96(3)	79(3)	44(2)	-4(2)	-8(2)	28(3)
C(36)	26(1)	31(1)	35(1)	1(1)	-2(1)	3(1)
C(37)	32(1)	31(1)	32(1)	1(1)	-2(1)	-3(1)
C(38)	28(1)	28(1)	28(1)	-4(1)	1(1)	1(1)
C(39)	28(1)	24(1)	28(1)	-3(1)	-3(1)	-1(1)
C(40)	30(1)	28(1)	33(1)	6(1)	0(1)	-7(1)
C(41)	22(1)	36(2)	40(2)	6(1)	-2(1)	-5(1)
C(42)	29(1)	26(2)	33(1)	0(1)	-2(1)	-2(1)
C(43)	30(1)	33(2)	27(1)	2(1)	0(1)	-3(1)
C(44)	23(1)	35(1)	32(1)	-2(1)	1(1)	0(1)
C(45)	28(1)	26(1)	32(1)	1(1)	-4(1)	-3(1)
C(46)	31(1)	29(1)	34(1)	0(1)	-2(1)	3(1)
C(47)	38(2)	34(2)	27(1)	-1(1)	-1(1)	3(1)
C(48)	33(1)	27(1)	33(1)	-1(1)	-2(1)	1(1)
C(49)	33(2)	35(2)	39(2)	1(1)	4(1)	4(1)
C(50)	35(2)	35(2)	31(1)	2(1)	3(1)	-1(1)
C(51)	34(1)	33(2)	38(1)	3(1)	6(1)	5(1)
C(52)	35(1)	36(2)	39(1)	1(1)	2(1)	7(1)
C(53)	42(2)	39(2)	42(2)	-5(1)	9(1)	0(1)
C(54)	56(2)	33(2)	54(2)	0(1)	14(2)	10(1)
C(55)	56(2)	43(2)	62(2)	4(2)	-1(2)	23(2)
C(56)	43(2)	46(2)	51(2)	-1(1)	-7(1)	13(1)
C(57)	29(1)	37(2)	35(1)	3(1)	-3(1)	6(1)

C(58)	54(2)	46(2)	42(2)	6(2)	1(2)	-4(2)
C(59)	73(3)	69(3)	38(2)	12(2)	1(2)	-2(2)
C(60)	61(2)	69(2)	33(2)	-8(2)	-9(2)	7(2)
C(61)	48(2)	50(2)	48(2)	-9(2)	-9(2)	-4(2)
C(62)	38(2)	41(2)	41(2)	4(1)	0(1)	-2(1)
C(63)	45(2)	32(1)	37(1)	8(2)	-2(1)	4(2)
C(64)	50(2)	38(1)	40(1)	7(2)	0(1)	0(2)
C(65)	65(2)	40(2)	41(2)	7(1)	-2(2)	-1(2)
C(66)	73(3)	57(2)	39(2)	7(2)	2(2)	-5(2)
C(67)	69(2)	51(2)	42(2)	-3(2)	2(2)	1(2)
C(68)	81(3)	42(2)	50(2)	0(2)	6(2)	-5(2)
C(69)	74(3)	83(3)	65(3)	-13(2)	10(2)	-12(2)
C(70)	105(4)	104(4)	90(4)	-36(3)	31(3)	-49(3)
N(1)	26(1)	28(1)	35(1)	1(1)	5(1)	1(1)
N(2)	34(1)	36(1)	46(1)	10(1)	7(1)	4(1)
N(3)	47(2)	63(2)	38(1)	15(1)	4(1)	-8(1)
N(4)	23(1)	28(1)	31(1)	-1(1)	2(1)	1(1)
N(5)	34(1)	29(1)	34(1)	6(1)	-1(1)	3(1)
N(6)	40(1)	32(1)	34(1)	1(1)	-7(1)	9(1)
Zn(1)	21(1)	30(1)	31(1)	0(1)	2(1)	0(1)
Cl(1)	33(1)	50(1)	38(1)	-1(1)	12(1)	-1(1)
Cl(2)	30(1)	67(1)	40(1)	1(1)	-7(1)	-12(1)

Table S14. Hydrogen coordinates (x 104) and isotropic displacement parameters (Å2x 103) for **ZnCl$_2$(ImL1)$_2$** complex.

	x	y	z	U(eq)
H(1)	9212	7662	2032	42
H(2)	6969	8080	2397	46
H(5)	6509	5737	2649	41
H(6)	6179	4411	3064	46
H(8)	8798	4239	3395	46
H(9)	9131	5569	2982	42
H(11)	6134	2317	3372	52
H(12)	5801	1257	3816	55
H(14)	7908	3367	4289	61
H(15)	8243	4429	3845	56
H(17)	5136	3071	4257	85
H(18)	3701	2348	4380	92
H(19)	3540	160	4622	89
H(20)	4681	-1467	4723	86
H(21)	6085	-840	4612	70

H(23)	6728	2337	4933	64
H(24)	7859	1969	5331	76
H(25)	9152	744	5264	74
H(26)	9326	-150	4784	69
H(27)	8186	176	4379	62
H(28A)	8281	10172	1920	49
H(28B)	7313	10182	2024	49
H(29A)	6794	8467	1635	50
H(29B)	7769	8430	1532	50
H(30A)	6624	11020	1509	50
H(30B)	7639	11132	1448	50
H(31A)	6293	9451	1083	51
H(31B)	7307	9561	1022	51
H(32A)	6124	12078	984	46
H(32B)	7125	12122	904	46
H(33A)	5688	10450	577	60
H(33B)	6692	10348	506	60
H(34A)	6619	12938	366	74
H(34B)	5934	11923	148	74
H(35A)	4776	12697	399	112
H(35B)	5255	14113	273	112
H(35C)	5458	13684	625	112
H(36)	10099	2226	2834	38
H(37)	7575	932	2598	39
H(40)	6546	2784	2349	37
H(41)	5564	3925	1975	40
H(43)	7625	5147	1543	36
H(44)	8612	3970	1915	36
H(46)	6485	5104	1129	38
H(47)	5542	6518	781	40
H(49)	4019	7488	1430	43
H(50)	4993	6157	1780	41
H(52)	5014	9747	1291	45
H(53)	4821	12253	1398	49
H(54)	3703	13644	1109	56
H(55)	2719	12448	740	65
H(56)	2880	9931	634	57
H(58)	4221	9446	343	58
H(59)	3856	8503	-146	73
H(60)	3308	6093	-224	67
H(61)	3160	4591	189	61

H(62)	3530	5497	680	48
H(63A)	9658	91	3138	47
H(63B)	9015	-983	2918	47
H(64A)	7776	-189	3138	52
H(64B)	8386	989	3346	52
H(65A)	8658	-2188	3380	59
H(65B)	9243	-1003	3591	59
H(66A)	8264	-2022	3878	68
H(66B)	7944	-344	3808	68
H(67A)	7127	-2774	3468	66
H(67B)	6756	-1102	3470	66
H(68A)	6860	-3089	3976	70
H(68B)	6479	-1423	3976	70
H(69A)	5665	-3745	3600	88
H(69B)	5277	-2089	3614	88
H(70A)	5378	-4238	4091	147
H(70B)	4479	-3609	3901	147
H(70C)	5087	-2541	4133	147

Table S15. Torsion angles [°] for **$ZnCl_2(ImL1)_2$** complex.

N(2)-C(2)-C(3)-N(1)	-0.4(3)
N(2)-C(2)-C(3)-C(4)	177.9(3)
C(2)-C(3)-C(4)-C(9)	-146.9(3)
N(1)-C(3)-C(4)-C(9)	31.3(4)
C(2)-C(3)-C(4)-C(5)	32.5(4)
N(1)-C(3)-C(4)-C(5)	-149.4(3)
C(9)-C(4)-C(5)-C(6)	1.1(4)
C(3)-C(4)-C(5)-C(6)	-178.2(3)
C(4)-C(5)-C(6)-C(7)	0.7(4)
C(5)-C(6)-C(7)-C(8)	-2.4(4)
C(5)-C(6)-C(7)-C(10)	174.7(3)
C(6)-C(7)-C(8)-C(9)	2.5(4)
C(10)-C(7)-C(8)-C(9)	-174.6(3)
C(7)-C(8)-C(9)-C(4)	-0.8(4)
C(5)-C(4)-C(9)-C(8)	-1.0(4)
C(3)-C(4)-C(9)-C(8)	178.3(3)
C(8)-C(7)-C(10)-C(15)	32.6(5)
C(6)-C(7)-C(10)-C(15)	-144.4(3)
C(8)-C(7)-C(10)-C(11)	-149.3(3)
C(6)-C(7)-C(10)-C(11)	33.7(5)

C(15)-C(10)-C(11)-C(12)	-0.3(5)
C(7)-C(10)-C(11)-C(12)	-178.5(3)
C(10)-C(11)-C(12)-C(13)	-0.4(6)
C(11)-C(12)-C(13)-C(14)	1.1(6)
C(11)-C(12)-C(13)-N(3)	-178.0(3)
C(12)-C(13)-C(14)-C(15)	-1.1(6)
N(3)-C(13)-C(14)-C(15)	178.0(4)
C(13)-C(14)-C(15)-C(10)	0.4(6)
C(11)-C(10)-C(15)-C(14)	0.3(5)
C(7)-C(10)-C(15)-C(14)	178.5(3)
C(21)-C(16)-C(17)-C(18)	0.2(6)
N(3)-C(16)-C(17)-C(18)	-178.3(3)
C(16)-C(17)-C(18)-C(19)	1.0(6)
C(17)-C(18)-C(19)-C(20)	-2.3(7)
C(18)-C(19)-C(20)-C(21)	2.3(7)
C(19)-C(20)-C(21)-C(16)	-1.0(6)
C(17)-C(16)-C(21)-C(20)	-0.2(5)
N(3)-C(16)-C(21)-C(20)	178.4(3)
C(27)-C(22)-C(23)-C(24)	0.7(6)
N(3)-C(22)-C(23)-C(24)	178.6(4)
C(22)-C(23)-C(24)-C(25)	0.0(6)
C(23)-C(24)-C(25)-C(26)	-0.2(7)
C(24)-C(25)-C(26)-C(27)	-0.3(6)
C(23)-C(22)-C(27)-C(26)	-1.2(5)
N(3)-C(22)-C(27)-C(26)	-179.1(3)
C(25)-C(26)-C(27)-C(22)	1.0(6)
N(2)-C(28)-C(29)-C(30)	-179.1(3)
C(28)-C(29)-C(30)-C(31)	171.7(3)
C(29)-C(30)-C(31)-C(32)	-179.9(3)
C(30)-C(31)-C(32)-C(33)	-176.4(3)
C(31)-C(32)-C(33)-C(34)	-175.1(3)
C(32)-C(33)-C(34)-C(35)	-66.4(5)
N(5)-C(37)-C(38)-N(4)	0.1(3)
N(5)-C(37)-C(38)-C(39)	179.6(3)
C(37)-C(38)-C(39)-C(40)	27.7(4)
N(4)-C(38)-C(39)-C(40)	-152.9(3)
C(37)-C(38)-C(39)-C(44)	-151.6(3)
N(4)-C(38)-C(39)-C(44)	27.8(4)
C(44)-C(39)-C(40)-C(41)	-2.6(4)
C(38)-C(39)-C(40)-C(41)	178.1(3)
C(39)-C(40)-C(41)-C(42)	-0.2(4)

C(40)-C(41)-C(42)-C(43)	2.4(4)
C(40)-C(41)-C(42)-C(45)	-176.6(3)
C(41)-C(42)-C(43)-C(44)	-1.8(4)
C(45)-C(42)-C(43)-C(44)	177.1(2)
C(42)-C(43)-C(44)-C(39)	-0.9(4)
C(40)-C(39)-C(44)-C(43)	3.1(4)
C(38)-C(39)-C(44)-C(43)	-177.6(2)
C(41)-C(42)-C(45)-C(46)	-148.0(3)
C(43)-C(42)-C(45)-C(46)	33.1(4)
C(41)-C(42)-C(45)-C(50)	34.2(4)
C(43)-C(42)-C(45)-C(50)	-144.7(3)
C(50)-C(45)-C(46)-C(47)	2.0(4)
C(42)-C(45)-C(46)-C(47)	-175.8(3)
C(45)-C(46)-C(47)-C(48)	-1.3(4)
C(46)-C(47)-C(48)-C(49)	-0.7(4)
C(46)-C(47)-C(48)-N(6)	176.8(3)
C(47)-C(48)-C(49)-C(50)	2.0(4)
N(6)-C(48)-C(49)-C(50)	-175.5(3)
C(48)-C(49)-C(50)-C(45)	-1.4(4)
C(46)-C(45)-C(50)-C(49)	-0.6(4)
C(42)-C(45)-C(50)-C(49)	177.2(3)
C(56)-C(51)-C(52)-C(53)	-3.4(5)
N(6)-C(51)-C(52)-C(53)	176.8(3)
C(51)-C(52)-C(53)-C(54)	0.0(5)
C(52)-C(53)-C(54)-C(55)	2.9(5)
C(53)-C(54)-C(55)-C(56)	-2.2(6)
C(54)-C(55)-C(56)-C(51)	-1.3(6)
C(52)-C(51)-C(56)-C(55)	4.0(5)
N(6)-C(51)-C(56)-C(55)	-176.1(3)
C(62)-C(57)-C(58)-C(59)	0.2(5)
N(6)-C(57)-C(58)-C(59)	179.4(3)
C(57)-C(58)-C(59)-C(60)	0.5(6)
C(58)-C(59)-C(60)-C(61)	-0.9(6)
C(59)-C(60)-C(61)-C(62)	0.6(6)
C(58)-C(57)-C(62)-C(61)	-0.4(5)
N(6)-C(57)-C(62)-C(61)	-179.6(3)
C(60)-C(61)-C(62)-C(57)	0.0(5)
N(5)-C(63)-C(64)-C(65)	-175.9(3)
C(63)-C(64)-C(65)-C(66)	-178.5(3)
C(64)-C(65)-C(66)-C(67)	67.6(4)
C(65)-C(66)-C(67)-C(68)	167.6(3)

C(66)-C(67)-C(68)-C(69)	-179.3(4)
C(67)-C(68)-C(69)-C(70)	177.6(4)
N(2)-C(1)-N(1)-C(3)	1.5(3)
N(2)-C(1)-N(1)-Zn(1)	169.0(2)
C(2)-C(3)-N(1)-C(1)	-0.6(3)
C(4)-C(3)-N(1)-C(1)	-179.0(3)
C(2)-C(3)-N(1)-Zn(1)	-165.9(2)
C(4)-C(3)-N(1)-Zn(1)	15.7(4)
N(1)-C(1)-N(2)-C(2)	-1.7(4)
N(1)-C(1)-N(2)-C(28)	174.5(3)
C(3)-C(2)-N(2)-C(1)	1.3(4)
C(3)-C(2)-N(2)-C(28)	-175.0(3)
C(29)-C(28)-N(2)-C(1)	83.6(4)
C(29)-C(28)-N(2)-C(2)	-100.8(4)
C(12)-C(13)-N(3)-C(16)	-34.6(5)
C(14)-C(13)-N(3)-C(16)	146.4(4)
C(12)-C(13)-N(3)-C(22)	154.9(4)
C(14)-C(13)-N(3)-C(22)	-24.1(5)
C(17)-C(16)-N(3)-C(13)	-41.7(5)
C(21)-C(16)-N(3)-C(13)	139.8(3)
C(17)-C(16)-N(3)-C(22)	129.0(4)
C(21)-C(16)-N(3)-C(22)	-49.5(5)
C(23)-C(22)-N(3)-C(13)	126.6(4)
C(27)-C(22)-N(3)-C(13)	-55.5(5)
C(23)-C(22)-N(3)-C(16)	-44.3(5)
C(27)-C(22)-N(3)-C(16)	133.5(4)
N(5)-C(36)-N(4)-C(38)	0.3(3)
N(5)-C(36)-N(4)-Zn(1)	163.86(18)
C(37)-C(38)-N(4)-C(36)	-0.2(3)
C(39)-C(38)-N(4)-C(36)	-179.7(3)
C(37)-C(38)-N(4)-Zn(1)	-161.4(2)
C(39)-C(38)-N(4)-Zn(1)	19.2(4)
N(4)-C(36)-N(5)-C(37)	-0.2(3)
N(4)-C(36)-N(5)-C(63)	176.3(3)
C(38)-C(37)-N(5)-C(36)	0.0(3)
C(38)-C(37)-N(5)-C(63)	-176.4(3)
C(64)-C(63)-N(5)-C(36)	127.8(3)
C(64)-C(63)-N(5)-C(37)	-56.4(4)
C(56)-C(51)-N(6)-C(48)	-163.6(3)
C(52)-C(51)-N(6)-C(48)	16.2(4)
C(56)-C(51)-N(6)-C(57)	31.5(4)

C(52)-C(51)-N(6)-C(57)	-148.7(3)
C(49)-C(48)-N(6)-C(51)	53.8(4)
C(47)-C(48)-N(6)-C(51)	-123.7(3)
C(49)-C(48)-N(6)-C(57)	-140.9(3)
C(47)-C(48)-N(6)-C(57)	41.6(4)
C(58)-C(57)-N(6)-C(51)	35.1(4)
C(62)-C(57)-N(6)-C(51)	-145.7(3)
C(58)-C(57)-N(6)-C(48)	-129.9(3)
C(62)-C(57)-N(6)-C(48)	49.2(4)

6. Fluorescence decay curves

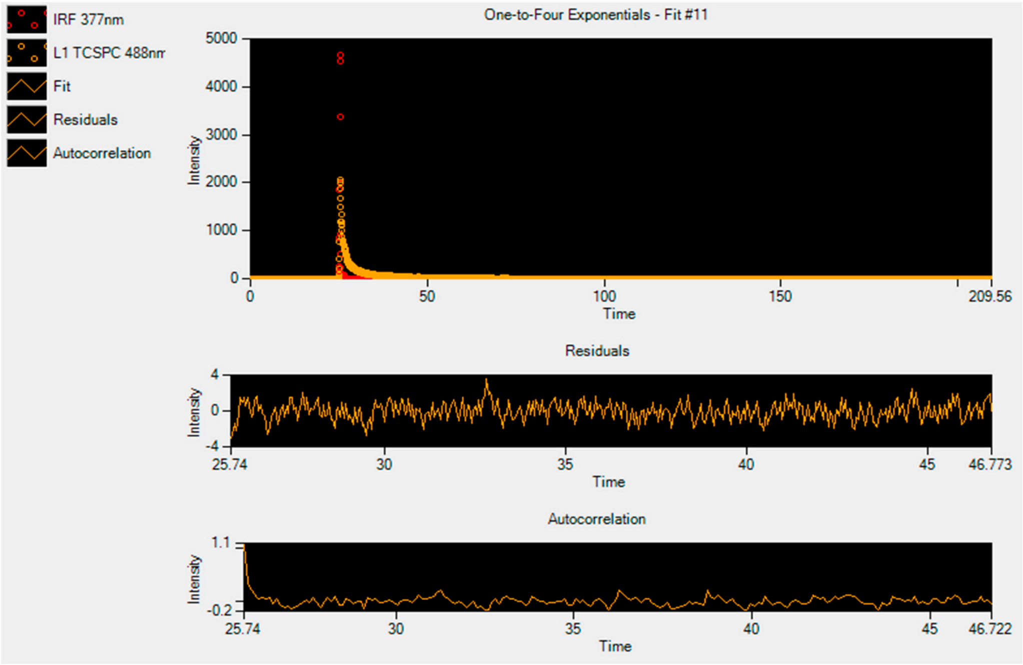

Figure S13: The fluorescence decay curve of **ImL1**.

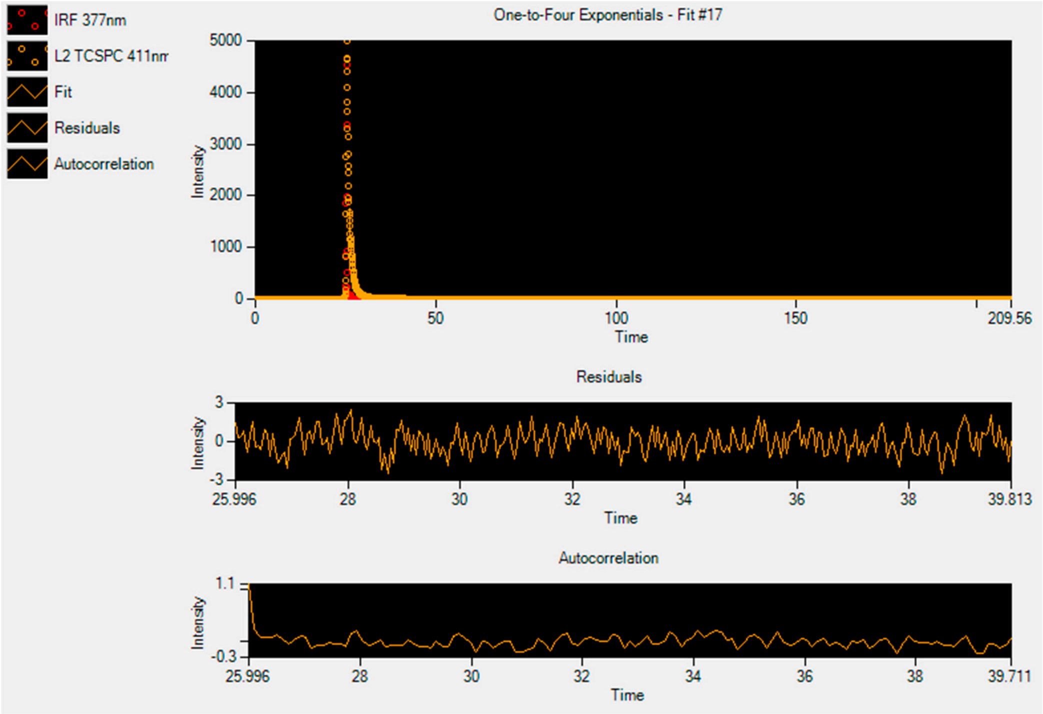

Figure S14: The fluorescence decay curve of **ImL2**.

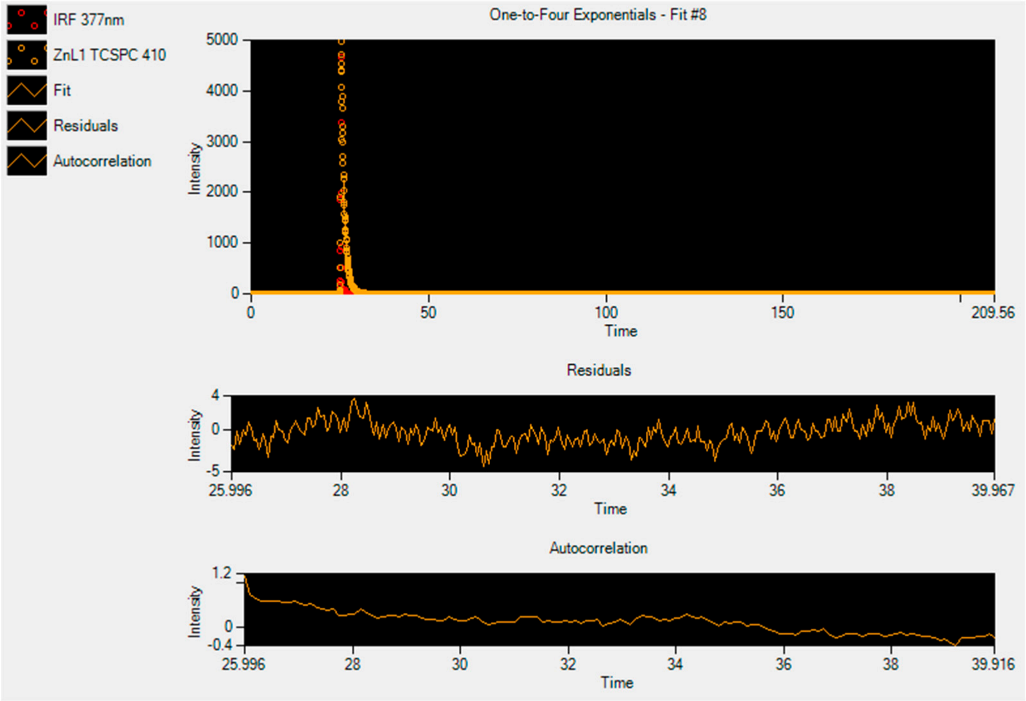

Figure S15: The fluorescence decay curve of **$ZnCl_2(ImL1)_2$**.

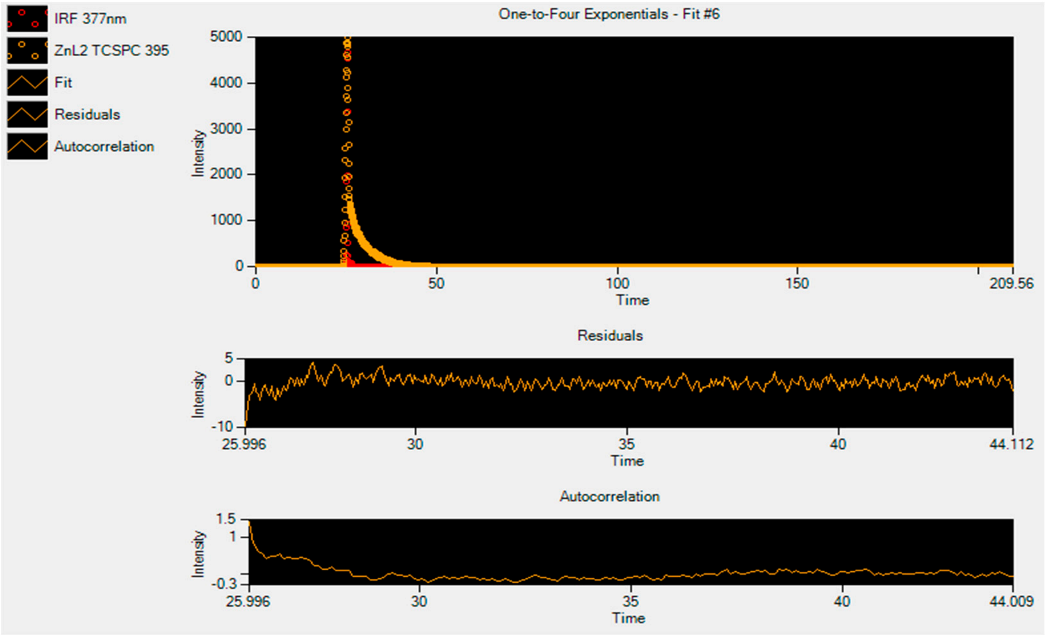

Figure S16: The fluorescence decay curve of **$ZnCl_2(ImL2)_2$**.

Disclaimer/Publisher's Note: The statements, opinions and data contained in all publications are solely those of the individual author(s) and contributor(s) and not of MDPI and/or the editor(s). MDPI and/or the editor(s) disclaim responsibility for any injury to people or property resulting from any ideas, methods, instructions or products referred to in the content.
